# Supplementary material for: Molecular Rules Underpinning Enhanced Affinity Binding of Human T Cell Receptors Engineered for Immunotherapy
Source: Mol Ther Oncolytics. 2020 Jul 31;18:443–56. doi: 10.1016/j.omto.2020.07.008 (PMC7452143; doi:10.1016/j.omto.2020.07.008)
Supplement: Document S1. Figures S1–S7, Tables S1–S7, and Supplemental Materials and Methods [file mmc1.pdf]

OMTO, Volume *18*

Supplemental Information

Molecular Rules Underpinning Enhanced Affinity Binding of Human T Cell Receptors Engineered for Immunotherapy

Rory M. Crean, Bruce J. MacLachlan, Florian Madura, Thomas Whalley, Pierre J. Rizkallah, Christopher J. Holland, Catriona McMurran, Stephen Harper, Andrew Godkin, Andrew K. Sewell, Christopher R. Pudney, Marc W. van der Kamp, and David K. Cole

Title:

Molecular rules underpinning enhanced affinity binding of human T cell receptors engineered for immunotherapy

Authors and Affiliations:

Rory M. Crean[1,2], Bruce J. MacLachlan[3†], Florian Madura[3], Thomas Whalley[3], Pierre J. Rizkallah[3], Christopher J. Holland[4], Catriona McMurran[4], Stephen Harper[4], Andrew Godkin[3], Andrew K. Sewell[3], Christopher R. Pudney[1,5], Marc W. van der Kamp[6*], and David K. Cole[3,4*]

[1]Department of Biology and Biochemistry, University of Bath, Bath, BA2 7AY, UK.
[2]Doctoral Training Centre in Sustainable Chemical Technologies, University of Bath, Bath, BA2 7AY, UK.
[3]Division of Infection & Immunity, Cardiff University, CF14 4XN, UK.
[4]Immunocore Ltd., Abingdon OX14 4RY, UK
[5]Centre for Therapeutic Innovation, University of Bath, Bath, BA2 7AY, UK.
[6]School of Biochemistry, University of Bristol, Biomedical Sciences Building, University Walk, Bristol, BS8 1TD, UK.

†Present address: Monash Biomedicine Discovery Institute, 19 Innovation Walk, Clayton, Victoria, 3800, Australia

*These authors contributed equally to the study

To whom correspondence should be addressed: Dr Marc W. van der Kamp Email: marc.vanderkamp@bristol.ac.uk, and Dr David K. Cole, E-mail: david.cole@immunocore.com.

Supplementary Figures

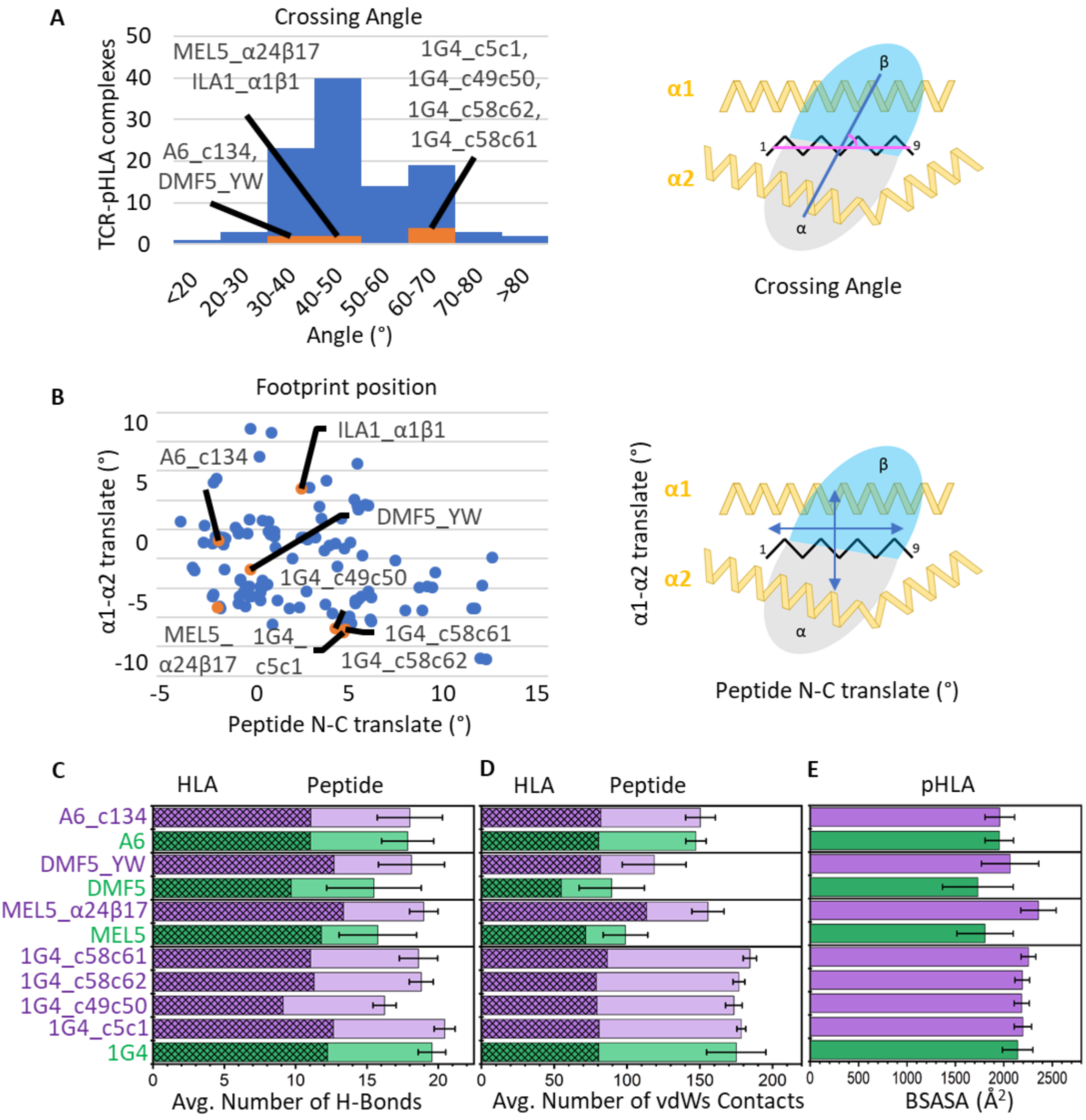

Supplementary Figure S1: (**A**) Distribution of TCR crossing angles of all published TCR-pHLA complexes are plotted in blue. Affinity enhanced TCR-pHLA complexes are labelled and shown in the plot in orange. (**B**) Footprint position of all published TCR-pHLA complexes are plotted in blue. Affinity enhanced TCR-pHLA complexes are labelled and shown in the plot in orange. (**C-E**) Differences in the average number of HBs (**C**), vdWs contacts (**D**) and the BSASA (**E**) from our MD simulations. All wildtype TCRs are coloured in green and all affinity enhanced TCRs are coloured in purple. Bars for the HBs and vdWs contacts are divided in two based on TCR-HLA interactions (darker colour and hashed bars) and peptide-TCR interactions (lighter colour). The totals obtained are from 10 independent 100 ns long MD simulations of each TCR-pHLA complex (using the last 90 ns of each simulation). Error bars plotted for A and B are the standard deviation of the averages from the 10 replicas. Error bars plotted for C are the standard deviation obtained from combining all snapshots from all replicas together.

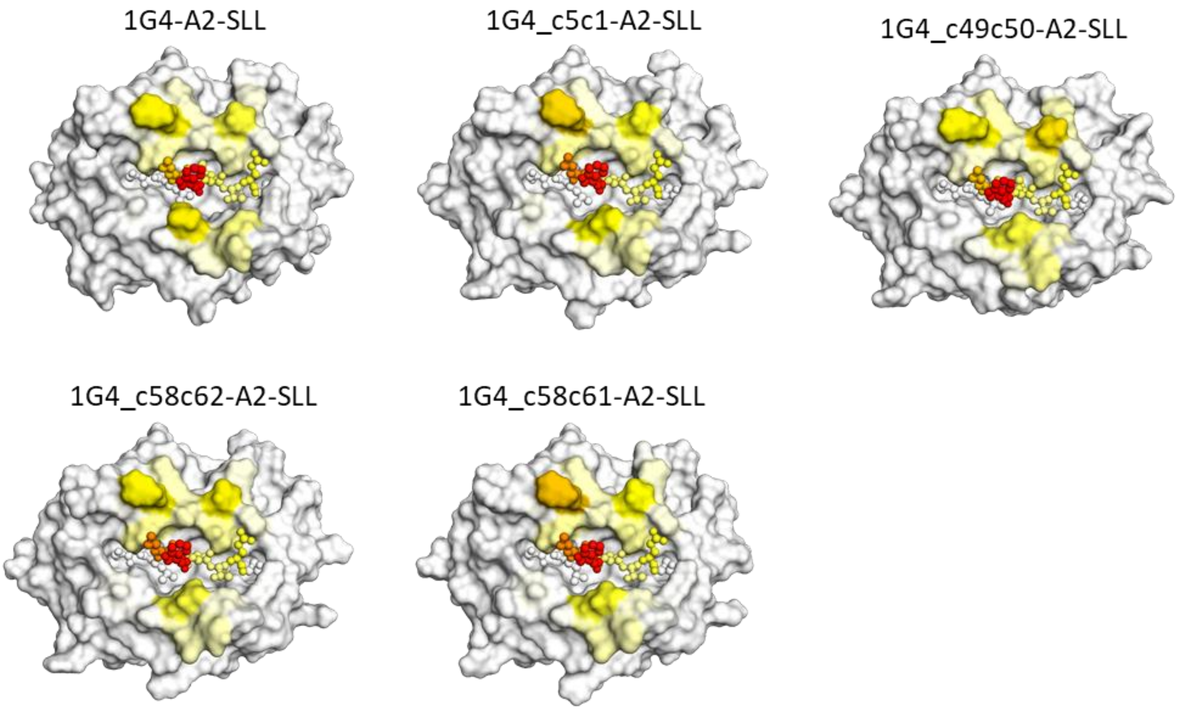

Supplementary Figure S2: Surface plots of the pHLA (peptide shown as sticks) with each structure colour mapped according the average number of vdWs contacts formed between the given residue and all five 1G4 affinity enhanced TCRs studied in this manuscript. Colour mapping was performed from white (no contacts) through yellow and orange to red (highest number of contacts observed for each of the pairs of TCR-pHLAs studied). All pHLA structures are shown in the same orientation, such that the peptide N-terminus is left and the C-terminus is right.

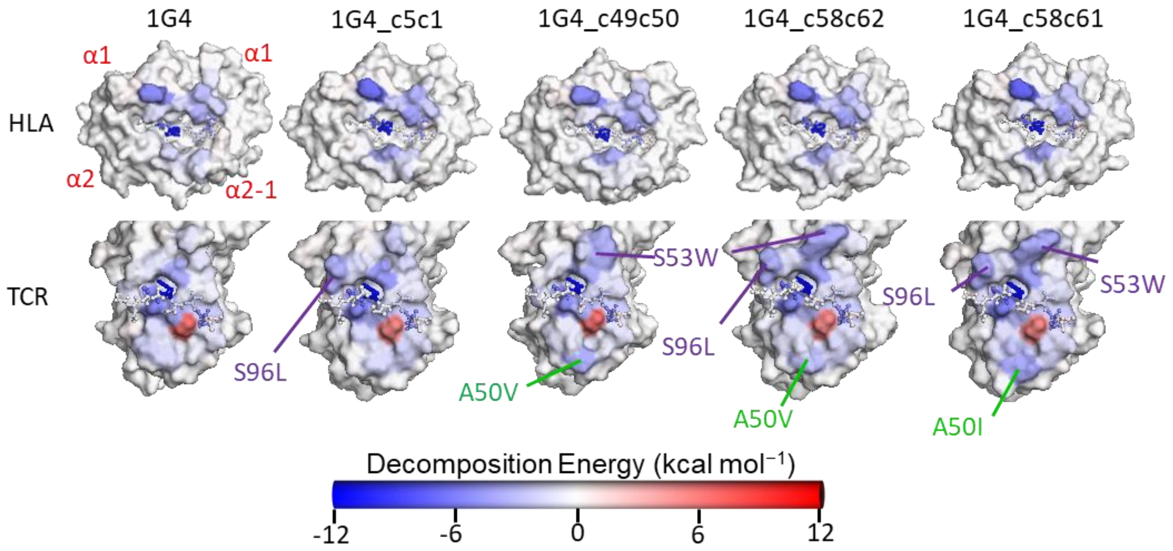

Supplementary Figure S3: Changes in the energetic footprint between the wildtype 1G4 and affinity enhanced 1G4 TCRs. For all TCR-pHLA complexes, the HLA (top) and TCR (bottom) structures are plotted as surfaces with the peptide shown in both structures as sticks. All plots are colour mapped according to the MMGBSA per residue decomposition results, going from blue (favours binding) to white (no preference) to red (disfavours binding). All pHLA and TCR structures are shown in the same orientation, such that the peptide N-terminus is left and the C-terminus right. Several mutations sites are indicated on the affinity enhanced variants (purple labels: CDRα mutations; green labels: CDRβ mutations).

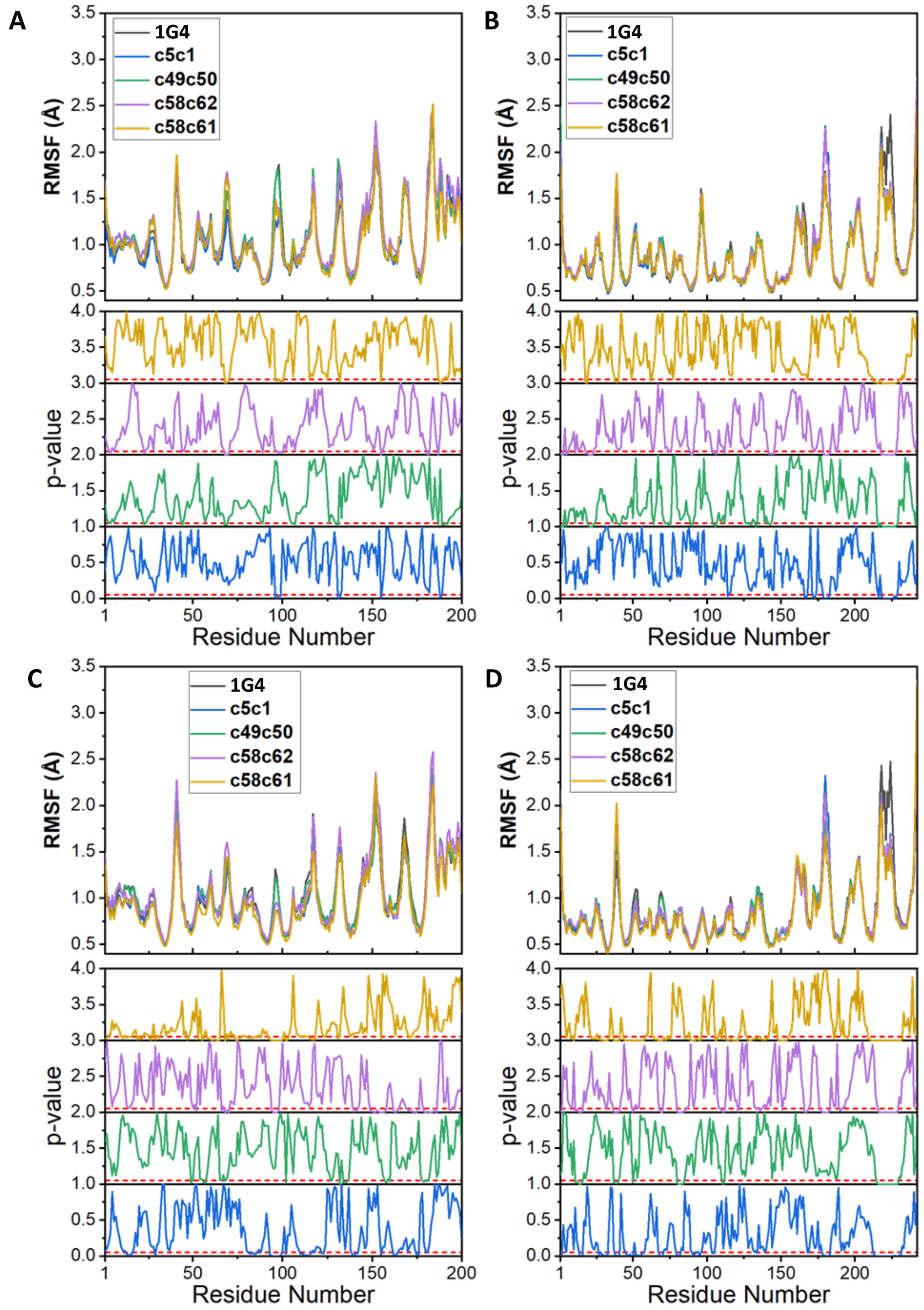

Supplementary Figure S4: Cα RMSF values for all Apo (A + B) and pHLA bound (C + D) 1G4 TCRs simulated. The α-chain RMSFs for the apo and pHLA bound simulations are plotted in panels A and C respectively, whilst the β-chain RMSFs for the apo and pHLA bound simulations are plotted in panels B and D respectively. Below each plot is the p-value obtained from a two-sample t-test between the wildtype and each affinity enhanced 1G4 TCR (following the same colour scheme). A red dotted line is plotted at a p-value of 0.05, which is the cut-off used to determine significance.

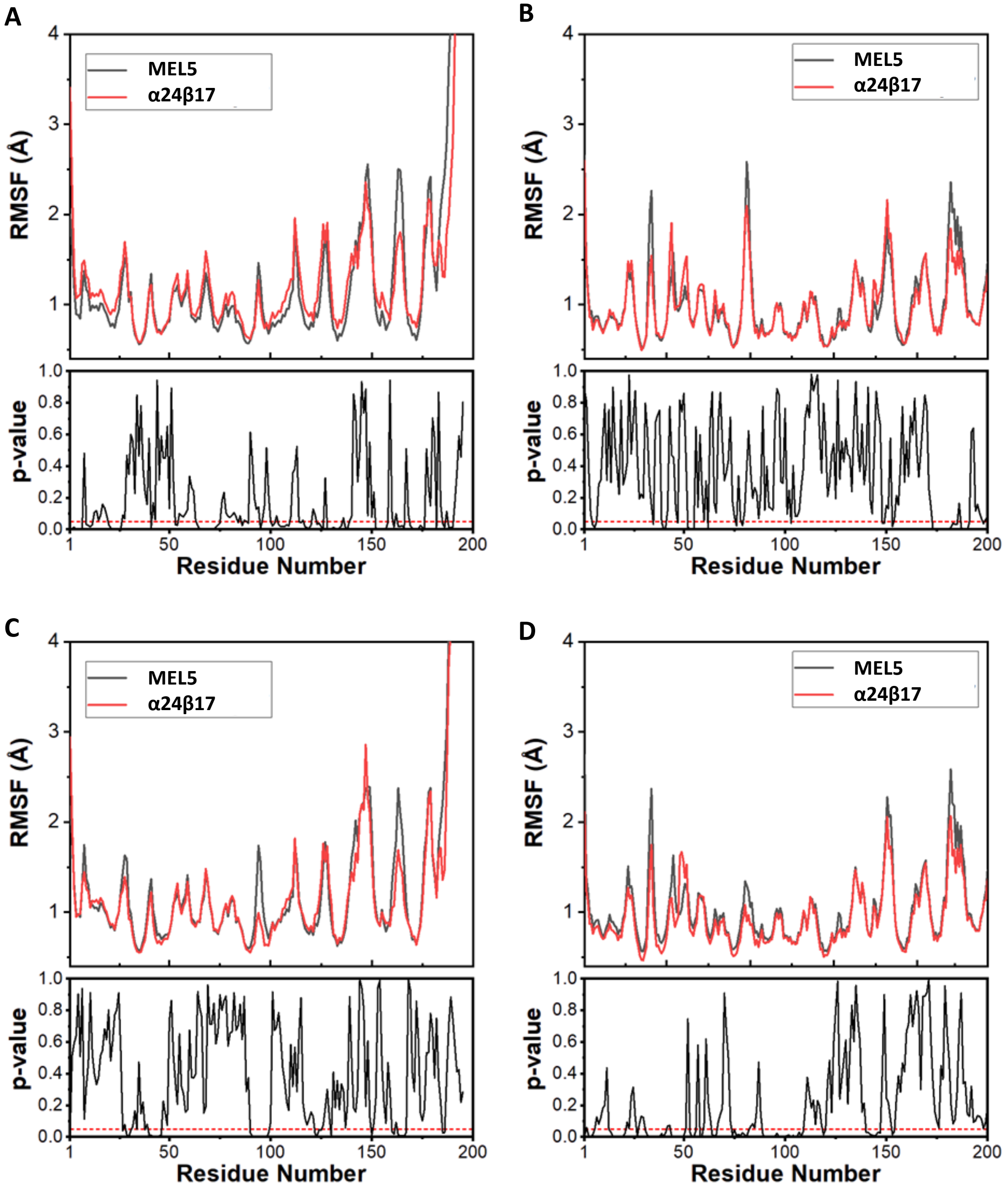

Supplementary Figure S5: Cα RMSF values for both Apo (A + B) and pHLA bound (C + D) MEL5 TCRs simulated. The α-chain RMSFs for the apo and pHLA bound simulations are plotted in panels A and C respectively, whilst the β-chain RMSFs for the apo and pHLA bound simulations are plotted in panels B and D respectively. Below each plot is the p-value obtained from a two-sample t-test between the wildtype and the affinity enhanced MEL5 TCRs. A red dotted line is plotted at a p-value of 0.05, which is the cut-off used to determine significance.

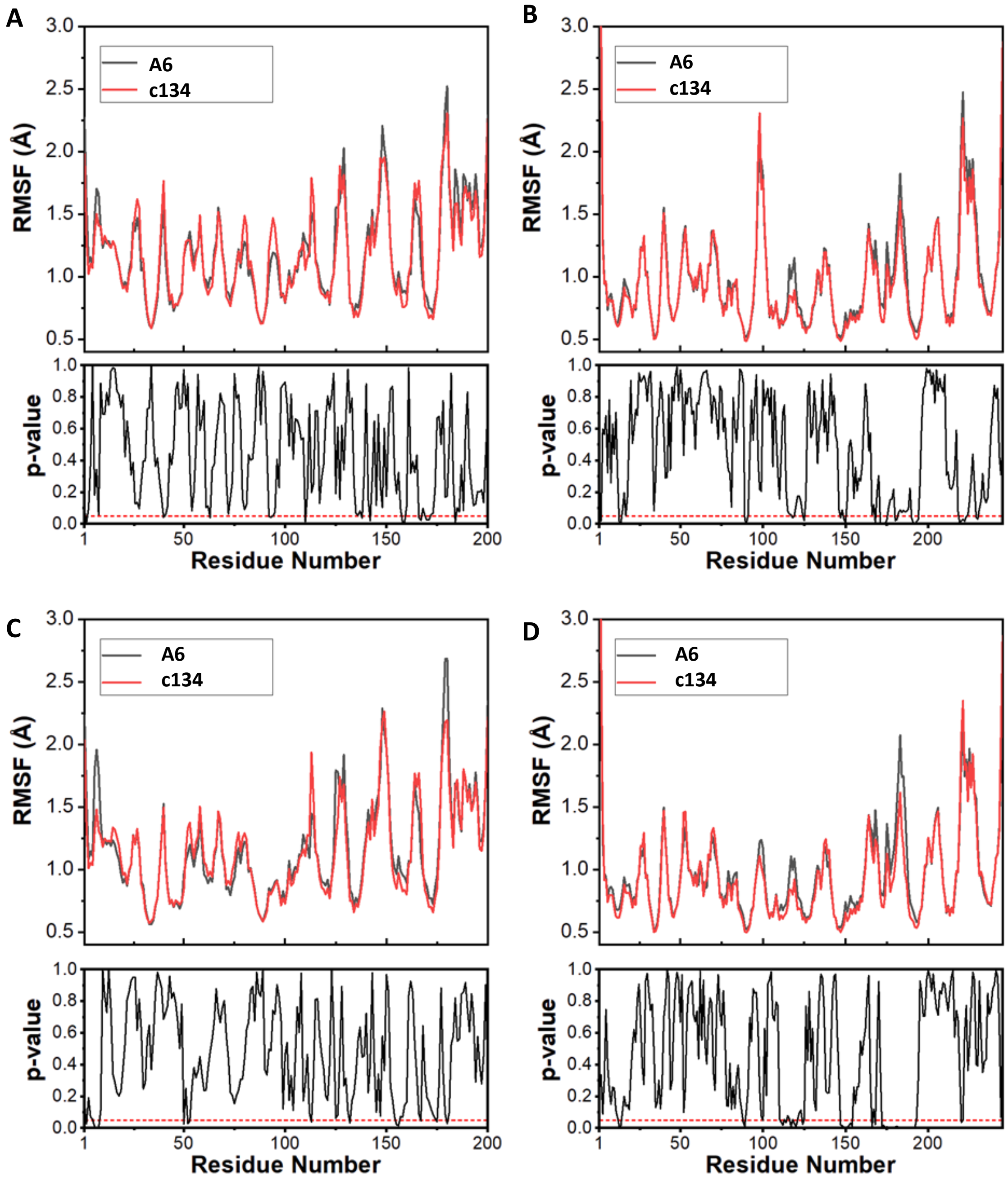

Supplementary Figure S6: Cα RMSF values for both Apo (A + B) and pHLA bound (C + D) Tax A6 TCRs simulated. The α-chain RMSFs for the apo and pHLA bound simulations are plotted in panels A and C respectively, whilst the β-chain RMSFs for the apo and pHLA bound simulations are plotted in panels B and D respectively. Below each plot is the p-value obtained from a two-sample t-test between the wildtype and the affinity enhanced A6 TCRs. A red dotted line is plotted at a p-value of 0.05, which is the cut-off used to determine significance.

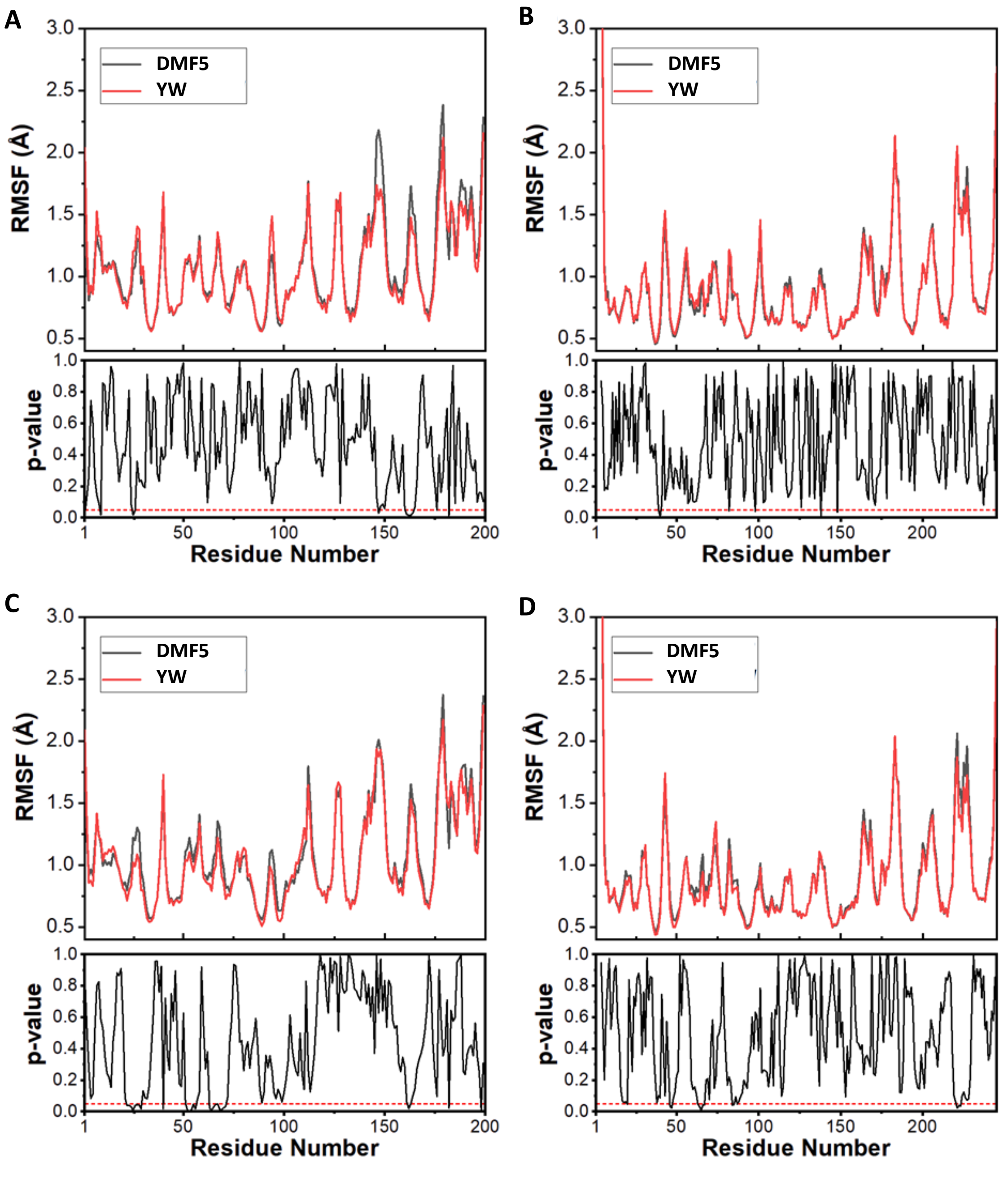

Supplementary Figure S7: Cα RMSF values for both Apo (A + B) and pHLA bound (C + D) DMF5 TCRs simulated. The α-chain RMSFs for the apo and pHLA bound simulations are plotted in panels A and C respectively, whilst the β-chain RMSFs for the apo and pHLA bound simulations are plotted in panels B and D respectively. Below each plot is the p-value obtained from a two-sample t-test between the wildtype and the affinity enhanced DMF5 TCRs. A red dotted line is plotted at a p-value of 0.05, which is the cut-off used to determine significance.

Supplementary Tables

Supplementary Table S1. Data collection and refinement statistics

* One crystal was used for determining each structure.
* Figures in brackets refer to outer resolution shell, where applicable.
[1] Coordinate Estimated Standard Uncertainty in (Å), based on maximum likelihood statistics.

**Data Collection**	**MEL5_α24β17-A2-EAA**
Accession Code	6TMO
Beamtime proposal	mx6232-3
Diamond Beamline	I02
Wavelength	0.9795
**Crystal Data**	
a, b, c (Å)	121.49, 121.49, 82.68
α, β, γ (°)	90.0, 90.0, 90.0
Space group	$P\ 4_3$
Resolution (Å)	2.10 – 54.33
Outer shell	2.10 – 2.15
R-merge (%)	8.4 (71.3)
R-meas (%)	9.6 (80.7)
R-pim (%)	3.3 (27.7)
CC1/2	n/a (n/a)
I / σ(I)	16.0 (3.6)
Completeness (%)	99.4 (99.0)
Multiplicity	8.3 (8.4)
Total Measurements	581,617 (42,829)
Unique Reflections	69,920 (5,110)
Wilson B-factor ($Å^2$)	33.3
**Refinement Statistics**	
Non-H Atoms	7,167
R-work reflections	66,386
R-free reflections	3,532
R-work/R-free	0.173 / 0.209
**rms deviations**	
Bond lengths (Å)	0.016
Bond Angles (°)	1.556
[1]Coordinate error	0.093
Mean B value ($Å^2$)	40.3
**Ramachandran Statistics**	
Favoured/allowed/Outliers	775 / 24 / 6
%	96.3 / 3.0 / 0.7

Supplementary Table S2. PDB IDs and histidine tautomerisation state assignments for all 1G4 MD simulations.

[a], All Apo-TCR simulations used the same tautomerisation states as those in the TCR-pHLA simulations.

[b], HID corresponds to a histidine residue which is singly protonated on its Nδ1 nitrogen.

[c], HIE corresponds to a histidine residue which is singly protonated on their Nε2 nitrogen.

TCR-pHLA System[a]	HID Tautomerisation States[b]	HIE Tautomerisation States[c]
**1G4**<br>PDB: 2BNR	**HLA:** 3, 70, 74, 93, 114, 145, 260.<br>**β2m:**<br>**CDRα:**<br>**CDRβ:** 151.	**HLA:** 151, 188, 191, 192, 197, 263.<br>**β2m:** 13, 31, 51, 84.<br>**CDRα:** 112.<br>**CDRβ:** 27, 45, 134, 164, 204.
**1G4_c5c1**<br>PDB: 2PYE	**HLA:** 3, 70, 74, 93, 114, 145, 260.<br>**β2m:**<br>**CDRα:**<br>**CDRβ:** 151.	**HLA:** 151, 188, 191, 192, 197, 263.<br>**β2m:** 13, 31, 51, 84.<br>**CDRα:** 112.<br>**CDRβ:** 27, 45, 134, 164, 204.
**1G4_c49c50**<br>PDB: 2F53	**HLA:** 3, 70, 74, 93, 114, 145, 260.<br>**β2m:**<br>**CDRα:**<br>**CDRβ:** 151.	**HLA:** 151, 188, 191, 192, 197, 263.<br>**β2m:** 13, 31, 51, 84.<br>**CDRα:** 112.<br>**CDRβ:** 27, 45, 134, 164, 204.
**1G4_c58c62**<br>PDB: 2P5W	**HLA:** 3, 70, 74, 93, 114, 145, 260.<br>**β2m:**<br>**CDRα:**<br>**CDRβ:** 151.	**HLA:** 151, 188, 191, 192, 197, 263.<br>**β2m:** 13, 31, 51, 84.<br>**CDRα:** 112.<br>**CDRβ:** 27, 45, 134, 164, 204.
**1G4_c58c61**<br>PDB: 2P5E	**HLA:** 3, 70, 74, 93, 114, 145, 260.<br>**β2m:**<br>**CDRα:**<br>**CDRβ:** 151.	**HLA:** 151, 188, 191, 192, 197, 263.<br>**β2m:** 13, 31, 51, 84.<br>**CDRα:** 112.<br>**CDRβ:** 27, 45, 134, 164, 204.

Supplementary Table S3. PDB IDs and histidine tautomerisation state assignments for the MD simulations of the A6, DMF5 and MEL5 and affinity enhanced TCRs.
[a], All Apo-TCR simulations used the same tautomerisation states as those in the TCR-pHLA simulations.
[b], HID corresponds to a histidine residue which is singly protonated on its Nδ1 nitrogen.
[c], HIE corresponds to a histidine residue which is singly protonated on their Nε2 nitrogen.

TCR-pHLA System[a]	HID Tautomerisation States[b]	HIE Tautomerisation States[c]
**DMF5**<br>PDB: 3QDG	**HLA:** 3, 70, 74, 93, 114, 151, 191, 192, 260.<br>**β2m:** 51.<br>**CDRα:**<br>**CDRβ:** 32, 50, 138, 168.	**HLA:** 145, 188, 197, 263.<br><br>**β2m:** 13, 31, 84.<br>**CDRα:**<br>**CDRβ:** 155, 208.
**DMF5_YW**<br>PDB: 4L3E	**HLA:** 3, 70, 74, 93, 114, 151, 191, 192, 260.<br>**β2m:** 51.<br>**CDRα:**<br>**CDRβ:** 32, 50, 138, 168.	**HLA:** 145, 188, 197, 263.<br><br>**β2m:** 13, 31, 84.<br>**CDRα:**<br>**CDRβ:** 155, 208.
**A6**<br>PDB: 1AO7	**HLA:** 3, 70, 74, 93, 114.<br><br>**β2m:**<br>**CDRα:**<br>**CDRβ:**	**HLA:** 145, 151, 188, 191, 192, 260, 263.<br>**β2m:** 13,31, 51, 84.<br>**CDRα:**<br>**CDRβ:** 29, 47, 139, 156, 169, 209.
**A6_c134**<br>PDB: 4FTV	**HLA:** 3, 70, 74, 93, 114.<br><br>**β2m:**<br>**CDRα:**<br>**CDRβ:**	**HLA:** 145, 151, 188, 191, 192, 260, 263.<br>**β2m:** 13,31, 51, 84.<br>**CDRα:**<br>**CDRβ:** 29, 47, 139, 156, 169, 209.
**MEL5**<br>PDB: 3HG1	**HLA:** 3, 70, 74, 114.<br><br>**β2m:**<br>**CDRα:**<br>**CDRβ:** 5, 207.	**HLA:** 93, 145, 151, 188, 191, 192, 197, 260, 263.<br>**β2m:** 13, 31, 51, 84.<br>**CDRα:** 71.<br>**CDRβ:** 137, 154, 167.
**MEL5_α24β17**<br>PDB: 4JFF	**HLA:** 3, 70, 74, 114.<br><br>**β2m:**<br>**CDRα:**<br>**CDRβ:** 5, 207.	**HLA:** 93, 145, 151, 188, 191, 192, 197, 260, 263.<br>**β2m:** 13, 31, 51, 84.<br>**CDRα:** 71.<br>**CDRβ:** 137, 154, 167.

Supplementary Table S4: Average number of vdWs contacts formed between the TCR to each pHLA residue for all 1G4 simulations. For each TCR-pHLA complex the 10 pHLA residues with the greatest number of contacts are coloured red, with their rank provided in brackets.

	TCR-pHLA Complex				
**Residue**	**1G4**	**1G4_c5c1**	**1G4_c49c50**	**1G4_c58c62**	**1G4_c58c61**
**HLA: 19**	0.0	0.0	0.2	0.3	0.7
**HLA: 62**	1.4	0.7	1.5	0.5	0.6
**HLA: 65**	14.6 (4)	19.2 (3)	16.3 (4)	14.3 (4)	19.5 (3)
**HLA: 66**	4.2	6.0 (9)	2.4	6.1 (9)	5.6 (10)
**HLA: 68**	2.4	2.1	4.2	3.7	3.7
**HLA: 69**	2.5	3.7	2.4	4.2	3.5
**HLA: 71**	0.0	0.0	0.1	0.1	0.0
**HLA: 72**	11.6 (6)	13.3 (4)	16.9 (3)	15.4 (3)	14.8 (4)
**HLA: 73**	4.0	5.9 (10)	4.7 (10)	6.0 (10)	6.0 (9)
**HLA: 75**	1.7	0.5	3.6	1.8	2.0
**HLA: 76**	0.8	1.9	1.4	1.9	1.9
**HLA: 146**	0.1	0.0	0.0	0.2	0.1
**HLA: 149**	0.0	0.0	0.0	0.3	0.2
**HLA: 150**	7.1 (9)	5.0	4.0	2.4	2.4
**HLA: 151**	4.9 (10)	5.0	6.5 (8)	5.2	4.8
**HLA: 152**	0.3	0.0	0.0	0.0	0.0
**HLA: 154**	2.1	2.1	1.4	4.0	4.9
**HLA: 155**	16.4 (3)	11.9 (6)	11.5 (6)	9.9 (6)	11.7 (6)
**HLA: 158**	0.0	0.0	0.7	0.4	0.6
**HLA: 163**	0.0	1.0	0.0	0.7	1.0
**Pep: 4**	21.1 (2)	27.8 (2)	21.5 (2)	28.3 (2)	28.0 (2)
**Pep: 5**	44.4 (1)	42.7 (1)	44.4 (1)	42.4 (1)	42.8 (1)
**Pep: 6**	9.4 (7)	7.8 (7)	9.5 (7)	7.7 (7)	7.7 (7)
**Pep: 7**	7.1 (8)	7.4 (8)	6.2 (9)	7.3 (8)	6.7 (8)
**Pep: 8**	12.5 (5)	13.1 (5)	12.0 (5)	12.7 (5)	12.7 (5)

Supplementary Table S5: Average number of hydrogen bonds formed between the TCR to each pHLA residue for all 1G4 simulations. For each TCR-pHLA complex the 10 pHLA residues with the greatest number of contacts are coloured red, with their rank provided in brackets.

	TCR-pHLA Complex				
**Residue**	**1G4**	**1G4_c5c1**	**1G4_c49c50**	**1G4_c58c62**	**1G4_c58c61**
**HLA: 19**	0.1	0.3	0.2	0.2	0.4
**HLA: 43**	0.0	0.0	0.1	0.1	0.0
**HLA: 58**	0.0	0.1	0.0	0.1	0.1
**HLA: 61**	0.0	0.0	0.1	0.0	0.1
**HLA: 65**	2.3 (2)	3.2 (1)	2.3 (2)	2.9 (1)	3.2 (1)
**HLA: 66**	0.5	0.7	0.4	0.7 (10)	0.7
**HLA: 68**	0.4	0.6	0.5 (10)	0.4	0.6
**HLA: 70**	0.0	0.1	0.0	0.0	0.1
**HLA: 71**	0.0	0.0	0.0	0.1	0.0
**HLA: 72**	1.7 (4)	1.1 (8)	2.2 (3)	1.0 (7)	0.9 (8)
**HLA: 73**	0.6	1.6 (5)	0.7 (9)	1.6 (4)	1.6 (5)
**HLA: 75**	0.4	0.3	0.4	0.3	0.2
**HLA: 80**	0.0	0.0	0.0	0.1	0.0
**HLA: 146**	0.0	0.3	0.3	0.4	0.3
**HLA: 149**	0.1	0.1	0.1	0.2	0.2
**HLA: 150**	1.5 (6)	1.1 (7)	0.2	0.5	0.6
**HLA: 151**	1.0 (8)	0.8	0.3	0.7	0.7 (10)
**HLA: 154**	0.2	0.3	0.0	0.4	0.4
**HLA: 155**	2.1 (3)	1.8 (4)	1.2 (5)	1.2 (5)	1.6 (4)
**HLA: 163**	0.1	0.0	0.0	0.0	0.0
**Pep: 1**	0.0	0.1	0.0	0.0	0.1
**Pep: 4**	1.1 (7)	1.4 (6)	1.0 (6)	1.2 (6)	1.3 (6)
**Pep: 5**	1.0 (9)	0.9 (10)	0.9 (8)	0.9 (9)	0.9 (9)
**Pep: 6**	1.7 (5)	1.8 (3)	1.8 (4)	1.8 (3)	1.8 (3)
**Pep: 7**	1.0 (10)	1.0 (9)	1.0 (7)	1.0 (8)	1.0 (7)
**Pep: 8**	2.5 (1)	2.6 (2)	2.5 (1)	2.6 (2)	2.6 (2)

Supplemetary Table S6: Average number of vdWs contacts formed between the TCR to each pHLA residue for our simulations of wildtype and affinity enhanced DMF5, MEL5 and Tax A6. For each TCR-pHLA complex the 10 pHLA residues with the greatest number of contacts are coloured red, with their rank provided in brackets.

	TCR-pHLA Complex					
**Residue**	**DMF5**	**DMF5_ YW**	**MEL5**	**MEL5_ α24β17**	**A6**	**A6_ c134**
**HLA: 43**	0.0	0.0	0.4	0.0	0.0	0.0
**HLA: 55**	3.0	3.5	0.0	0.0	2.7	3.6
**HLA: 56**	0.0	0.2	0.0	0.0	0.0	0.0
**HLA: 58**	0.1	2.9	0.3	2.0	0.1	0.5
**HLA: 59**	1.2	2.3	0.0	0.3	0.4	1.0
**HLA: 61**	0.0	0.9	0.0	0.0	0.0	0.0
**HLA: 62**	0.0	3.2	1.6	0.2	0.0	0.0
**HLA: 63**	0.0	0.3	0.0	0.0	0.0	0.0
**HLA: 65**	7.1 (4)	8.5 (3)	13.9 (1)	19.9 (2)	17.1 (3)	17.1 (2)
**HLA: 66**	4.1 (9)	8.3 (4)	1.8	4.0	8.3 (6)	9.1 (6)
**HLA: 68**	0.0	0.0	2.2	1.6	2.7	2.8
**HLA: 69**	5.6 (7)	7.1 (5)	3.6 (9)	3.1	5.7 (8)	5.7 (9)
**HLA: 70**	0.1	0.5	2.3	1.4	0.0	0.0
**HLA: 72**	10.4 (1)	12.2 (1)	8.0 (4)	25.4 (1)	2.2	2.5
**HLA: 73**	1.3	1.5	2.9	1.7	2.2	2.1
**HLA: 75**	1.3	1.7	0.9	6.6 (8)	0.0	0.0
**HLA: 76**	1.7	2.1	1.3	4.2 (10)	0.0	0.0
**HLA: 145**	0.0	0.1	0.0	0.0	0.0	0.0
**HLA: 146**	0.0	0.8	0.0	0.0	0.0	0.0
**HLA: 149**	0.0	0.7	0.0	0.0	0.8	0.2
**HLA: 150**	0.8	4.8 (9)	0.0	0.1	5.1 (9)	8.4 (8)
**HLA: 151**	0.2	0.0	1.0	0.7	3.5	4.6 (10)
**HLA: 152**	0.0	0.0	0.0	0.0	0.5	1.2
**HLA: 154**	0.1	0.6	4.2 (8)	6.3 (9)	1.2	1.9
**HLA: 155**	7.6 (3)	5.8 (8)	9.2 (2)	10.4 (3)	13.3 (4)	12.9 (4)
**HLA: 157**	0.0	0.0	0.0	8.0 (6)	0.5	0.1
**HLA: 158**	2.1	1.8	1.4	2.2	2.3	1.6
**HLA: 159**	1.9	1.6	0.7	1.4	0.9	0.8
**HLA: 161**	0.0	0.0	0.0	2.2	0.0	0.1
**HLA: 162**	0.0	0.0	0.0	0.3	0.0	0.1
**HLA: 163**	1.7	2.0	3.5 (10)	2.8	2.4	2.6
**HLA: 166**	1.0	1.8	4.3 (7)	0.4	4.2 (10)	3.6
**HLA: 167**	1.4	2.7	0.3	4.2	2.2	2.0
**HLA: 170**	0.7	1.7	1.5	0.7	2.1	1.0
**Pep: 1**	6.5 (5)	6.1 (6)	0.8	4.2	2.8	3.2
**Pep: 2**	2.3	2.0	0.4	1.2	1.6	1.2
**Pep: 3**	1.7	1.7	1.2	2.1	0.2	0.0
**Pep: 4**	8.4 (2)	9.1 (2)	5.5 (5)	8.7 (5)	9.5 (5)	8.9 (7)
**Pep: 5**	5.9 (6)	6.1 (7)	8.1 (3)	6.9 (7)	25.5 (1)	26.8 (1)
**Pep: 6**	1.9	0.1	2.3	2.9	3.0	2.7
**Pep: 7**	4.2 (8)	4.3 (10)	5.1 (6)	9.3 (4)	7.4 (7)	9.3 (5)
**Pep: 8**	2.7	0.0	1.8	3.0	17.2 (2)	17.1 (3)
**Pep: 9**	4.0 (10)	0.0	0.2	2.1	0.0	0.0

Supplementary Table S7: Average number of hydrogen bonds formed between the TCR to each pHLA residue for our simulations of wildtype and enhanced affinity DMF5, MEL5 and Tax A6. For each TCR-pHLA complex the 10 pHLA residues with the greatest number of contacts are coloured red, with their rank provided in brackets.

	TCR-pHLA Complex					
**Residue**	**DMF5**	**DMF5_ YW**	**MEL5**	**MEL5_ α24β17**	**A6**	**A6_ c134**
**HLA: 19**	0.0	0.0	0.1	0.0	0.0	0.0
**HLA: 43**	0.0	0.0	0.2	0.0	0.0	0.0
**HLA: 55**	1.0 (5)	1.1 (5)	0.0	0.0	0.9 (9)	1.1 (8)
**HLA: 58**	0.3	1.0 (6)	0.1	0.8	0.5	0.5
**HLA: 59**	0.0	0.2	0.0	0.0	0.0	0.0
**HLA: 61**	0.1	0.3	0.1	0.3	0.0	0.0
**HLA: 62**	0.0	0.1	0.0	0.0	0.0	0.0
**HLA: 65**	1.5 (4)	1.6 (3)	2.1 (1)	2.0 (2)	2.5 (1)	2.6 (1)
**HLA: 66**	0.6 (10)	0.9 (9)	0.0	0.7	1.0 (7)	1.3 (5)
**HLA: 68**	0.1	0.0	0.5 (8)	1.0 (9)	0.0	0.0
**HLA: 72**	2.0 (1)	2.4 (1)	1.5 (2)	2.7 (1)	0.2	0.2
**HLA: 73**	0.1	0.2	0.6 (7)	0.0	0.0	0.0
**HLA: 75**	0.5	0.5	0.2	0.0	0.0	0.0
**HLA: 145**	0.0	0.1	0.0	0.0	0.0	0.0
**HLA: 146**	0.1	0.2	0.0	0.0	0.0	0.0
**HLA: 149**	0.0	0.2	0.0	0.0	0.4	0.2
**HLA: 150**	0.0	0.1	0.0	0.1	0.4	0.8
**HLA: 151**	0.0	0.1	0.0	0.2	0.4	0.8
**HLA: 154**	0.1	0.0	0.3	1.3 (6)	0.3	0.2
**HLA: 155**	0.7 (8)	0.9 (7)	0.5 (9)	0.8 (10)	1.0 (6)	1.5 (4)
**HLA: 157**	0.0	0.0	0.0	1.1 (8)	0.1	0.1
**HLA: 158**	0.2	0.2	0.0	0.2	0.3	0.2
**HLA: 161**	0.1	0.1	0.0	1.1 (7)	0.2	0.3
**HLA: 162**	0.0	0.0	0.0	0.1	0.0	0.0
**HLA: 163**	0.5	0.5	0.4 (10)	0.6	0.9 (8)	0.9 (10)
**HLA: 166**	0.4	0.8	0.9	0.1	1.4 (4)	1.1 (9)
**HLA: 167**	0.4	0.3	0.0	0.5	0.5	0.5
**HLA: 170**	0.1	0.0	0.4	0.0	0.1	0.1
**Pep: 1**	1.7 (3)	1.8 (2)	0.6 (6)	1.5 (4)	0.0	0.0
**Pep: 2**	0.7 (9)	0.5	0.2	0.4	0.7	0.5
**Pep: 3**	0.0	0.1	0.0	0.0	0.0	0.0
**Pep: 4**	1.8 (2)	1.3 (4)	1.2 (3)	1.4 (5)	2.2 (3)	1.9 (3)
**Pep: 5**	0.2	0.1	0.1	0.0	1.1 (5)	1.1 (7)
**Pep: 6**	0.1	0.0	0.2	0.1	0.9 (10)	1.2 (6)
**Pep: 7**	0.7 (7)	0.8 (10)	0.9 (5)	1.6 (3)	2.3 (2)	2.1 (2)
**Pep: 9**	0.8 (6)	0.9 (8)	0.4	0.2	0.0	0.0

Supplementary Methods

Cloning, expression and refolding of proteins. The TCR α and TCR β-chains, as well as the HLA class I α-chains (tagged and not tagged with a biotinylation sequence) and β2m sequences, were cloned into the pGMT7 expression vector under the control of the T7 promoter using BamH1 and EcoR1 restriction sites, as described previously[1–3]. All sequences were confirmed by automated DNA sequencing. The TCR α and TCR β-chains, the HLA-A*02:01 α chains and β2m were expressed separately, without post-translational modification, as insoluble inclusion bodies in competent Rosetta DE3 *Escherichia coli* cells using 1 mM IPTG, as described previously[1–3]. For a 1 L TCR refold, 30 mg TCR α-chain was incubated at 37 °C for 30 min with 10 mM DTT and added to cold refold buffer (50 mM TRIS pH 8.1, 2 mM EDTA, 2.5 M urea, 6 mM cysteamine hydrochloride and 4 mM cystamine). After 30 min, 30 mg TCR β-chain, also incubated at 37°C for 30 min with 10 mM DTT, was added. For a 1 L pHLA-I refold, 30 mg HLA-A*02:01 α-chain was mixed with 30 mg β2m and 4 mg of peptide at 37 °C for 30 min with 10 mM DTT. This mixture was then added to cold refold buffer (50 mM TRIS pH 8.1, 2 mM EDTA, 400 mM L-arginine, 6 mM cysteamine hydrochloride, and 4 mM cystamine). TCR and pHLAI refolds were mixed at 4 °C for >1 hr and dialyzed against 10mM TRIS pH 8.1 until the conductivity of the refolds was <2 mS/cm. All the refolds were then filtered, ready for purification. Refolded proteins were purified, initially by ion exchange using a Poros50HQTM column (Thermo Fisher Scientific Inc, MA, USA.), and finally gel filtered into crystallization buffer (10 mM TRIS pH 8.1 and 10 mM NaCl) or BIAcore buffer (10 mM HEPES pH 7.4, 150 mM NaCl, 3 mM EDTA and 0.005% (v/v) surfactant P20) using a Superdex200HRTM column (GE Healthcare, Buckinghamshire, U.K.). Protein quality, either under non-reducing or reducing conditions, was analyzed by Coomassie-stained SDS-PAGE.

Molecular Dynamics Simulations. Starting structures for all simulations were obtained from previously solved X-ray crystal structures from multiple studies (**Table 1 and Supplementary Tables S2&S3**), with any missing residues added using Modeller[4]. Molecular dynamics (MD) simulations of apo-TCR structures were initiated from the TCR-pHLA bound structure. MolProbity[5] was used to determine the optimum tautomerisation states for all histidine residues and make any required Asn/Gln side chain flips under the criteria of optimising the hydrogen bonding network. Protonation states of all titratable residues were assigned using PropKa 3.0[6] for pH 7. All structures were then solvated in an octahedral water box (retaining any crystal waters), ensuring all protein atoms were at least 10 Å away from the box boundary, with Na^+ or Cl^- counter ions added, as necessary, to ensure an overall neutral charge. MD simulations were performed using GPU accelerated Amber16[7], with the ff14SB force field[8] and TIP3P water model used to describe protein and water molecules, respectively. All systems investigated were equilibrated to 300 K and 1 atm in the NPT ensemble (see section “MD Equilibration Procedure” below). Subsequently, production MD simulations were performed for 100 ns each with 10 replicas performed per TCR-pHLA or apo-TCR structure. Production MD simulations were run using a 2 fs time step with the SHAKE algorithm applied to any bond containing a hydrogen atom. An 8 Å direct space non-bonded cut-off was applied with long range electrostatics evaluated using the particle mesh Ewald algorithm[9]. Temperature was regulated using Langevin temperature control (collision frequency of 1 ps^{-1}), whilst pressure was controlled with a Berendsen barostat (setting the pressure relaxation time to 1 ps).

MD Equilibration Procedure. The following procedure was used to prepare all systems simulated for production MD simulations at 300 K and 1 atm. Furthermore, the equilibration protocol used is identical for both our "long" timescale (5 x 100 ns) and "short" time scale (25 x 4 ns) MD simulations. All dynamics steps applied the SHAKE algorithm to constrain all bonds containing hydrogen. Replicas simulations were initiated from the second heating step of the following protocol (with each replica therefore assigned different random velocity vectors at this stage). First hydrogens atoms and solvent molecules were energy minimised (using 500 steps of steepest descent followed by 500 steps of conjugate gradient minimisation). To prevent the movement of non-hydrogen and non-solvent atoms during the minimisation, 10 kcal mol^{-1} $Å^{-1}$ positional restraints were used to keep all heavy atoms fixed. Then the solvent was heated rapidly from 50 K to 300 K (NVT ensemble, 1 fs timestep) over the course of 200 ps, with the previously described restraints still maintained. The positional restraints were then replaced with 5 kcal mol^{-1} $Å^{-1}$ positional restraint on only the Cα carbon atoms and subjected to another round of energy minimisation (500 steps of steepest descent followed by 500 steps of conjugate gradient). Retaining these positional restraints, the system was heated from 25 K to 300 K over the course of 50 ps (NVT ensemble, 1 fs time step). Simulations were then performed in the NPT ensemble (1 atm, 300 K, 2 fs time step) by first gradually reducing the 5 kcal mol^{-1} $Å^{-1}$ Cα carbon restraints over the course of 50 ps. This was done by reducing the restraint weight by 1 kcal mol^{-1} $Å^{-1}$ every 10 ps. The end structure from this run was then used as the starting structure for production MD simulations. Simulations performed in the NVT ensemble used Langevin temperature control (with a collision frequency of 1 ps^{-1}) and used a simulation timestep of 1 fs. Simulations performed in the NPT ensemble again used Langevin temperature control (collision frequency of 1 ps^{-1}) and a Berendsen barostat (1 ps pressure relaxation time).

MD Simulation Analysis. Trajectory analysis was performed with CPPTRAJ[10], using frames collected every 10 ps for analysis of MD simulations. Hydrogen bonds (HBs), including water bridged HBs between atoms of the TCR and pHLA were defined as formed if the donor acceptor distance was within 3.5 Å and the donor hydrogen acceptor angle was 180 ± 45°. If two heavy (non-hydrogen) atoms were within 4 Å of one another, a van der Waals (vdWs) contact was considered to be formed between the two atoms. RMSF calculations were performed on the Cα atoms of the relevant residues with RMS fitting performed to a consistent set of TCR residues in the variable domains that are not highly flexible (see section "RMS Fitting Procedure" below). RMSFs, HBs and vdWs contacts were calculated by discarding the first 10 ns of simulation time (meaning 10 replicas of 10-100 ns used) to allow for structure equilibration. Buried solvent accessible surface area (BSASA) was determined using with the LCPO algorithm[11], available with the "molsurf" command within CPPTRAJ[10].

RMS Fitting Procedure. To ensure fair comparison between the TCR structures, RMS fitting (for RMSF calculations) was performed using the same set of residues in the TCR variable region. Residues excluded from the RMS fitting procedure were the first five N-terminal residues and all CDR loop residues (due to their high mobility, which would therefore provide a poor fit). Residues used for RMS fitting from Chain A were therefore: 6-22,33-46,55-65,73-94,103-113. Residues used for RMS fitting from Chain B were: 6-21,30-47,54-65,73-91,102-111. RMS fitting of MD simulation snapshots was first performed to the crystal structure, with this RMS fitted trajectory used to create an average structure. Following this, all snapshots were then re-fitted to the average structure for the subsequent RMSF calculation.

MMGBSA Methodology. The molecular mechanics generalized Born surface area (MMGBSA) method is a binding free energy calculation method which has been widely used to predict relative binding free energies[12,13]. The approach uses a combination of MD simulations (for sampling to obtain many snapshots) and empirical calculations (on the obtained snapshots) to predict ΔG_{bind}. In the MMGBSA approach, the different contributions to affinity are calculated individually and summed together to obtain ΔG_{bind} (see Equation 1):

Equation S1: $$\Delta G_{bind} = \Delta E_{el} + \Delta E_{vdw} + \Delta G_{pol} + \Delta G_{npol} - T\Delta S \qquad (1)$$

Where ΔE_{el} and ΔE_{vdw} are obtained directly from the molecular mechanics force field terms and describe the gas phase interaction energy. The polar and non-polar contributions to the solvation free energy are described by ΔG_{pol} and ΔG_{npol}, respectively. ΔG_{pol} is calculated by solving the GB equation, whilst ΔG_{npol} is obtained from a function that assumes a linear relationship between the solvent accessible surface area and ΔG_{npol}. Finally, $T\Delta S$ describes the change in entropy of the solute upon binding, most often calculated through normal mode analysis (NMA). For large systems like TCR-pHLA, NMA is computationally expensive and also tends to produce large errors that do not improve the accuracy of the calculation[14]. Furthermore, as it is not possible to decompose the results from NMA to a per-residue level, we did not perform NMA for our MMGBSA calculations.

MMGBSA Calculations. MMGBSA calculations were performed using MMPBSA.py.MPI[15], using 25 independent (random velocity vectors assigned upon heating) 4 ns long MD simulations (separate to the above described 100 ns long simulations). These simulations were run under the same conditions as the aforementioned longer timescale simulations. From each run, 300 equally spaced snapshots were taken from the last 3 ns of each MD simulation for MMGBSA calculations, giving a total of 7500 frames per complex. MMGBSA calculations used the GB-Neck2 (i.e. igb = 8) solvation model and an implicit salt concentration of 150 mM. The obtained results were decomposed into their per-residue contributions to the total free energy, with the values obtained used to calculate the differences between the WT and eaTCRs.

Supplementary References

1. Boulter, JM, Glick, M, Todorov, PT, Baston, E, Sami, M, Rizkallah, P, *et al.* (2003). Stable, soluble T-cell receptor molecules for crystallization and therapeutics. *Protein Eng.* **16**: 707–11.
2. Garboczi, DN, Utz, U, Ghosh, P, Seth, A, Kim, J, VanTienhoven, EA, *et al.* (1996). Assembly, specific binding, and crystallization of a human TCR-alphabeta with an antigenic Tax peptide from human T lymphotropic virus type 1 and the class I MHC molecule HLA-A2. *J. Immunol.* **157**: 5403–10.
3. Garboczi, DN, Hung, DT and Wiley, DC (1992). HLA-A2-peptide complexes: refolding and crystallization of molecules expressed in Escherichia coli and complexed with single antigenic peptides. *Proc. Natl. Acad. Sci. U. S. A.* **89**: 3429–3433.
4. Webb, B and Sali, A (2014). Comparative Protein Structure Modeling Using MODELLER. *Curr. Protoc. Bioinforma.* **47**: 5.6.1-5.6.32.
5. Chen, VB, Arendall, WB, Headd, JJ, Keedy, DA, Immormino, RM, Kapral, GJ, *et al.* (2010). *MolProbity* : all-atom structure validation for macromolecular crystallography. *Acta Crystallogr. Sect. D Biol. Crystallogr.* **66**: 12–21.
6. Søndergaard, CR, Olsson, MHM, Rostkowski, M and Jensen, JH (2011). Improved Treatment of Ligands and Coupling Effects in Empirical Calculation and Rationalization of pKa Values. *J. Chem. Theory Comput.* **7**: 2284–95.
7. D.A. Case, D.S. Cerutti, T.E. Cheatham, III, T.A. Darden, R.E. Duke, T.J. Giese, H. Gohlke, A.W. Goetz, D. Greene, N. Homeyer, S. Izadi, A. Kovalenko, T.S. Lee, S. LeGrand, P. Li, C. Lin, J. Liu, T. Luchko, R. Luo, D. Mermelstein, K.M. Merz, G. Monard, H., DMY and PAK (2017). AMBER 2016. *Univ. Calafornia San Fr.*
8. Maier, JA, Martinez, C, Kasavajhala, K, Wickstrom, L, Hauser, KE and Simmerling, C (2015). ff14SB: Improving the Accuracy of Protein Side Chain and Backbone Parameters from ff99SB. *J. Chem. Theory Comput.* **11**: 3696–713.
9. Darden, T, York, D and Pedersen, L (1993). Particle mesh Ewald: An *N* ·log(*N*) method for Ewald sums in large systems. *J. Chem. Phys.* **98**: 10089–10092.
10. Roe, DR and Cheatham, TE (2013). PTRAJ and CPPTRAJ: Software for Processing and Analysis of Molecular Dynamics Trajectory Data. *J. Chem. Theory Comput.* **9**: 3084–3095.
11. Weiser, J, Shenkin, PS and Still, WC (1999). Approximate atomic surfaces from linear combinations of pairwise overlaps (LCPO). *J. Comput. Chem.* **20**: 217–230.
12. Genheden, S and Ryde, U (2015). The MM/PBSA and MM/GBSA methods to estimate ligand-binding affinities. *Expert Opin. Drug Discov.* **10**: 449–461.
13. Chen, F, Liu, H, Sun, H, Pan, P, Li, Y, Li, D, *et al.* (2016). Assessing the performance of the MM/PBSA and MM/GBSA methods. 6. Capability to predict protein–protein binding free energies and re-rank binding poses generated by protein–protein docking. *Phys. Chem. Chem. Phys.* **18**: 22129–22139.
14. Sun, H, Duan, L, Chen, F, Liu, H, Wang, Z, Pan, P, *et al.* (2018). Assessing the performance of MM/PBSA and MM/GBSA methods. 7. Entropy effects on the performance of end-point binding free energy calculation approaches. *Phys. Chem. Chem. Phys.*doi:10.1039/C7CP07623A.
15. Miller, BR, McGee, TD, Swails, JM, Homeyer, N, Gohlke, H and Roitberg, AE (2012). *MMPBSA.py* : An Efficient Program for End-State Free Energy Calculations. *J. Chem. Theory Comput.* **8**: 3314–3321.
