# Supplementary material for: Molecular Rules Underpinning Enhanced Affinity Binding of Human T Cell Receptors Engineered for Immunotherapy
Source: Mol Ther Oncolytics. 2020 Jul 31;18:443–56. doi: 10.1016/j.omto.2020.07.008 (PMC7452143; doi:10.1016/j.omto.2020.07.008)
Supplement: Document S2. Article plus Supplemental Information [file mmc2.pdf]

Molecular Therapy
Oncolytics
Original Article

Molecular Rules Underpinning Enhanced Affinity Binding of Human T Cell Receptors Engineered for Immunotherapy

Rory M. Crean,[1,2] Bruce J. MacLachlan,[3,8] Florian Madura,[3] Thomas Whalley,[3] Pierre J. Rizkallah,[3] Christopher J. Holland,[4] Catriona McMurran,[4] Stephen Harper,[4] Andrew Godkin,[3] Andrew K. Sewell,[3] Christopher R. Pudney,[1,5] Marc W. van der Kamp,[6,7] and David K. Cole[3,4,7]

[1]Department of Biology and Biochemistry, University of Bath, Bath, BA2 7AY, UK; [2]Doctoral Training Centre in Sustainable Chemical Technologies, University of Bath, Bath, BA2 7AY, UK; [3]Division of Infection & Immunity, Cardiff University, Cardiff, CF14 4XN, UK; [4]Immunocore, Ltd., Abingdon, OX14 4RY, UK; [5]Centre for Therapeutic Innovation, University of Bath, Bath, BA2 7AY, UK; [6]School of Biochemistry, University of Bristol, Biomedical Sciences Building, University Walk, Bristol, BS8 1TD, UK

Immuno-oncology approaches that utilize T cell receptors (TCRs) are becoming highly attractive because of their potential to target virtually all cellular proteins, including cancer-specific epitopes, via the recognition of peptide-human leukocyte antigen (pHLA) complexes presented at the cell surface. However, because natural TCRs generally recognize cancer-derived pHLAs with very weak affinities, efforts have been made to enhance their binding strength, in some cases by several million-fold. In this study, we investigated the mechanisms underpinning human TCR affinity enhancement by comparing the crystal structures of engineered enhanced affinity TCRs with those of their wild-type progenitors. Additionally, we performed molecular dynamics simulations to better understand the energetic mechanisms driving the affinity enhancements. These data demonstrate that supra-physiological binding affinities can be achieved without altering native TCR-pHLA binding modes via relatively subtle modifications to the interface contacts, often driven through the addition of buried hydrophobic residues. Individual energetic components of the TCR-pHLA interaction governing affinity enhancements were distinct and highly variable for each TCR, often resulting from additive, or knock-on, effects beyond the mutated residues. This comprehensive analysis of affinity-enhanced TCRs has important implications for the future rational design of engineered TCRs as efficacious and safe drugs for cancer treatment.

INTRODUCTION

Recent advances in immuno-oncology (IO) have revolutionized the treatment of some cancers by harnessing and redirecting T cells against tumors. These successes are driving new areas of IO development, including exploiting T cell receptor (TCR) recognition of short antigenic peptide fragments presented at the cell surface by peptide-human leukocyte antigens (pHLAs). These peptide fragments represent virtually all cellular proteins, allowing TCRs to access a much larger pool of potential therapeutic targets than monoclonal antibodies (mAbs), which primarily bind to extracellular antigens.[1] This advantage has encouraged the development of soluble engineered TCRs as therapeutics for viral and cancerous diseases.[2–4] Soluble TCRs have been designed as bispecific T cell engagers by coupling with a T cell-activating antibody domain.[5] This approach of utilizing a soluble bispecific TCR to target cancer has been shown to induce tumor regression in mice,[6] and clinical trials are currently under way for multiple diseases.

To achieve high sensitivity as a soluble receptor, mAbs undergo a natural process of somatic hypermutation to generate affinities for their target antigens in the nanomolar to picomolar range. However, naturally occurring TCRs are selected in the thymus to bind pHLAs with relatively weak affinities (~micromolar) and short half-lives (seconds).[7,8] TCRs that recognize cancer-derived pHLAs are at the weaker end of the TCR-affinity scale,[9] reflecting a further disadvantage in their use to drive functional T cell responses against cancer. Although it is not fully understood why TCRs are selected with these binding characteristics, published evidence has demonstrated an optimal TCR affinity window for T cell triggering,[10–12] and that peptide binding must be degenerate (i.e., TCRs must be able to functionally bind to many thousands of different peptides) in order to provide immune coverage against all possible foreign antigens, while remaining tolerant to self-antigens.[13–16] Finally, weak TCR affinity may enable T cells to rapidly disengage from target cells to allow them to effectively penetrate tissues. This may contribute to the observation

Received 15 July 2020; accepted 27 July 2020;
https://doi.org/10.1016/j.omto.2020.07.008.

[7]These authors contributed equally to this work.

[8]Present address: Monash Biomedicine Discovery Institute, 19 Innovation Walk, Clayton, VIC 3800, Australia

Correspondence: Marc W. van der Kamp, School of Biochemistry, University of Bristol, Biomedical Sciences Building, University Walk, Bristol, BS8 1TD, UK.
E-mail: marc.vanderkamp@bristol.ac.uk
Correspondence: David K. Cole, Immunocore, Ltd., Abingdon, OX14 4RY, UK.
E-mail: david.cole@immunocore.com

This is an open access article under the CC BY license (http://creativecommons.org/licenses/by/4.0/).

Table 1. Structural Analyses of all TCR-pHLAs Complexes under Investigation

TCR-pHLA	TCR	PDB	K_D (nM)	On-Rate ($M^{-1}s^{-1}$)	Off-Rate (s^{-1})	Crossing Angle (°)	S_C
1G4-HLA-A*0201-SLL	wild-type	2BNR[59]	13,300	12,000	0.049	69.4	0.71
	1G4_c5c1	2PYE[28]	81.6	17,800	0.0015	65.4	0.77
	1G4_c49c50	2F53[3]	1	180,000	0.00024	65.9	0.77
	1G4_c58c61	2P5E[28]	0.048	570,000	0.00003	66.3	0.76
	1G4_c58c62	2P5W[28]	nm	nm	0.00003	65.7	0.78
DMF5-HLA-A*0201-ELA	wild-type	3QDG[60]	29,000	nm	nm	33.3	0.65
	DMF5_YW	4L3E[23]	24	nm	nm	31.7	0.64
MEL5-HLA-A*0201-ELA	wild-type	3HG1[47]	18,000	nm	nm	47.6	0.64
	MEL5_α24β17	4JFF[34]	0.6	179,000	0.0001	42.2	0.66
MEL5-HLA-A*0201-EAA	wild-type	4QOK[61]	8,400	nm	nm	46.9	0.64
	MEL5_α24β17	6TMO	0.75	280,000	0.00021	42.6	0.67
MEL5-HLA-A*0201-AAG	wild-type	6EQA[62]	14,200	nm	nm	48.0	0.57
	MEL5_α24β17	6EQB[62]	26.2	74,000	0.0019	42.3	0.71
A6-HLA-A*0201-LLF	wild-type	1AO7[63]	3,200	23,000	0.074	33.5	0.63
	A6_c134	4FTV[27]	4	45,000	0.00018	32.9	0.74
ILA1-HLA-A*0201-ILA	wild-type	5MEN[16]	34,000	3,490	0.13	39.8	0.64
	ILA1_α1β1	4MNQ[29]	2	80,000	0.00016	42.1	0.57

Surface complementarity (S_C) was determined using ePISA. K_D, affinity dissociation constant; nm, not measured

that stronger affinity chimeric antigen receptor (CAR)-T cells have a limited ability to penetrate solid tumors.[17] This thymically selected balance between functionality and self-tolerance is also likely reflected in the relatively conserved binding mode that has been observed for the majority of TCR-pHLA complexes, which places the TCR diagonally over the center of the pHLA peptide-binding groove, enabling a broad contact interface with both the peptide and HLA surface.

The natural weak affinity of TCRs imposes limitations on their use as soluble therapeutics. Thus, in parallel with protein engineering strategies that have been used to generate therapeutic antibodies[18] and other therapeutic protein molecules,[19] a number of approaches focused on introducing affinity-enhancing mutations within the six complementarity determining region (CDR) loops that comprise the TCR binding site,[3,20–25] or residues within the variable domain interface, have been used.[26] These mutations are designed to improve the binding characteristics of the TCR-pHLA interaction, with the intention of maintaining native characteristics, such as self-tolerance, that are selected naturally during thymic selection.

In this study, we explored the fundamental mechanisms that underpin the interactions between engineered affinity-enhanced human TCRs (aeTCRs) with pHLAs. With many possible mechanisms available for affinity enhancement (such as improved electrostatics, burial of hydrophobic residues, expulsion of unfavorable water molecules, and a reduction in the entropic cost of solute binding by rigidification of the protein), the identification of principles by which TCRs can be affinity enhanced, yet retain their native binding characteristics, would be beneficial for the rational design of antigen-selective therapeutic TCRs. We compared the structures of wild-type (WT) and multiple aeTCRs specific for for distinct cancer-derived pHLAs and one virally-derived pHLA. In addition, we performed molecular dynamics (MD) simulations and binding free energy calculations to determine the energetic mechanisms driving affinity enhancement. These data reveal new insights into the flexibility of the native TCR-pHLA complex and demonstrate that this interaction is globally compatible with large affinity enhancements without major reconstruction of the binding interface. These findings have important implications for our understanding of the basic principles that govern thymic selection of "weak"-affinity natural TCRs, and for the development of *in silico* rational design approaches for therapeutic aeTCRs.

RESULTS

aeTCRs Show Preservation of the WT Binding Mode

We analyzed the crystal structures of all published structures of TCR-pHLA complexes where there exists a WT and an enhanced affinity version of the same TCR (Table 1), and further solved the structure of an enhanced affinity version of the MEL5 TCR (MEL5_α24β17 aeTCR) in complex with human leukocyte antigen (HLA)-A*0201-EAAGIGILTV (A2-EAA) at 2.1 Å resolution (Table S1). Each of the aeTCRs were previously generated through directed evolution approaches by positive selection on capacity to bind pHLA antigen, except for DMF5_YW, which was affinity enhanced *in silico*.

We directly compared their overall binding geometry (crossing angle, shape complementarity [S_C], positions of the CDR loops) and total contacts (Figure 1). Overall, and in line with previously published findings,[16,27–29] all aeTCRs bound their cognate pHLA

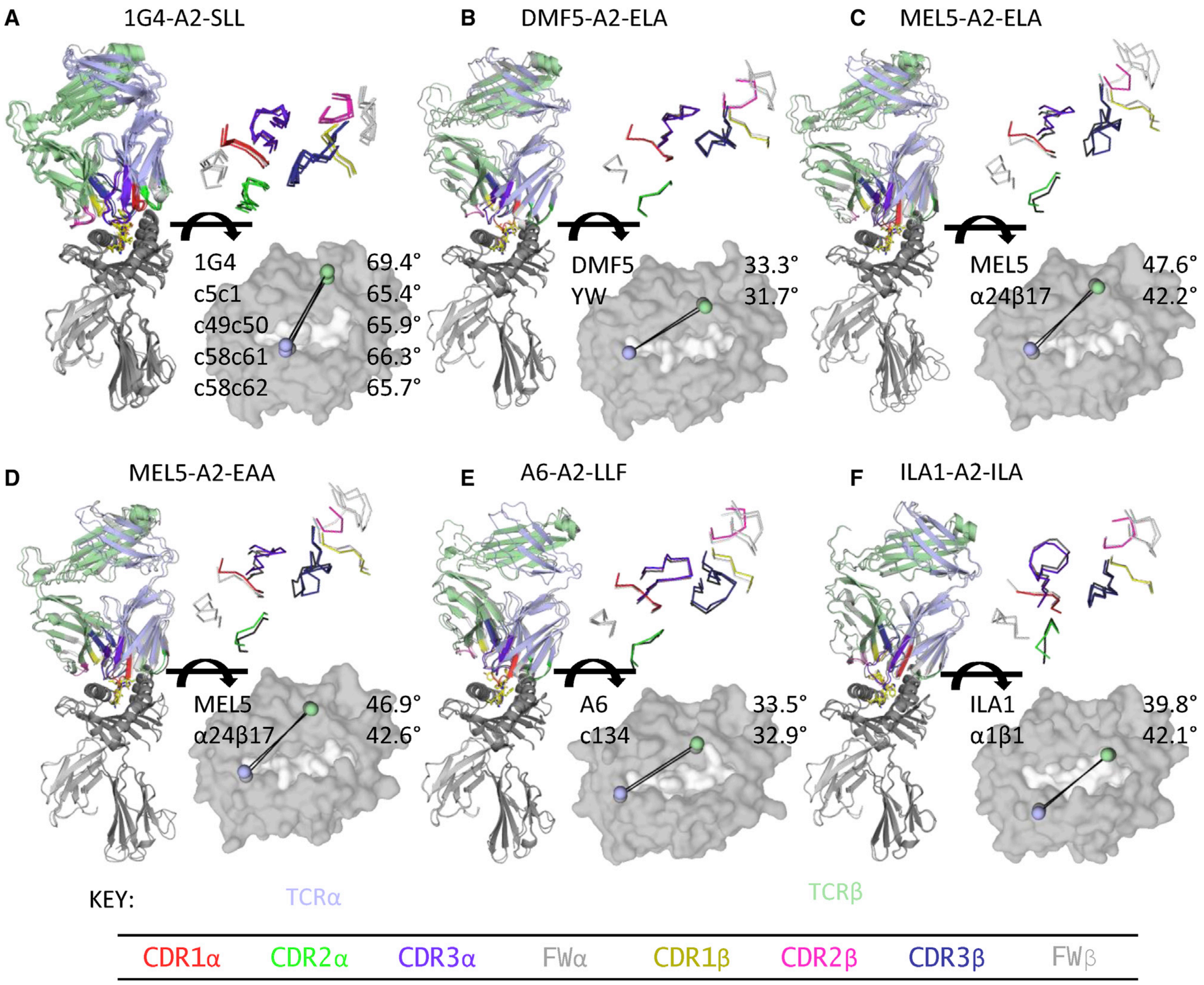

Figure 1. Structural Comparison of Overall Binding Modes of Affinity Enhanced and Wild-Type TCR-pHLA Complexes Shows Virtually Identical Conformations

(A–F) Left: structural overlay of wild-type (WT) TCR-pHLA complexes versus affinity-enhanced (ae)TCR-pHLA complexes. TCR and HLA are shown as cartoon and peptide is shown as stick representation. Right top: overlay of CDR loop positions of WT and aeTCRs. Backbone locations of each CDR loop shown as line representation. In each panel, the WT TCR complex structure is in grayscale and the aeTCR complex structure is colored by chain: TCRα, blue; TCRβ, green; HLA, dark gray; β2m, light gray. Right bottom: TCR crossing angle of WT TCR-pHLA complexes versus aeTCR-pHLA complexes. pHLA is shown as surface representation (gray). TCRα (blue) and TCRβ (green) centroid locations of aeTCR structure are shown as spheres. (A) 1G4 TCR and multiple aeTCR variants in complex with HLA-A*0201-SLLMWITQC. (B) DMF5 TCR and aeTCR variant DMF5_YW in complex with HLA-A*0201-ELAGIGILTV. (C) MEL5 TCR and aeTCR variant MEL5_α24β17 in complex with HLA-A*0201-ELAGIGILTV. (D) MEL5 TCR and aeTCR variant MEL5_α24β17 in complex with HLA-A*0201-EAAGIGILTV. (E) A6 TCR and aeTCR variant A6_c134 in complex with HLA-A*0201-LLFGYPVYV. (F) ILA1 TCR and aeTCR variant ILA1_α1β1 in complex with HLA-A*0201-ILAKFLHWL.

with virtually identical crossing angles and CDR loop positions compared to their WT progenitor TCRs (Figures 1A–1F). We also compared the binding footprint (in terms of the TCR position from the center of the pHLA, toward either the N or C terminus of the peptide, or the α1 or α2 helices of the HLA) of the aeTCRs to all published TCR-pHLA complexes (Figure S1). The aeTCRs were all within the normal distribution of known TCR-pHLA complexes for both parameters. Most of the aeTCRs demonstrated slightly increased S_C, in line with their increases in affinity, but this metric was not predictive of overall affinity gains (Table 1). For example, an enhanced affinity version of the 1G4 TCR (1G4_c58c61), which underwent the largest affinity gain compared to its WT TCR progenitor (~300,000-fold), demonstrated one of the smallest gains in favorable S_C compared to other aeTCRs.

aeTCRs Generally Form Additional Contacts with HLA

Assessment of the number of additional contacts formed between the aeTCRs and the pHLA from the structural analysis

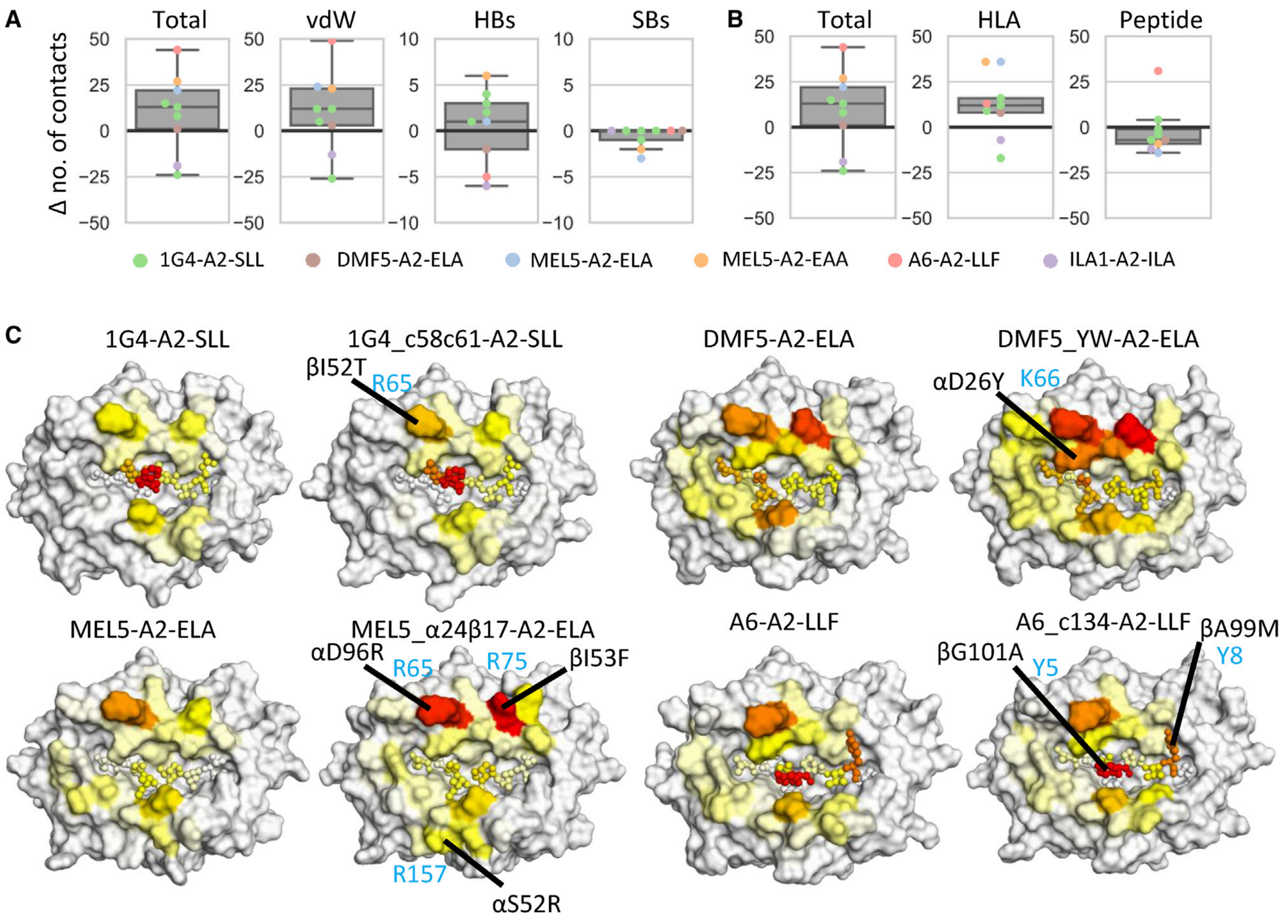

Figure 2. Differences in the Average Number of Contacts Formed during pHLA Engagement of WT TCRs versus aeTCRs

(A) Difference (Δ) in the number of contacts between WT TCRs and aeTCRs segregated by contact type analyzed using crystal structures. Boxplots represent median (middle line), interquartile ranges Q1 (box lower) and Q3 (box upper), Q1-1.5*IQR (lower whisker), and Q3+1.5*IQR (upper whisker). Individual scatter points of each TCR are overlaid and colored by the TCR-pHLA system-indicated inset. vdW, van der Waals (≤4.0 Å); HBs, hydrogen bonds (≤3.4 Å); SBs, salt bridges (≤3.4 Å). (B) Difference in the number of contacts between WT and aeTCRs from crystal structures segregated by contacts to HLA or peptide atoms or both (total). (C) Surface plots of the pHLA (peptide atoms shown as spheres) with each structure color mapped according the average number of vdWs contacts formed between the given residue and the TCR during the course of MD simulations. Color mapping was performed from white (no contacts) through yellow and orange to red (highest number of contacts observed for each of the pairs of TCR-pHLAs studied). All pHLA structures are shown in the same orientation, such that the peptide N terminus is left, and the C terminus is right. TCR residues contributing substantially to new contacts are labeled in black and indicated with black lines. Corresponding peptide or HLA residues are labeled in light blue. For brevity, only one 1G4 aeTCR is shown; all others are shown in Figure S2. Note that (A) and (B) were generated from crystal structure analysis, while (C) was generated from MD simulation data.

demonstrated a modest increase in the total number of van der Waals (vdWs) contacts and hydrogen bonds (HBs) for most complexes (Figures 2A and 2B). Although most aeTCRs demonstrated an increase in the total number of vdWs interactions, the 1G4 aeTCRs had virtually the same, or in one case lower, numbers of vdWs contacts compared to the WT 1G4 TCR (Figure 2A). We observed a similar average number of HBs, and a lower average number of salt bridges (SBs) for the aeTCRs. aeTCRs exhibited a trend toward increases in new contacts to HLA residues as opposed to the peptide residues (Figure 2B); except for the A6_c134 aeTCR, which was the only TCR to make new contacts with peptide residues. In most cases, this was driven by mutations that increased the size and hydrophobicity of the TCR paratope.

To further unpick the contribution of each introduced mutation to the binding interface, we turned to MD simulations (Tables S2 and S3), performing 10 replicas of 100 ns each in both their apo and pHLA-bound forms (totaling 22 μs of MD simulation). The use of many independent replicas (such as the 10 performed here) is important for obtaining reliable and reproducible results.[30] This enabled insight into how the number of contacts between the TCR and pHLA changed over time (Figure 2C; Figures S1C–S1E and S2; Tables S4–S7), rather than relying on static images generated from crystal

structures alone. Overall, our findings were consistent compared to the crystal structures (Figure 2A), with small increases in both the average number of HBs and vdWs contacts for the MEL5 and DMF5 aeTCRs, and no clear relationship between affinity and the number of contacts for the 1G4 aeTCRs. This is consistent with previous findings that there is poor correlation between number of contacts and affinity.[31] For the A6_c134 TCR, although our MD simulations suggested a smaller increase in the average number of contacts compared to the analysis of the crystal structure, increases were observed in the region of the HLA and peptide adjacent to the modified residues in the A6_c134 TCR CDR3β loop (Table S7).

To measure the extent to which contacts to individual pHLA residues were preserved upon affinity enhancement, we calculated the total average number of HBs and vdWs contacts formed between the TCR and each pHLA residue. Of the 10 pHLA residues most contacted by the 1G4 TCR (as defined by either HBs or vdWs contacts), at least 8 were preserved for all aeTCR variants (Tables S4 and S5). For both the DMF5 and A6 TCRs, the aeTCR variants preserved at least 9 of the top 10 WT TCR contacts, while for the MEL5 TCR, 7 of the top 10 WT contacts were preserved (Tables S6 and S7). These results are consistent with the aeTCRs preserving a native TCR-pHLA binding footprint, combined with increases in contacts to existing as well as new pHLA residues (Figure 2C).

We also applied our MD simulations to determine how the average buried solvent-accessible surface area (BSASA) differed for all TCR-pHLA complexes (Figure S1E). While the DMF5- and MEL5-derived aeTCRs showed an increase in the BSASA, the A6- and 1G4-derived aeTCRs showed no significant change.

Energetic Hotspots Are Largely Preserved during the Course of Affinity Enhancement

To further assess how mutations affected the affinity between the TCR and pHLA, we performed binding free energy calculations using the molecular mechanics generalized born surface area (MMGBSA) method.[32] This approach uses MD simulations to sample conformations of the complex, receptor, and ligand, and subjects these snapshots to an empirical calculation to estimate the binding free energy (ΔG). We note that this approach should not be relied on for absolute binding affinities, and instead should be used to predict relative binding affinities among similar systems, as we have done here.[33] Comparison of our calculated $\Delta\Delta G$ values with the experimentally derived results from SPR experiments[3,23,27–29,34] showed that the differences in affinity between the aeTCRs and their WT progenitor TCRs were identified correctly (Figure 3A).

Decomposing the calculated binding energies onto a per residue level can indicate which interactions are the main drivers for the increased binding affinity. We note that these per residue decomposition values do not directly relate to a possible experimental measurement and should be used in a more qualitative manner to identify key favorable/unfavorable residues/interactions across the binding interface. While our primary focus was on the binding differences between aeTCRs and their WT progenitor TCRs (i.e., $\Delta\Delta G$), we showed that the energetic hotspots across the TCR-pHLA interface were largely conserved upon affinity enhancements (Figure 3B; Figure S3), in line with the observation that the number and types of contacts were largely maintained (Figures 1 and 2).

Decomposition of the Calculated Binding Energies Reveal the Molecular Basis for Affinity Enhancement

For the examples described in this study, the mutated residues were located in one of the six CDR loops, or the two hypervariable 4 (HV4) loops, which comprise the TCR paratope. However, most of the residues selected during the aeTCR generation do not make direct antigen contacts. Thus, we hypothesized that these residues may exert their effects indirectly, instead optimizing the residues that make direct contact with the pHLA. We therefore calculated the changes in the per residue contributions to the TCR-pHLA binding affinity upon affinity enhancement (i.e., $\Delta\Delta G$, Figures 4 and 5) to further understand how each of these mutations modified TCR-pHLA affinities. This analysis indicated that the 1G4 aeTCR loop mutations had a largely additive effect on TCR-pHLA binding; i.e., the contribution of mutations in one loop was not affected by mutations in other loops (Figure 4A). For the 1G4 aeTCR α chains, the two different mutations introduced into the CDR2α loop (Figures 4B and 4C) both appeared to improve affinity via the same mechanism: large hydrophobic (and aromatic) groups were introduced in locations where they can be effectively buried at the interface (either S53W, or S52F and S53W). The G97D mutation in the CDR3α loop was predicted to be unfavorable for all three cases in which it occurred, likely due to the partial burial (i.e., desolvation) of a charged residue upon binding (Figure 4D). However, the G97D mutation resulted in the formation of a new internal hydrogen bond (HB) within the CDR3α loop to T99 (S99 in the WT 1G4 TCR), relative to the WT 1G4 TCR. This mutation might help to rigidify and preorganize the CDR loop, and be compensated for by the S96L and S99T mutations introduced alongside. Furthermore, the S96L and S99T mutations likely benefit from a more rigid/preorganized CDR loop, as this would allow for stronger and more persistent interactions with the pHLA.

Further analysis of the impact of CDR3α mutations suggested a beneficial knock-on effect for HLA residue R65 and CDR2β residue D54, which form a salt-bridge with one another, as all CDR3α mutated aeTCRs formed an increased average number of HBs between the TCR and R65 (Table S5). For the 1G4 aeTCR β chains, the substitution of the methyl side chain of A50 for a larger hydrophobic side chain (A50V or A50I) in CDR2β was primarily responsible for the increased binding affinity. Furthermore, the G49A mutation (seen only in 1G4_c58c61) further increased the favorability toward binding, which contrasted with the more polar G49S mutation in the 1G4_c49c50 and 1G4_c58c62 aeTCRs (Figures 4E and 4F). CDR3β loop mutations that increased affinity were largely mediated through indirect effects, i.e., by increasing the favorability toward binding of the CDR1β loop residue E28 (Figure 4G). This improvement in E28 (seen only in TCRs with CDR3β loop mutations) was likely the result of increased preorganization of E28 for binding through an

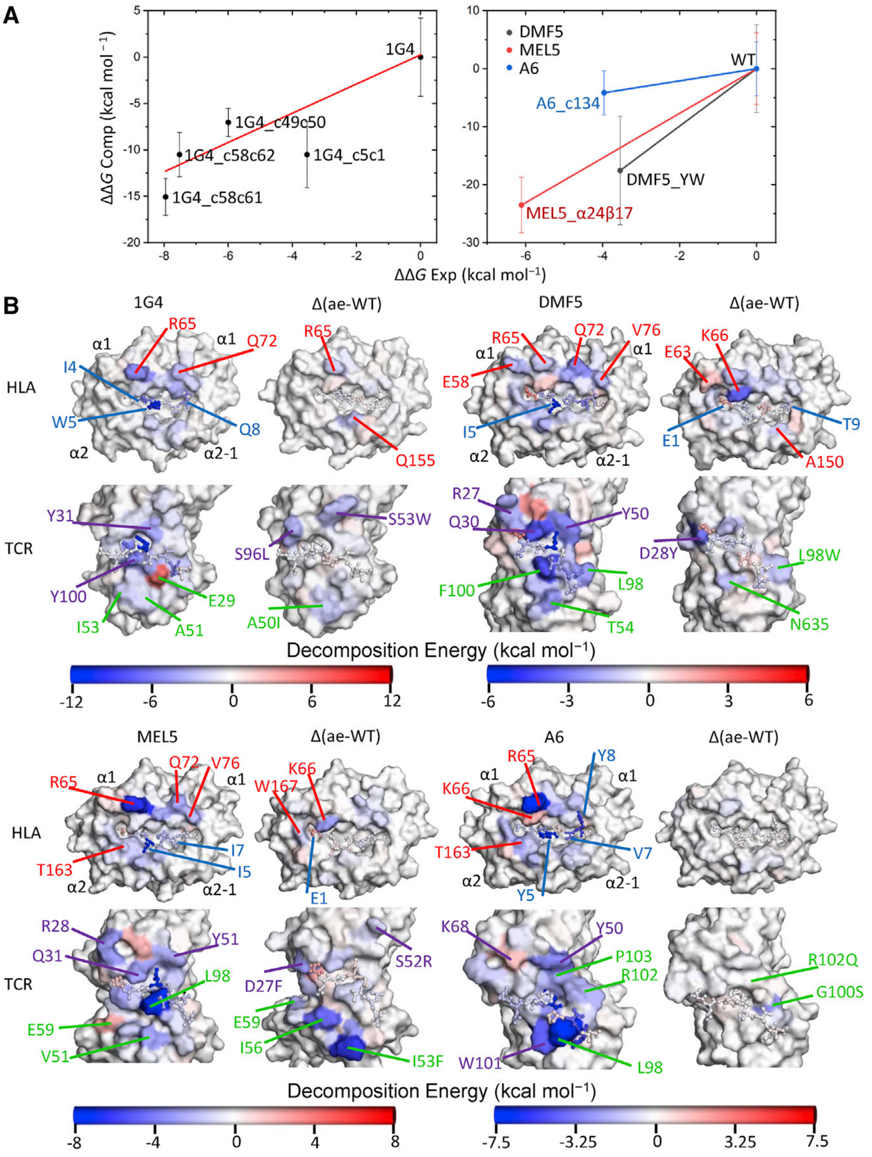

Figure 3. Changes in the Energetic Footprint between WT TCRs and aeTCRs

(A) Experimental versus computational ΔΔ*G* values obtained from our MMGBSA calculations for all TCR-pHLA systems studied. Error bars plotted are the standard deviation obtained from the 25 replicas performed per complex. (B) For all TCR-pHLA complexes, the HLA (top) and TCR (bottom) structures are plotted as surfaces with the peptide shown in both structures as ball and stick representations. All plots are color mapped according to the MMGBSA per residue decomposition results, going from blue (favorable binding) to white (neutral) to red (unfavorable binding) with the WT TCR-pHLA complex on the left, and the Δ (ae-WT) on the right for each system. Separate scaling is used for each of the four sets of TCRs studied as indicated by the color bars below each group (kcal mol^{-1}). All pHLA and TCR structures are shown in the same orientation, such that the peptide N terminus is left and the C terminus is right. Several mutations sites are indicated on the aeTCR variants (purple labels, CDRα mutations; green labels, CDRβ mutations). For brevity only one 1G4 aeTCR is shown. All others are shown in Figure S3.

increased strength HB between the side chain carboxyl group and the backbone of residue V/L94 (average HB occupancy in WT and 1G4_c49c50 simulations was between 0.50 and 0.56, compared to 1G4_c5c1, 1G4_c58c62, and 1G4_c58c61 where it was between 0.83 and 0.86).

All differences in sequence between the A6 TCR and A6_c134 aeTCR occurred on the CDR3β loop (A99M, G100S, G101A, and R102Q), and prior structural analysis suggested that the increased affinity was due to a greater number of contacts between the TCR and pHLA.[27] Our binding energy decomposition analysis indicated that mutations A99M and G100S were primarily responsible for the enhanced affinity (Figures 5A and 5B), and in line with the prior structural characterization, we observed an increase in the total average number of contacts made between the TCR and the pHLA residues that sit below the CDR3β loop residues A99M and G100S (Table S6).

For the DMF5 and DMF5_YW TCRs, two point mutations designed *in silico* (D26Y on CDR1α and L98W on CDR3β) gave rise to an approximate 400-fold enhancement in affinity.[23] Analysis of the effect of the D26Y mutation (Figure 5C) suggested that the mutation was directly favorable, as well as enhancing the binding to K66 on HLA through the formation of a HB. In the case of L98W (Figure 5D), the burial of a large aromatic residue led to increased contribution to the binding affinity.

For the MEL5_α24β17 aeTCR, a total of 19 mutations (with 17 of these on TCR paratope) gave rise to the approximate 30,000-fold increase in affinity, which was found to be primarily entropically driven.[34] Consistent with this, our simulation analysis indicated that several of the most favorable mutations (CDRα D27F and CDRβ G52P and I53F) increased the total amount of buried hydrophobic content at the binding interface. This is likely an entropically favorable process due to the expulsion of ordered water molecules that typically surround these hydrophobic or aromatic groups upon binding. The only mutation that showed a large negative effect on the binding energy was V93D CDR3α (Figures 5E and 5G). This mutation, however, results in a new interloop HB with R96 (K96 in the WT MEL5 TCR). Similar to the G97D mutation observed in some of the 1G4 eaTCRs, the mutation might play a role in rigidifying the apo-loop (reducing the entropic penalty toward binding), but also likely strengthens neighboring interactions, through preorganization of the loop toward its bound conformation.

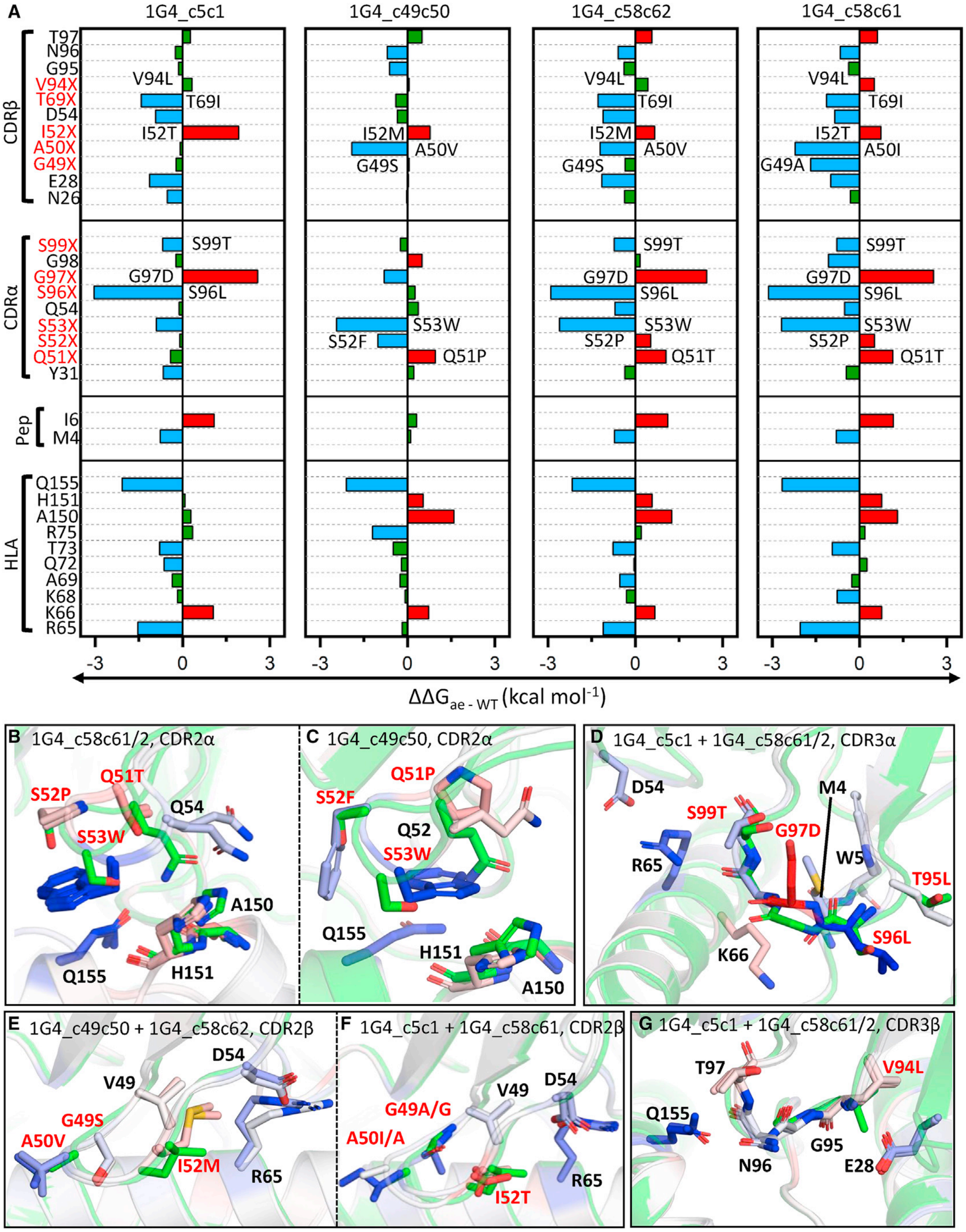

Figure 4. The 1G4 aeTCRs Show Largely Additive Energetic Effects upon Affinity Enhancement

(A) Per-residue ΔG differences as obtained from MMGBSA analysis between the aeTCR variants and 1G4 TCRs (i.e., $\Delta\Delta G$), with positions mutated indicated throughout in red. $\Delta\Delta G$ differences between the 1G4 TCR and aeTCRs are colored blue when $\leq$0.5 kcal mol^{-1} (favorable for binding) and red when >0.5 kcal mol^{-1} (unfavorable for binding), with values in between colored green. (B–G) Color mapping of the above per residue $\Delta\Delta G$ values onto all carbon atoms of the aeTCRs (with the 1G4 TCR structure shown in green for reference). Color mapping is performed from blue to white to red, with blue indicating a favorable change and red indicating an unfavorable change for the aeTCRs. Figures are divided to focus on the different regions of the TCR subjected to affinity maturation (CDR2α, CDR3α, CDR2β, and CDR3β), and subdivided when mutations are not consistent between aeTCRs. (B) 1G4_c58c61/2, CDR2α; (C) 1G4_c49c50, CDR2α; (D) 1G4_c5c1 + 1G4_c58c61/2, CDR3α; (E) 1G4_c49c50 + 1G4_c58c62, CDR2β; (F) 1G4_c5c1 + 1G4_c58c61, CDR2β; and (G) 1G4_c5c1 + 1G4_c58c61/2, CDR3β. (1G4_c58c61/2 means that both 1G4_c58c61 and 1G4_c58c62 TCRs are shown).

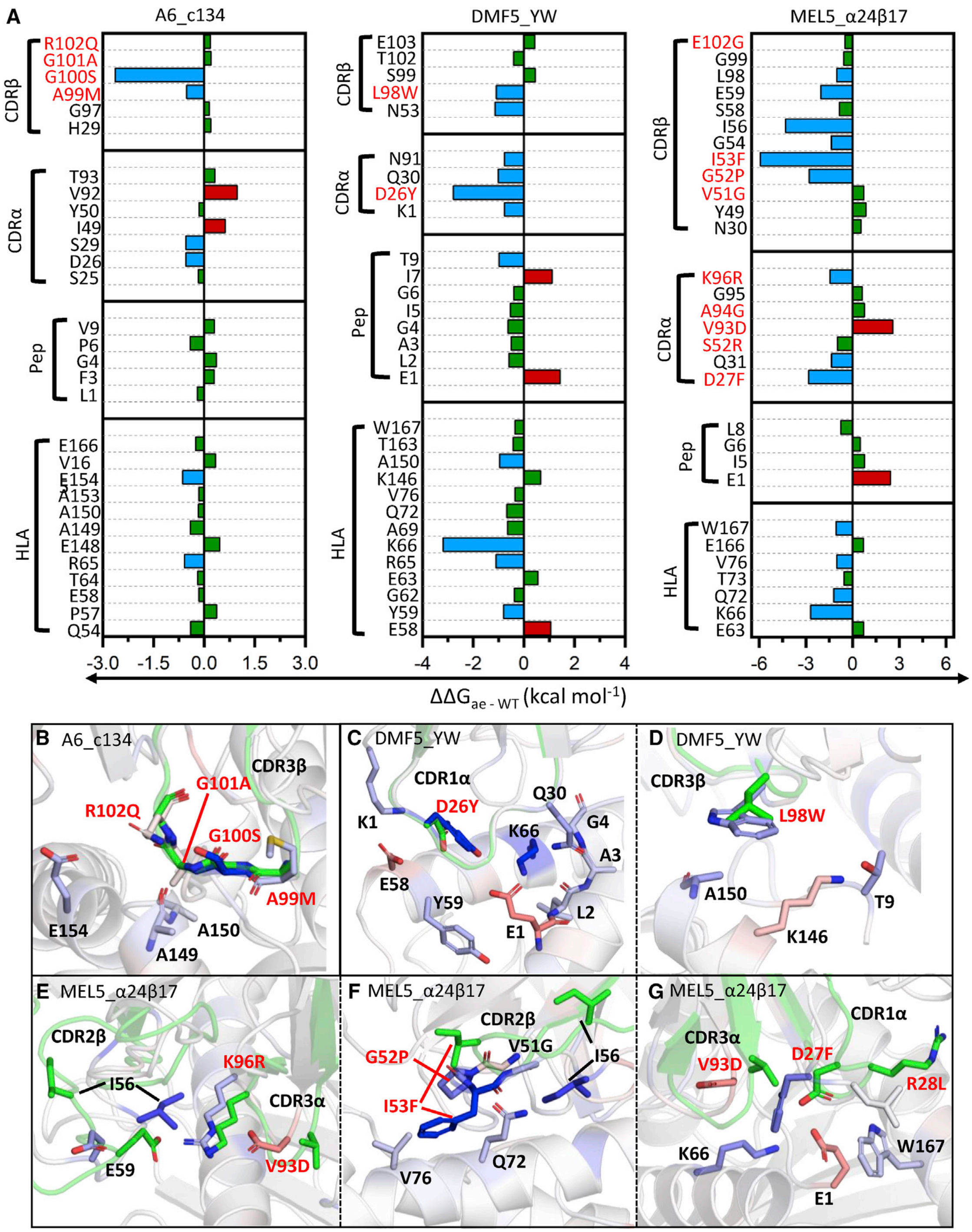

Figure 5. Changes in Energetics at the TCR-pHLA Interface upon Affinity Enhancement of the A6, DMF5, and MEL5 TCRs

(A) Per-residue ΔG differences as obtained from MMGBSA analysis between the A6, DMF5, and MEL5 derived aeTCR variants and their counterpart WT TCRs (i.e., $\Delta\Delta G$), with positions mutated indicated throughout in red. $\Delta\Delta G$ differences between the WT TCR and aeTCR pair are colored blue when ≤ 0.5 kcal mol^{-1} (favorable for aeTCRs) and red when 0.5 kcal mol^{-1} (unfavorable for aeTCRs), with all values in-between colored green. (B–G) Color mapping of the above per residue $\Delta\Delta G$ values onto all carbon atoms of the aeTCRs (with the WT TCR structure shown in green for reference). Color mapping is performed from blue to white to red, with blue indicating a favorable change and red indicating an unfavorable change for the aeTCRs, respectively. Figures are divided up to show the regions which show the major changes upon affinity maturation. (B) A6_c134, CDR3β; (C) DMF5_YW, CDR1α; (D) DMF5_YW, CDR3β; (E) MEL5_α24β17, CDR2β; (F) MEL5_α24β17, CDR2β; and (G) MEL5_α24β17, CDR1α and CDR3α.

Interestingly, of the 19 mutations present, only 9 showed substantial energetic differences. Of the remaining 10 mutations, two positions (CDR1α R28L and CDR3β T100M) made direct and favorable interactions with the pHLA but were predicted to have similar strength in both the MEL5 TCR and the MEL5_α24β17 aeTCR. The other eight mutations did not make direct contact with the pHLA, some being located within the αβ framework interface, and may thus be involved in regulating the flexibility, stability, and/or conformational sampling of the TCR, and therefore indirectly alter the binding affinity.

Affinity-Enhancing Mutations Can Lead to Reductions in Flexibility

The introduction of mutations to enhance affinity could also lead to the rigidification of the TCR and/or TCR-pHLA complex, particularly as some aeTCR residues have been positively selected that are not involved in directly binding pHLA, and instead form inter-chain contacts; e.g., stabilization of 1G4 TCR residue E28 (Figure 4G). To examine this, we calculated the changes in root mean square fluctuation (ΔRMSF) upon affinity enhancement from our MD simulations of the TCRs in their apo (unbound) and pHLA-bound states (Figures 6A and 6B). For the 1G4 aeTCRs, we observed a decrease in the flexibility of the CDR3α loop for the three variants that contained mutations in this loop (1G4_c5c1, 1G4_c58c61, and 1G4_c58c62) (Figure 6A; Figure S4). Consistent with our energetic analysis, this increase in rigidity could be rationalized by the substitution of a glycine residue for a more conformationally restricted amino acid (G97D). Furthermore, the carboxyl side chain of this mutated residue was able to form an interloop HB with T99 (S99 in the WT IG4 TCR), which could further rigidify the loop. An increase in the flexibility of the HV4 α loop was observed for TCR variants 1G4_c49c50, 1G4_c58c62, and 1G4_c58c61, which was likely induced by mutations made in the CDR2α loop given the proximity between these two loops (Figure 6C).

For the MEL5_α24β17 aeTCR, ΔRMSFs indicated that the CDR1α, CDR3α, and CDR2β loops at the TCR binding interface changed significantly upon affinity enhancement (Figure 6B; Figure S5). We note that both the positive and negative changes observed for the CDR2β loop (Figure 6B) are likely the result of the large-scale rearrangement of this loop upon affinity enhancement.[34] Consistent with our observations from the energetic analysis, the increased rigidity observed in both the apo and pHLA-bound forms of the CDR3α loop was likely induced by the formation of a new interloop HB between the side chain of D93 (V93 in the WT MEL5 TCR) and R96 (K96 in the WT MEL5 TCR). In contrast, the CDR1α was only observed to be more rigid in the pHLA-bound form, suggesting increased rigidity of the loop was the result of stronger interactions with the pHLA in MEL5_α24β17 over MEL5. Prior thermodynamic analysis of the MEL5_α24β17 aeTCR demonstrated an improvement in the entropy term of the binding free energy upon affinity maturation compared to MEL5 (from a $T\Delta S^\circ$ of ∼8.3 kcal mol^{-1} to ∼18.1 kcal mol^{-1}).[34] The ΔRMSF data discussed above suggest that this favorable effect was likely not primarily driven through changes in rigidity, but instead through an improved solvation entropy contribution. The A6 TCR and A6_c134 aeTCR demonstrated no substantial changes in flexibility for both the apo and pHLA-bound simulations (Figure 6B; Figure S6). The DMF5 aeTCR showed a reduction in the flexibility of the CDR1α and neighboring CDR2α and HVα loops in the pHLA-bound simulations (Figure 6B; Figure S7). This local reduction in mobility was likely induced by the HB between Y26 in the CDR1α loop and K66 on the HLA, which is present only in the aeTCR.

DISCUSSION

Recent progress in IO has placed treatments such as CAR-T and checkpoint inhibitors firmly in the scientific and media spotlight. These advances have contributed to the development of novel classes of drugs, including soluble bispecific T cell engagers that can target pHLA, as the next generation of cancer IOs.[35] We, and others, have previously demonstrated that it is possible to engineer the natural receptor for pHLA, the TCR, to overcome some of the limitations of the weak natural binding affinity that could limit its use as a soluble therapeutic.[3,20–24] However, how these engineering approaches modulate the natural binding characteristics of thymically selected TCRs is poorly understood. Indeed, previous evidence has shown that T cells engineered with even moderately affinity-enhanced TCRs can cause off-target toxicities through, for example, molecular mimicry of self-antigens.[36,37] Additionally, it has been shown that interactions between the TCR β chain and pHLA can be altered by differential TCR α chain pairing independently of direct TCR α chain-pHLA contacts, suggesting that the TCRVα-Vβ interface can indirectly influence TCR specificity.[38] Thus, the molecular rules that govern affinity maturation of TCRs have strong implications for the design of these molecules as efficacious and safe drugs for cancer treatment.

We used structural analysis, MD simulations, and free energy calculations to comprehensively characterize previously published aeTCRs and solved the structure of a new enhanced affinity TCR-pHLA complex to add to these datasets. These data demonstrate that aeTCRs preserve a native-like TCR binding geometry but achieve affinity enhancement via a range of different energetic mechanisms. Moreover, our free energy calculations reproduce the experimental affinity relationships for all cases studied herein. This demonstrates that atomistic simulations can be used to characterize the "energetic footprint" of different aeTCRs, allowing comparison with their thymically selected WT TCR counterparts. This approach could be used as an *in silico* assay to "filter" potential therapeutic TCR candidates and direct intelligent engineering, to complement *in vitro* experimental specificity/safety validation.

Previously, studies have revealed that aeTCRs can use a range of structural mechanisms during binding. For instance, the A6_c134 aeTCR was characterized by new peptide contacts driven directly through mutated residues;[27] the 1G4_c49c50 aeTCR bound via new HLA interactions mediated by CDR2 loop mutations;[2] the MEL5_α24β17 aeTCR bound via an energetically favorable entropic mechanism driven mainly by new HLA contacts;[34] and the DMF5_YW TCR bound almost identically to its WT counterpart

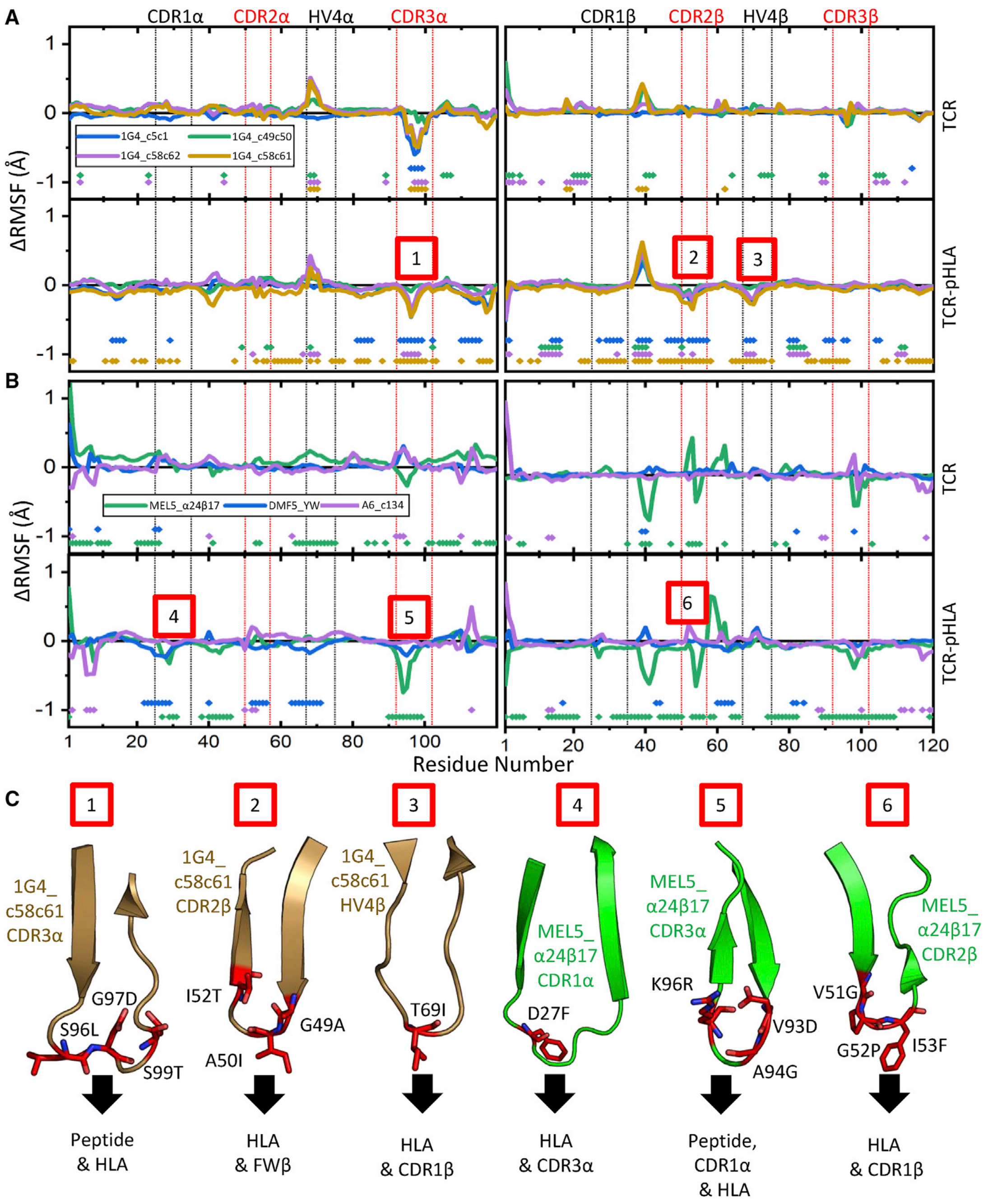

Figure 6. Differences in Flexibility between the aeTCRs Variable Regions and Their Counterpart WT TCRs

(A) ΔRMSF values (aeTCR variant RMSF-WT 1G4 TCR RMSF) for the 1G4-derived aeTCRs, with the top panels corresponding to the CDRα and CDRβ of the apo TCRs, and bottom panels corresponding to CDRα and CDRβ of the TCRs in complex with pHLA. (B) ΔRMSF values (aeTCR variant RMSF-WT TCR RMSF) for the MEL5-, DMF5-, and A6-derived aeTCRs, with top panels corresponding to the CDRα and CDRβ of the apo TCRs, and bottom panels corresponding to CDRα and CDRβ of the TCRs in complex with pHLA. A more negative ΔRMSF value indicates increased rigidity for the aeTCR variant relative to the WT TCR. The points toward the bottom of each graph indicate residues with significantly different ΔRMSF values as determined by a two-sample t test ($p < 0.05$). The numbers boxed in red represent regions of each aeTCR that increase rigidity compared to the WT TCRs in the pHLA bound form. Complete RMSF plots for all TCRs simulated are provided in Figures S4–S7. (C) For each region of the aeTCRs where increased rigidity compared to the WT TCRs in pHLA bound form was observed (marked by numbers in red boxes), the corresponding CDR or HV4 loop of the TCR is shown (as cartoon colored in accordance with A and B) with mutations from WT TCR to aeTCR labeled (shown as red sticks). Black arrows show which regions of the TCR-pHLA complex are near each loop, to provide a potential mechanism for the increases in rigidity detected.

with the increase in affinity attributed to unknown changes in interaction dynamics.[23] Despite the differential mechanisms, aeTCRs can maintain peptide specificity independently of the structural mechanisms guiding their interactions with pHLA.[29] Indeed, it has recently been shown that, using structure-guided approaches, it is possible to improve TCR specificity for a given antigen.[20,39] In this study, our simulations and binding energy calculations showed that the mechanisms underpinning the ability of the aeTCRs to bind to pHLA with enhanced affinity are complex, with indirect and compensatory effects being common. For example, the 1G4 aeTCRs were characterized by energetically favorable effects mediated by direct/indirect peptide and/or HLA interactions with multiple CDR loops, while energetically favorable contributions for the A6_c134 aeTCR were driven almost exclusively through the mutated residues in the CDR3β loop. Furthermore, our analyses indicated that the introduction and burial of large aromatic or hydrophobic side chains at the HLA interface are common in affinity enhancement. This observation ties in with the fact that the most commonly identified residues in protein-protein binding sites are Trp, Met, and Phe.[40] Finally, in both the 1G4 TCR and MEL5 TCR systems, we observed mutations that formed new interloop HBs, resulting in the rigidification of the respective CDR loops of those TCRs (in both the apo and pHLA-bound form). Thus, rationally designing mutations that preorganize the CDR loops for a given antigen may be beneficial for TCR-pHLA affinity.

The data presented raise some interesting biological questions concerning the nature of TCR selection in the thymus, as they demonstrate that the natural TCR scaffold and binding mode is compatible with supra-physiological affinity enhancements.[3,6] If this is the case, why have no natural TCRs ever been detected with such strong affinities? A possible reason could be that the introduction of large aromatic or hydrophobic side chains at the HLA interface, as we observed for the majority of the aeTCRs investigated in this study, could lead to self-reactivity when the TCR is expressed in the context of the T cell surface. Indeed, a recent study demonstrated an enrichment in TCRs with hydrophobic residues in the CDR3 loops in autoimmunity.[41] Additionally, a number of reports have shown that TCRs binding to autoantigens can utilize aromatic residues to directly engage with the autoantigen.[42–44] Thus, natural TCRs with a CDR loop enrichment in residues with large aromatic or hydrophobic side chains (that could have the potential for strong affinity binding) might be deleted in the thymus through negative selection. Finally, weak TCR affinity may enable T cells to rapidly disengage from target cells to allow them to effectively penetrate tissues. This may contribute to the observation that stronger affinity CAR-T cells have a limited ability to penetrate solid tumors.[17]

Our biomolecular simulations successfully ranked the affinity-enhanced variants and identified some commonly adopted strategies for affinity enhancement. The approaches used in this study therefore show early promise for aiding the rational design and implementation of TCR-based therapies. However, prediction of affinity-enhancing TCR mutations might be challenging because the TCR-pHLA system uses a broader and more energetically balanced binding mode than do other immune-related protein-protein interactions (e.g., antibodies, or therapeutic peptides[18,19,45]), resulting in a complex interconnected interface.[45] Furthermore, the peptide repertoire is so diverse that there are likely no fully universal strategies that can be used to affinity enhance TCRs. In any case, it is likely that computational tools will sit alongside other experimental approaches to ensure that therapeutically deployed aeTCRs maintain peptide selectivity relative to their WT parent TCR.[46]

MATERIALS AND METHODS

Cloning, Expression, and Refolding of Proteins

The TCR α and TCR β chains, as well as the HLA class I α chains (tagged and not tagged with a biotinylation sequence) and β_2-microglobulin (β2m) sequences, were cloned, expressed, and refolded as previously described.[47]

Protein Crystallization and Structure Determination

Crystals were grown at 18°C by vapor diffusion via the sitting drop technique. All crystallization screening and optimization experiments were completed with an Art-Robbins Phoenix dispensing robot (Alpha Biotech, UK). 200 nL of 10–15 mg/mL TCR-pHLA complex mixed at a 1:1 molar ratio was added to 200 nL of reservoir solution. Intelli-Plates were then sealed and incubated at 18°C in a crystallization incubator (RuMed, Rubarth Apperate, Germany) and analyzed for crystal formation using the Rock Imager 2 (Formulatrix, Bedford, MA, USA). Crystals selected for further analysis were cryoprotected with ethylene glycol to 25% and then flash cooled in liquid nitrogen in Litho loops (Molecular Dimensions, UK). For α24β17-A2-EAA, optimal crystals were obtained in TOPS[48] with 0.1 M HEPES (pH 7.5), 0.2 M ammonium sulfate, 15% polyethylene glycol (PEG) 4000, and 8.7% glycerol. Diffraction data were collected at several different beamlines at the Diamond Light Source (Oxford, UK) using a Pilatus 2M detector, a QADSC detector, or a Rayonix detector. Using a rotation method, 400 frames were recorded, each covering 0.5° of rotation. Reflection intensities were estimated with the XIA2 package,[49,50] and the data were scaled, reduced, and analyzed with SCALA and the CCP4 package.[51] The TCR-pHLA complex structures were solved with molecular replacement using PHASER.[52] The model sequences were adjusted with COOT[53] and the models refined with REFMAC5. The accession code for α24β17-A2-EAA is PDB: 6TMO.

MD Simulations and Free Energy Calculations

The following is a short overview of the computational methods used herein; a detailed description is provided in the Supplemental Materials and Methods. X-ray crystal structures obtained from multiple studies were used as the starting point for MD simulations of TCRs in both their apo and pHLA-bound states (Table 1; Tables S2 and S3). Following structure preparation, all systems were solvated in a truncated octahedral water box (retaining any crystal waters) large enough to ensure that all protein atoms were at least 10 Å away from the box boundary. Simulations were performed using

Amber16,[54] with the ff14SB force field[55] and TIP3P water model used to describe protein and water molecules, respectively. In order to prepare for production-quality MD simulations in the NPT ensemble (at 300 K and 1 atm), we used a previously described minimization, heating, and equilibration procedure.[56] Subsequently, each system was subjected to 10 replicas of 100-ns-long production MD simulations, with the last 90 ns of each run used for analysis with the software package CPPTRAJ.[57] MMGBSA calculations were performed using MMPBSA.py.MPI,[32] using a protocol (described below) that has previously been shown to provide converged and accurate relative binding free energies for pHLA binding.[58] We performed 25 replicas of 4-ns-long MD simulations per system (separate to the above described 100-ns-long simulations). From each replica, 300 equally spaced snapshots were extracted from the last 3 ns of each MD simulation and subjected to MMGBSA calculations. MMGBSA calculations used the GB-Neck2 (i.e., igb = 8) solvation model and an implicit salt concentration of 150 mM. The obtained results were decomposed into their per-residue contributions to the total binding free energy, with the values obtained used to calculate the differences between the WT and aeTCRs.

Detailed methodology is described in the Supplemental Materials and Methods.

SUPPLEMENTAL INFORMATION

Supplemental Information can be found online at https://doi.org/10.1016/j.omto.2020.07.008.

AUTHOR CONTRIBUTIONS

B.J.M., F.M., C.M., S.H., and P.J.R. performed crystallography and/or structural analysis. R.M.C. performed MD simulations and free energy calculations. D.K.C., R.M.C., S.H., B.J.M., and M.W.v.d.K. wrote the manuscript. D.K.C., M.W.v.d.K., C.R.P., A.K.S., and A.G. funded the study. All authors conceived and directed the project. All authors critiqued the manuscript.

CONFLICTS OF INTEREST

D.K.C., C.J.H., C.M., and S.H., are employees of Immunocore Ltd. The remaining authors declare no competing interests.

ACKNOWLEDGMENTS

R.M.C.'s studentship is funded by the EPSRC. M.W.v.d.K. is a BBSRC David Phillips Fellow (BB/M026280/1). B.J.M. was a CRUK-funded PhD student with additional funding from the Wales Cancer Research Centre. F.M. was a Tenovos Foundation-funded PhD student. A.K.S. is a Wellcome Senior Investigator (WT100327MA). D.K.C. was a Wellcome Career Development Fellow (WT095767). This research made use of the Balena High Performance Computing (HPC) Service at the University of Bath as well as the computational facilities of the Advanced Computing Research Centre of the University of Bristol. The authors would like to thank Diamond Light Source for beamtime (mx6232-3), and the staff of beamlines I02 for assistance with crystal testing and data collection.

REFERENCES

1. de Souza, J.E.S., Galante, P.A.F., de Almeida, R.V.B., da Cunha, J.P.C., Ohara, D.T., Ohno-Machado, L., Old, L.J., and de Souza, S.J. (2012). SurfaceomeDB: a cancer-orientated database for genes encoding cell surface proteins. Cancer Immun. *12*, 15.
2. Dunn, S.M., Rizkallah, P.J., Baston, E., Mahon, T., Cameron, B., Moysey, R., Gao, F., Sami, M., Boulter, J., Li, Y., and Jakobsen, B.K. (2006). Directed evolution of human T cell receptor CDR2 residues by phage display dramatically enhances affinity for cognate peptide-MHC without increasing apparent cross-reactivity. Protein Sci. *15*, 710–721.
3. Li, Y., Moysey, R., Molloy, P.E., Vuidepot, A.-L., Mahon, T., Baston, E., Dunn, S., Liddy, N., Jacob, J., Jakobsen, B.K., and Boulter, J.M. (2005). Directed evolution of human T-cell receptors with picomolar affinities by phage display. Nat. Biotechnol. *23*, 349–354.
4. Boulter, J.M., Glick, M., Todorov, P.T., Baston, E., Sami, M., Rizkallah, P., and Jakobsen, B.K. (2003). Stable, soluble T-cell receptor molecules for crystallization and therapeutics. Protein Eng. *16*, 707–711.
5. Oates, J., and Jakobsen, B.K. (2013). ImmTACs: novel bi-specific agents for targeted cancer therapy. OncoImmunology *2*, e22891.
6. Liddy, N., Bossi, G., Adams, K.J., Lissina, A., Mahon, T.M., Hassan, N.J., Gavarret, J., Bianchi, F.C., Pumphrey, N.J., Ladell, K., et al. (2012). Monoclonal TCR-redirected tumor cell killing. Nat. Med. *18*, 980–987.
7. Cole, D.K., Pumphrey, N.J., Boulter, J.M., Sami, M., Bell, J.I., Gostick, E., Price, D.A., Gao, G.F., Sewell, A.K., and Jakobsen, B.K. (2007). Human TCR-binding affinity is governed by MHC class restriction. J. Immunol. *178*, 5727–5734.
8. Stepanek, O., Prabhakar, A.S.A.S., Osswald, C., King, C.G.C.G., Bulek, A., Naeher, D., Beaufils-Hugot, M., Abanto, M.L., Galati, V., Hausmann, B., et al. (2014). Coreceptor scanning by the T cell receptor provides a mechanism for T cell tolerance. Cell *159*, 333–345.
9. Aleksic, M., Liddy, N., Molloy, P.E., Pumphrey, N., Vuidepot, A., Chang, K.-M., and Jakobsen, B.K. (2012). Different affinity windows for virus and cancer-specific T-cell receptors: implications for therapeutic strategies. Eur. J. Immunol. *42*, 3174–3179.
10. Valitutti, S., Müller, S., Cella, M., Padovan, E., and Lanzavecchia, A. (1995). Serial triggering of many T-cell receptors by a few peptide-MHC complexes. Nature *375*, 148–151.
11. Schmid, D.A., Irving, M.B., Posevitz, V., Hebeisen, M., Posevitz-Fejfar, A., Sarria, J.-C.F., Gomez-Eerland, R., Thome, M., Schumacher, T.N.M., Romero, P., et al. (2010). Evidence for a TCR affinity threshold delimiting maximal CD8 T cell function. J. Immunol. *184*, 4936–4946.
12. Tan, M.P., Gerry, A.B., Brewer, J.E., Melchiori, L., Bridgeman, J.S., Bennett, A.D., Pumphrey, N.J., Jakobsen, B.K., Price, D.A., Ladell, K., and Sewell, A.K. (2015). T cell receptor binding affinity governs the functional profile of cancer-specific CD8+ T cells. Clin. Exp. Immunol. *180*, 255–270.
13. Mason, D. (1998). A very high level of crossreactivity is an essential feature of the T-cell receptor. Immunol. Today *19*, 395–404.
14. Sewell, A.K. (2012). Why must T cells be cross-reactive? Nat. Rev. Immunol. *12*, 669–677.
15. Wooldridge, L., Ekeruche-Makinde, J., van den Berg, H.A., Skowera, A., Miles, J.J., Tan, M.P., Dolton, G., Clement, M., Llewellyn-Lacey, S., Price, D.A., et al. (2012). A single autoimmune T cell receptor recognizes more than a million different peptides. J. Biol. Chem. *287*, 1168–1177.
16. Cole, D.K., van den Berg, H.A., Lloyd, A., Crowther, M.D., Beck, K., Ekeruche-Makinde, J., Miles, J.J., Bulek, A.M., Dolton, G., Schauenburg, A.J., et al. (2017). Structural mechanism underpinning cross-reactivity of a $CD8^+$ T-cell clone that recognizes a peptide derived from human telomerase reverse transcriptase. J. Biol. Chem. *292*, 802–813.
17. Moon, E.K., Wang, L.-C., Dolfi, D.V., Wilson, C.B., Ranganathan, R., Sun, J., Kapoor, V., Scholler, J., Puré, E., Milone, M.C., et al. (2014). Multifactorial T-cell hypofunction that is reversible can limit the efficacy of chimeric antigen receptor-transduced human T cells in solid tumors. Clin. Cancer Res. *20*, 4262–4273.
18. Ministro, J., Manuel, A.M., and Goncalves, J. (2020). Therapeutic antibody engineering and selection strategies. Adv. Biochem. Eng. Biotechnol. *171*, 55–86.

19. Saw, P.E., and Song, E.-W. (2019). Phage display screening of therapeutic peptide for cancer targeting and therapy. Protein Cell *10*, 787–807.

20. Harris, D.T., Wang, N., Riley, T.P., Anderson, S.D., Singh, N.K., Procko, E., Baker, B.M., and Kranz, D.M. (2016). Deep mutational scans as a guide to engineering high affinity T cell receptor interactions with peptide-bound major histocompatibility complex. J. Biol. Chem. *291*, 24566–24578.

21. Chervin, A.S., Aggen, D.H., Raseman, J.M., and Kranz, D.M. (2008). Engineering higher affinity T cell receptors using a T cell display system. J. Immunol. Methods *339*, 175–184.

22. Richman, S.A., Healan, S.J., Weber, K.S., Donermeyer, D.L., Dossett, M.L., Greenberg, P.D., Allen, P.M., and Kranz, D.M. (2006). Development of a novel strategy for engineering high-affinity proteins by yeast display. Protein Eng. Des. Sel. *19*, 255–264.

23. Pierce, B.G., Hellman, L.M., Hossain, M., Singh, N.K., Vander Kooi, C.W., Weng, Z., and Baker, B.M. (2014). Computational design of the affinity and specificity of a therapeutic T cell receptor. PLoS Comput. Biol. *10*, e1003478.

24. Haidar, J.N., Pierce, B., Yu, Y., Tong, W., Li, M., and Weng, Z. (2009). Structure-based design of a T-cell receptor leads to nearly 100-fold improvement in binding affinity for pepMHC. Proteins *74*, 948–960.

25. Zoete, V., Irving, M.B., and Michielin, O. (2010). MM-GBSA binding free energy decomposition and T cell receptor engineering. J. Mol. Recognit. *23*, 142–152.

26. Sharma, P., and Kranz, D.M. (2018). Subtle changes at the variable domain interface of the T-cell receptor can strongly increase affinity. J. Biol. Chem. *293*, 1820–1834.

27. Cole, D.K., Sami, M., Scott, D.R., Rizkallah, P.J., Borbulevych, O.Y., Todorov, P.T., Moysey, R.K., Jakobsen, B.K., Boulter, J.M., Baker, B.M., and Yi Li. (2013). Increased peptide contacts govern high affinity binding of a modified TCR whilst maintaining a native pMHC docking mode. Front. Immunol. *4*, 168.

28. Sami, M., Rizkallah, P.J., Dunn, S., Molloy, P., Moysey, R., Vuidepot, A., Baston, E., Todorov, P., Li, Y., Gao, F., et al. (2007). Crystal structures of high affinity human T-cell receptors bound to peptide major histocompatibility complex reveal native diagonal binding geometry. Protein Eng. Des. Sel. *20*, 397–403.

29. Cole, D.K., Miles, K.M., Madura, F., Holland, C.J., Schauenburg, A.J.A.A., Godkin, A.J., Bulek, A.M., Fuller, A., Akpovwa, H.J., Pymm, P.G., et al. (2014). T-cell receptor (TCR)-peptide specificity overrides affinity-enhancing TCR-major histocompatibility complex interactions. J. Biol. Chem. *289*, 628–638.

30. Knapp, B., Ospina, L., and Deane, C.M. (2018). Avoiding false positive conclusions in molecular simulation: the importance of replicas. J. Chem. Theory Comput. *14*, 6127–6138.

31. Siebenmorgen, T., and Zacharias, M. (2020). Computational prediction of protein–protein binding affinities. WIREs Comput. Mol. Sci. *10*, e1448.

32. Miller, B.R., 3rd, McGee, T.D., Jr., Swails, J.M., Homeyer, N., Gohlke, H., and Roitberg, A.E. (2012). *MMPBSA.py*: an efficient program for end-state free energy calculations. J. Chem. Theory Comput. *8*, 3314–3321.

33. Genheden, S., and Ryde, U. (2015). The MM/PBSA and MM/GBSA methods to estimate ligand-binding affinities. Expert Opin. Drug Discov. *10*, 449–461.

34. Madura, F., Rizkallah, P.J., Miles, K.M., Holland, C.J., Bulek, A.M., Fuller, A., Schauenburg, A.J., Miles, J.J., Liddy, N., Sami, M., et al. (2013). T-cell receptor specificity maintained by altered thermodynamics. J. Biol. Chem. *288*, 18766–18775.

35. Goebeler, M.-E., and Bargou, R.C. (2020). T cell-engaging therapies—BiTEs and beyond. Nat. Rev. Clin. Oncol. *17*, 418–434.

36. Cameron, B.J., Gerry, A.B., Dukes, J., Harper, J.V., Kannan, V., Bianchi, F.C., Grand, F., Brewer, J.E., Gupta, M., Plesa, G., et al. (2013). Identification of a Titin-derived HLA-A1-presented peptide as a cross-reactive target for engineered MAGE A3-directed T cells. Sci. Transl. Med. *5*, 197ra103.

37. Raman, M.C.C., Rizkallah, P.J., Simmons, R., Donnellan, Z., Dukes, J., Bossi, G., Le Provost, G.S., Todorov, P., Baston, E., Hickman, E., et al. (2016). Direct molecular mimicry enables off-target cardiovascular toxicity by an enhanced affinity TCR designed for cancer immunotherapy. Sci. Rep. *6*, 18851.

38. Stadinski, B.D., Trenh, P., Smith, R.L., Bautista, B., Huseby, P.G., Li, G., Stern, L.J., and Huseby, E.S. (2011). A role for differential variable gene pairing in creating T cell receptors specific for unique major histocompatibility ligands. Immunity *35*, 694–704.

39. Hellman, L.M., Foley, K.C., Singh, N.K., Alonso, J.A., Riley, T.P., Devlin, J.R., Ayres, C.M., Keller, G.L.J., Zhang, Y., Vander Kooi, C.W., et al. (2019). Improving T cell receptor on-target specificity via structure-guided design. Mol. Ther. *27*, 300–313.

40. Ma, B., and Nussinov, R. (2007). Trp/Met/Phe hot spots in protein-protein interactions: potential targets in drug design. Curr. Top. Med. Chem. *7*, 999–1005.

41. Stadinski, B.D., Shekhar, K., Gómez-Touriño, I., Jung, J., Sasaki, K., Sewell, A.K., Peakman, M., Chakraborty, A.K., and Huseby, E.S. (2016). Hydrophobic CDR3 residues promote the development of self-reactive T cells. Nat. Immunol. *17*, 946–955.

42. Bulek, A.M., Cole, D.K., Skowera, A., Dolton, G., Gras, S., Madura, F., Fuller, A., Miles, J.J., Gostick, E., Price, D.A., et al. (2012). Structural basis for the killing of human beta cells by $CD8^+$ T cells in type 1 diabetes. Nat. Immunol. *13*, 283–289.

43. Cole, D.K., Bulek, A.M., Dolton, G., Schauenberg, A.J., Szomolay, B., Rittase, W., Trimby, A., Jothikumar, P., Fuller, A., Skowera, A., et al. (2016). Hotspot autoimmune T cell receptor binding underlies pathogen and insulin peptide cross-reactivity. J. Clin. Invest. *126*, 2191–2204.

44. Sethi, D.K., Gordo, S., Schubert, D.A., and Wucherpfennig, K.W. (2013). Crossreactivity of a human autoimmune TCR is dominated by a single TCR loop. Nat. Commun. *4*, 2623.

45. Holland, C.J., Crean, R.M., Pentier, J.M., de Wet, B., Lloyd, A., Srikannathasan, V., Lissin, N., Lloyd, K.A., Blicher, T.H., Conroy, P.J., et al. (2020). Specificity of bispecific T cell receptors and antibodies targeting peptide-HLA. J. Clin. Invest. *130*, 2673–2688.

46. Coles, C.H., Mulvaney, R.M., Malla, S., Walker, A., Smith, K.J., Lloyd, A., Lowe, K.L., McCully, M.L., Martinez Hague, R., Aleksic, M., et al. (2020). TCRs with distinct specificity profiles use different binding modes to engage an identical peptide-HLA complex. J. Immunol. *204*, 1943–1953.

47. Cole, D.K., Yuan, F., Rizkallah, P.J., Miles, J.J., Gostick, E., Price, D.A., Gao, G.F., Jakobsen, B.K., and Sewell, A.K. (2009). Germ line-governed recognition of a cancer epitope by an immunodominant human T-cell receptor. J. Biol. Chem. *284*, 27281–27289.

48. Bulek, A.M., Madura, F., Fuller, A., Holland, C.J., Schauenburg, A.J.A., Sewell, A.K., Rizkallah, P.J., and Cole, D.K. (2012). TCR/pMHC optimized protein crystallization screen. J. Immunol. Methods *382*, 203–210.

49. Winter, G., Lobley, C.M.C., and Prince, S.M. (2013). Decision making in *xia*2. Acta Crystallogr. D Biol. Crystallogr. *69*, 1260–1273.

50. Winter, G. (2010). Xia2: an expert system for macromolecular crystallography data reduction. J. Appl. Cryst. *43*, 186–190.

51. Collaborative Computational Project, Number 4 (1994). The CCP4 suite: programs for protein crystallography. Acta Crystallogr. D Biol. Crystallogr. *50*, 760–763.

52. McCoy, A.J., Grosse-Kunstleve, R.W., Adams, P.D., Winn, M.D., Storoni, L.C., and Read, R.J. (2007). Phaser crystallographic software. J. Appl. Cryst. *40*, 658–674.

53. Emsley, P., and Cowtan, K. (2004). Coot: model-building tools for molecular graphics. Acta Crystallogr. D Biol. Crystallogr. *60*, 2126–2132.

54. Case, D.A., Cerutti, D.S., Cheatham, T.E., III, Darden, T.A., Duke, R.E., Giese, T.J., Gohlke, H., Goetz, A.W., Greene, D., Homeyer, N., et al. (2017). AMBER 2016 (San Francisco: University of California).

55. Maier, J.A., Martinez, C., Kasavajhala, K., Wickstrom, L., Hauser, K.E., and Simmerling, C. (2015). ff14SB: improving the accuracy of protein side chain and backbone parameters from ff99SB. J. Chem. Theory Comput. *11*, 3696–3713.

56. Hopkins, J.R., Crean, R.M., Catici, D.A.M., Sewell, A.K., Arcus, V.L., Van der Kamp, M.W., Cole, D.K., and Pudney, C.R. (2020). Peptide cargo tunes a network of correlated motions in human leucocyte antigens. FEBS J. Published online March 5, 2020. https://doi.org/10.1111/febs.15278.

57. Roe, D.R., and Cheatham, T.E., 3rd (2013). PTRAJ and CPPTRAJ: software for processing and analysis of molecular dynamics trajectory data. J. Chem. Theory Comput. *9*, 3084–3095.

58. Wan, S., Knapp, B., Wright, D.W., Deane, C.M., and Coveney, P.V. (2015). Rapid, precise, and reproducible prediction of peptide-MHC binding affinities from molecular dynamics that correlate well with experiment. J. Chem. Theory Comput. *11*, 3346–3356.

59. Chen, J.-L., Stewart-Jones, G., Bossi, G., Lissin, N.M., Wooldridge, L., Choi, E.M.L., Held, G., Dunbar, P.R., Esnouf, R.M., Sami, M., et al. (2005). Structural and kinetic basis for heightened immunogenicity of T cell vaccines. J. Exp. Med. *201*, 1243–1255.

60. Borbulevych, O.Y., Santhanagopolan, S.M., Hossain, M., and Baker, B.M. (2011). TCRs used in cancer gene therapy cross-react with MART-1/Melan-A tumor antigens via distinct mechanisms. J. Immunol. *187*, 2453–2463.

61. Madura, F., Rizkallah, P.J., Holland, C.J., Fuller, A., Bulek, A., Godkin, A.J., Schauenburg, A.J., Cole, D.K., and Sewell, A.K. (2015). Structural basis for ineffective T-cell responses to MHC anchor residue-improved "heteroclitic" peptides. Eur. J. Immunol. *45*, 584–591.

62. Madura, F., Rizkallah, P.J., Legut, M., Holland, C.J., Fuller, A., Bulek, A., Schauenburg, A.J., Trimby, A., Hopkins, J.R., Wells, S.A., et al. (2019). TCR-induced alteration of primary MHC peptide anchor residue. Eur. J. Immunol. *49*, 1052–1066.

63. Garboczi, D.N., Ghosh, P., Utz, U., Fan, Q.R., Biddison, W.E., and Wiley, D.C. (1996). Structure of the complex between human T-cell receptor, viral peptide and HLA-A2. Nature *384*, 134–141.

OMTO, Volume *18*

Supplemental Information

Molecular Rules Underpinning Enhanced Affinity Binding of Human T Cell Receptors Engineered for Immunotherapy

Rory M. Crean, Bruce J. MacLachlan, Florian Madura, Thomas Whalley, Pierre J. Rizkallah, Christopher J. Holland, Catriona McMurran, Stephen Harper, Andrew Godkin, Andrew K. Sewell, Christopher R. Pudney, Marc W. van der Kamp, and David K. Cole

Title:

Molecular rules underpinning enhanced affinity binding of human T cell receptors engineered for immunotherapy

Authors and Affiliations:

Rory M. Crean[1,2], Bruce J. MacLachlan[3†], Florian Madura[3], Thomas Whalley[3], Pierre J. Rizkallah[3], Christopher J. Holland[4], Catriona McMurran[4], Stephen Harper[4], Andrew Godkin[3], Andrew K. Sewell[3], Christopher R. Pudney[1,5], Marc W. van der Kamp[6*], and David K. Cole[3,4*]

[1]Department of Biology and Biochemistry, University of Bath, Bath, BA2 7AY, UK.
[2]Doctoral Training Centre in Sustainable Chemical Technologies, University of Bath, Bath, BA2 7AY, UK.
[3]Division of Infection & Immunity, Cardiff University, CF14 4XN, UK.
[4]Immunocore Ltd., Abingdon OX14 4RY, UK
[5]Centre for Therapeutic Innovation, University of Bath, Bath, BA2 7AY, UK.
[6]School of Biochemistry, University of Bristol, Biomedical Sciences Building, University Walk, Bristol, BS8 1TD, UK.

[†]Present address: Monash Biomedicine Discovery Institute, 19 Innovation Walk, Clayton, Victoria, 3800, Australia

*These authors contributed equally to the study

To whom correspondence should be addressed: Dr Marc W. van der Kamp Email: marc.vanderkamp@bristol.ac.uk, and Dr David K. Cole, E-mail: david.cole@immunocore.com.

Supplementary Figures

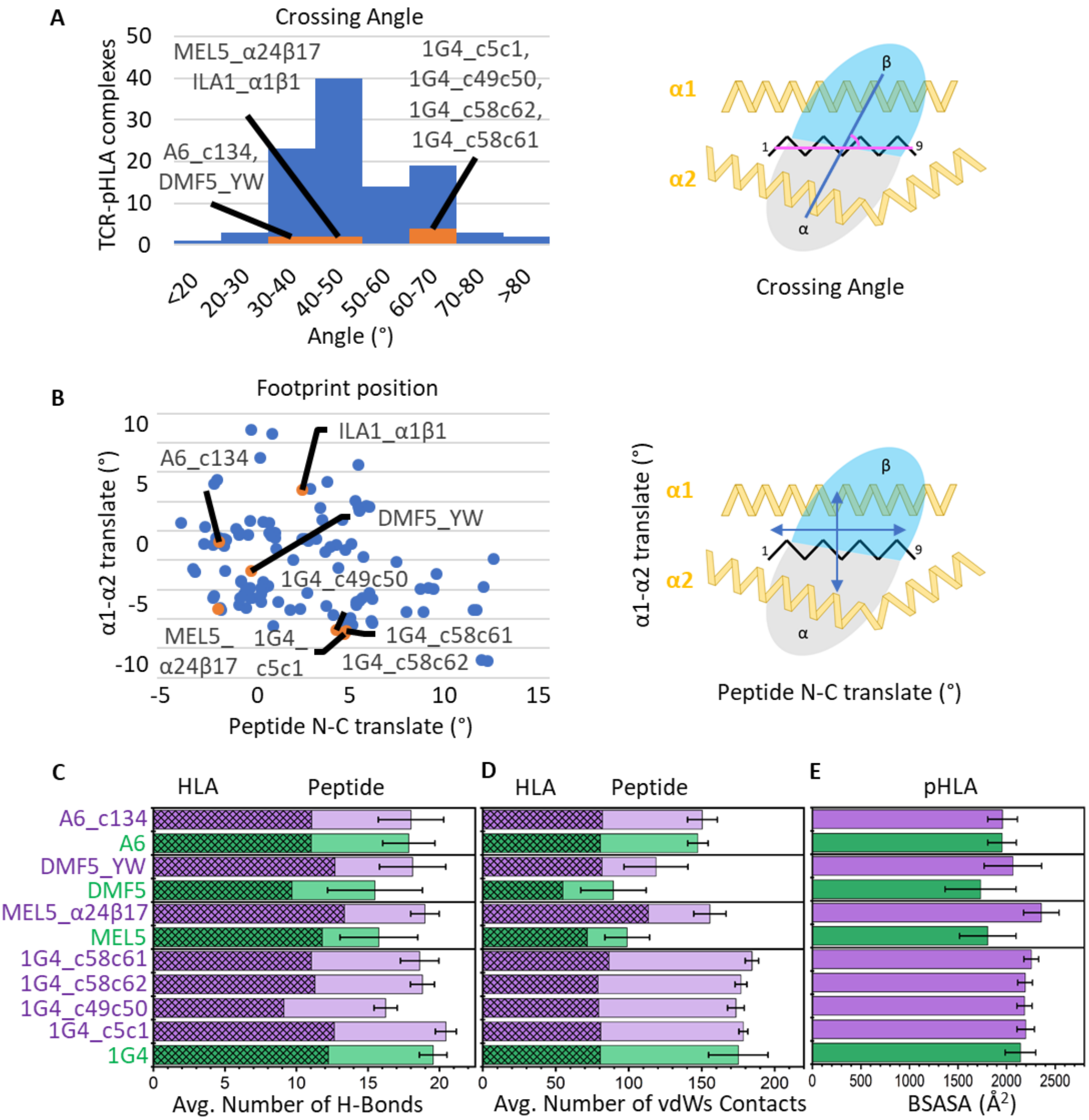

Supplementary Figure S1: (**A**) Distribution of TCR crossing angles of all published TCR-pHLA complexes are plotted in blue. Affinity enhanced TCR-pHLA complexes are labelled and shown in the plot in orange. (**B**) Footprint position of all published TCR-pHLA complexes are plotted in blue. Affinity enhanced TCR-pHLA complexes are labelled and shown in the plot in orange. (**C-E**) Differences in the average number of HBs (**C**), vdWs contacts (**D**) and the BSASA (**E**) from our MD simulations. All wildtype TCRs are coloured in green and all affinity enhanced TCRs are coloured in purple. Bars for the HBs and vdWs contacts are divided in two based on TCR-HLA interactions (darker colour and hashed bars) and peptide-TCR interactions (lighter colour). The totals obtained are from 10 independent 100 ns long MD simulations of each TCR-pHLA complex (using the last 90 ns of each simulation). Error bars plotted for A and B are the standard deviation of the averages from the 10 replicas. Error bars plotted for C are the standard deviation obtained from combining all snapshots from all replicas together.

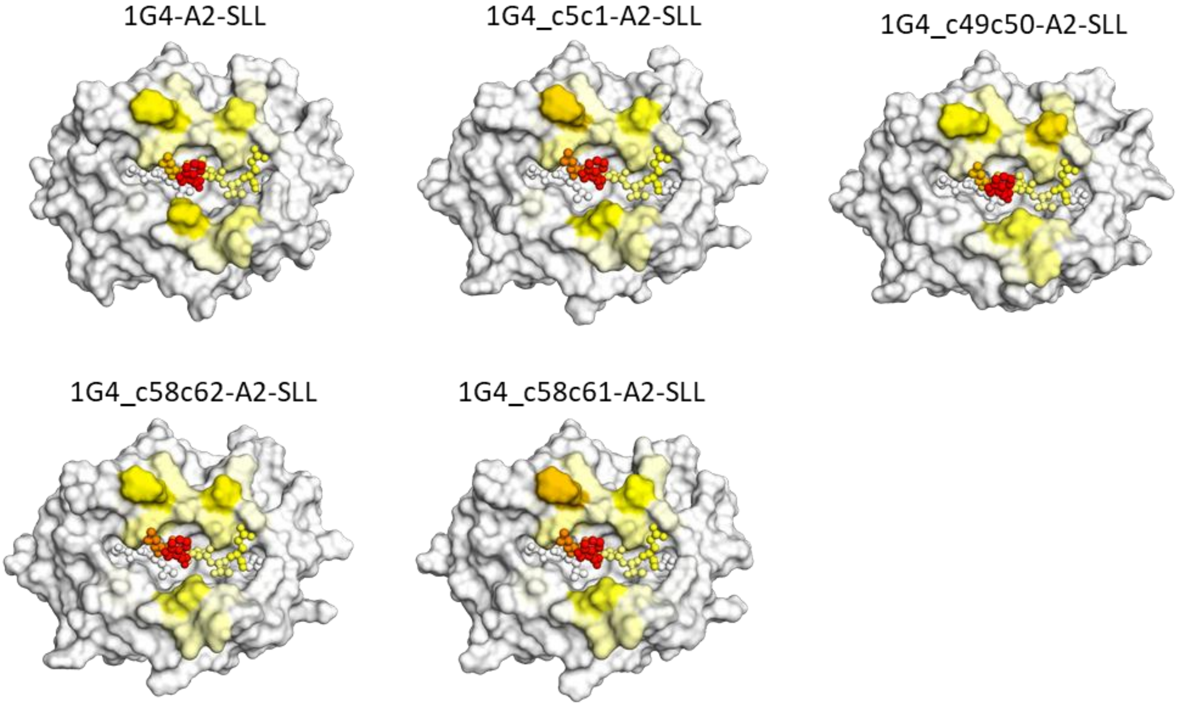

Supplementary Figure S2: Surface plots of the pHLA (peptide shown as sticks) with each structure colour mapped according the average number of vdWs contacts formed between the given residue and all five 1G4 affinity enhanced TCRs studied in this manuscript. Colour mapping was performed from white (no contacts) through yellow and orange to red (highest number of contacts observed for each of the pairs of TCR-pHLAs studied). All pHLA structures are shown in the same orientation, such that the peptide N-terminus is left and the C-terminus is right.

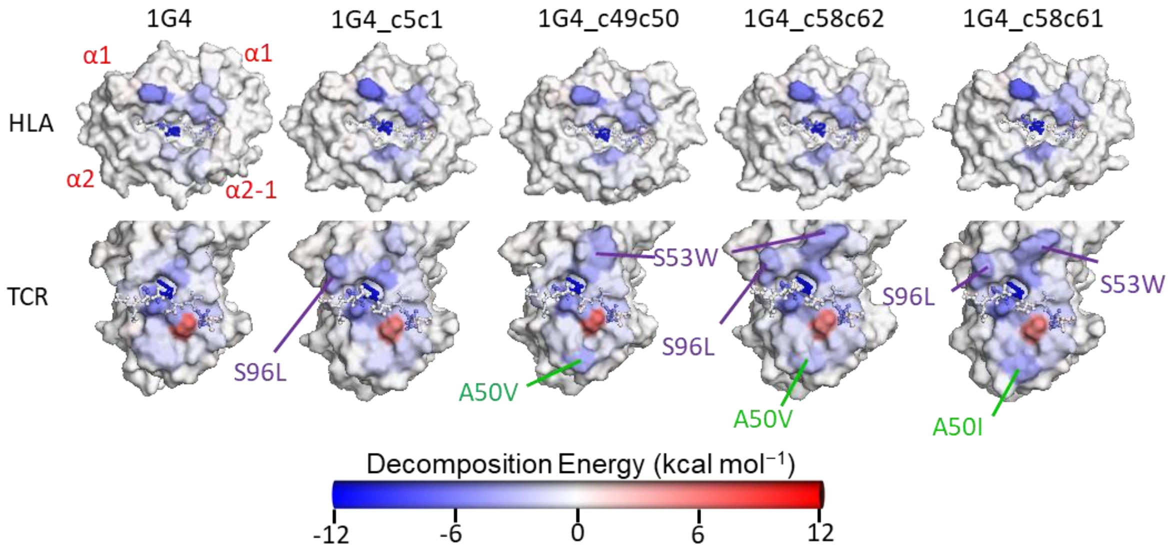

Supplementary Figure S3: Changes in the energetic footprint between the wildtype 1G4 and affinity enhanced 1G4 TCRs. For all TCR-pHLA complexes, the HLA (top) and TCR (bottom) structures are plotted as surfaces with the peptide shown in both structures as sticks. All plots are colour mapped according to the MMGBSA per residue decomposition results, going from blue (favours binding) to white (no preference) to red (disfavours binding). All pHLA and TCR structures are shown in the same orientation, such that the peptide N-terminus is left and the C-terminus right. Several mutations sites are indicated on the affinity enhanced variants (purple labels: CDRα mutations; green labels: CDRβ mutations).

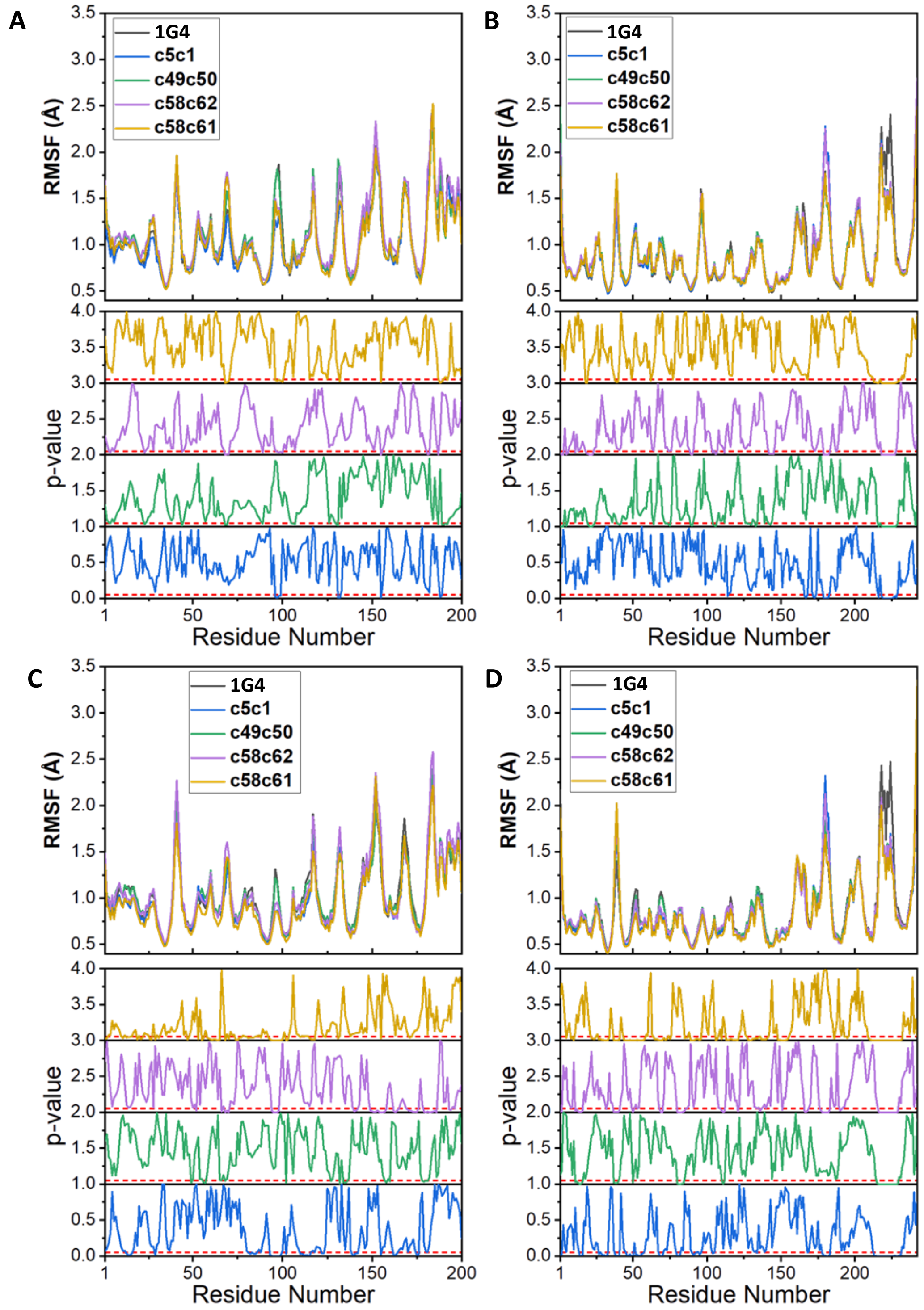

Supplementary Figure S4: Cα RMSF values for all Apo (A + B) and pHLA bound (C + D) 1G4 TCRs simulated. The α-chain RMSFs for the apo and pHLA bound simulations are plotted in panels A and C respectively, whilst the β-chain RMSFs for the apo and pHLA bound simulations are plotted in panels B and D respectively. Below each plot is the p-value obtained from a two-sample t-test between the wildtype and each affinity enhanced 1G4 TCR (following the same colour scheme). A red dotted line is plotted at a p-value of 0.05, which is the cut-off used to determine significance.

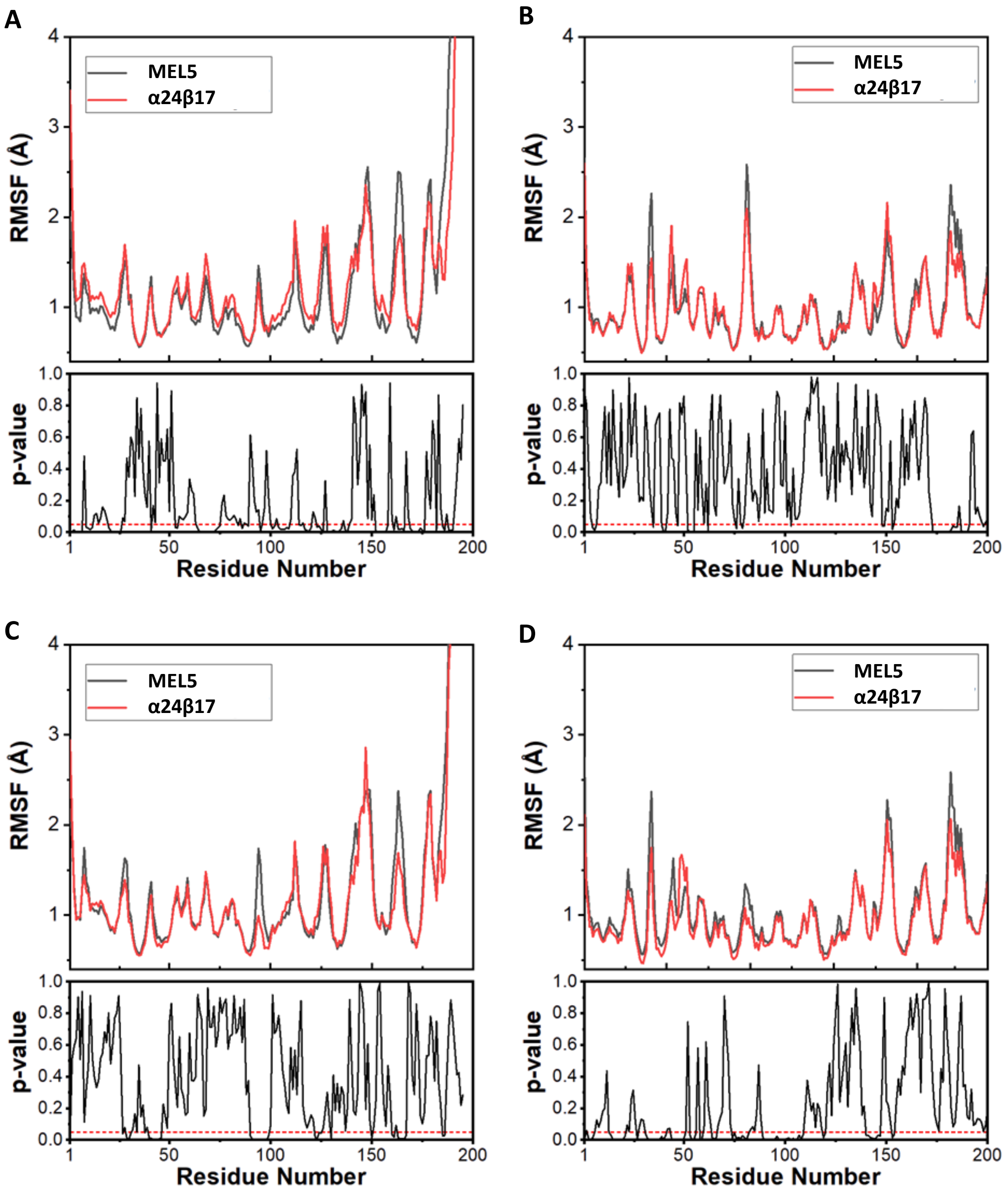

Supplementary Figure S5: Cα RMSF values for both Apo (A + B) and pHLA bound (C + D) MEL5 TCRs simulated. The α-chain RMSFs for the apo and pHLA bound simulations are plotted in panels A and C respectively, whilst the β-chain RMSFs for the apo and pHLA bound simulations are plotted in panels B and D respectively. Below each plot is the p-value obtained from a two-sample t-test between the wildtype and the affinity enhanced MEL5 TCRs. A red dotted line is plotted at a p-value of 0.05, which is the cut-off used to determine significance.

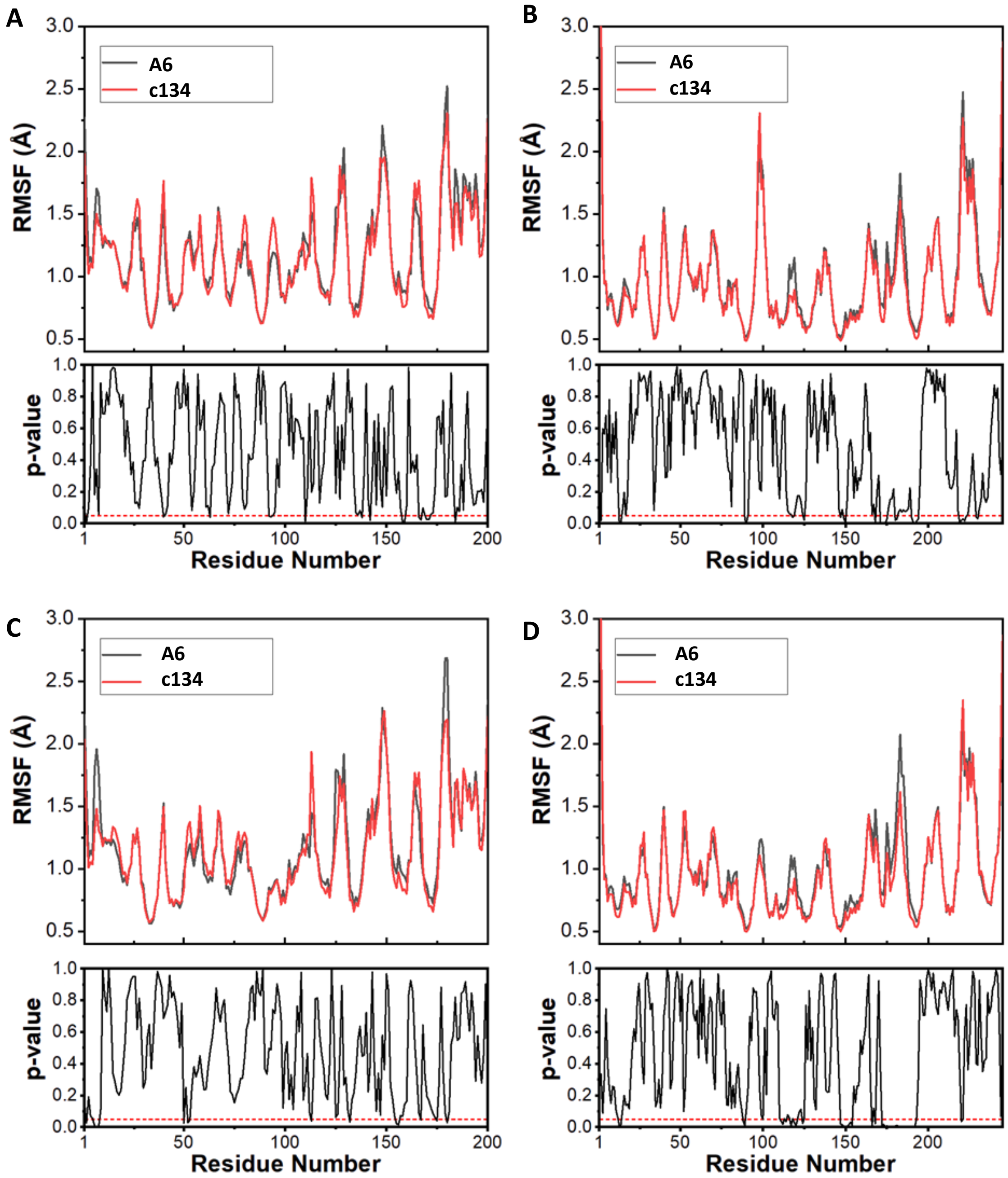

Supplementary Figure S6: Cα RMSF values for both Apo (A + B) and pHLA bound (C + D) Tax A6 TCRs simulated. The α-chain RMSFs for the apo and pHLA bound simulations are plotted in panels A and C respectively, whilst the β-chain RMSFs for the apo and pHLA bound simulations are plotted in panels B and D respectively. Below each plot is the p-value obtained from a two-sample t-test between the wildtype and the affinity enhanced A6 TCRs. A red dotted line is plotted at a p-value of 0.05, which is the cut-off used to determine significance.

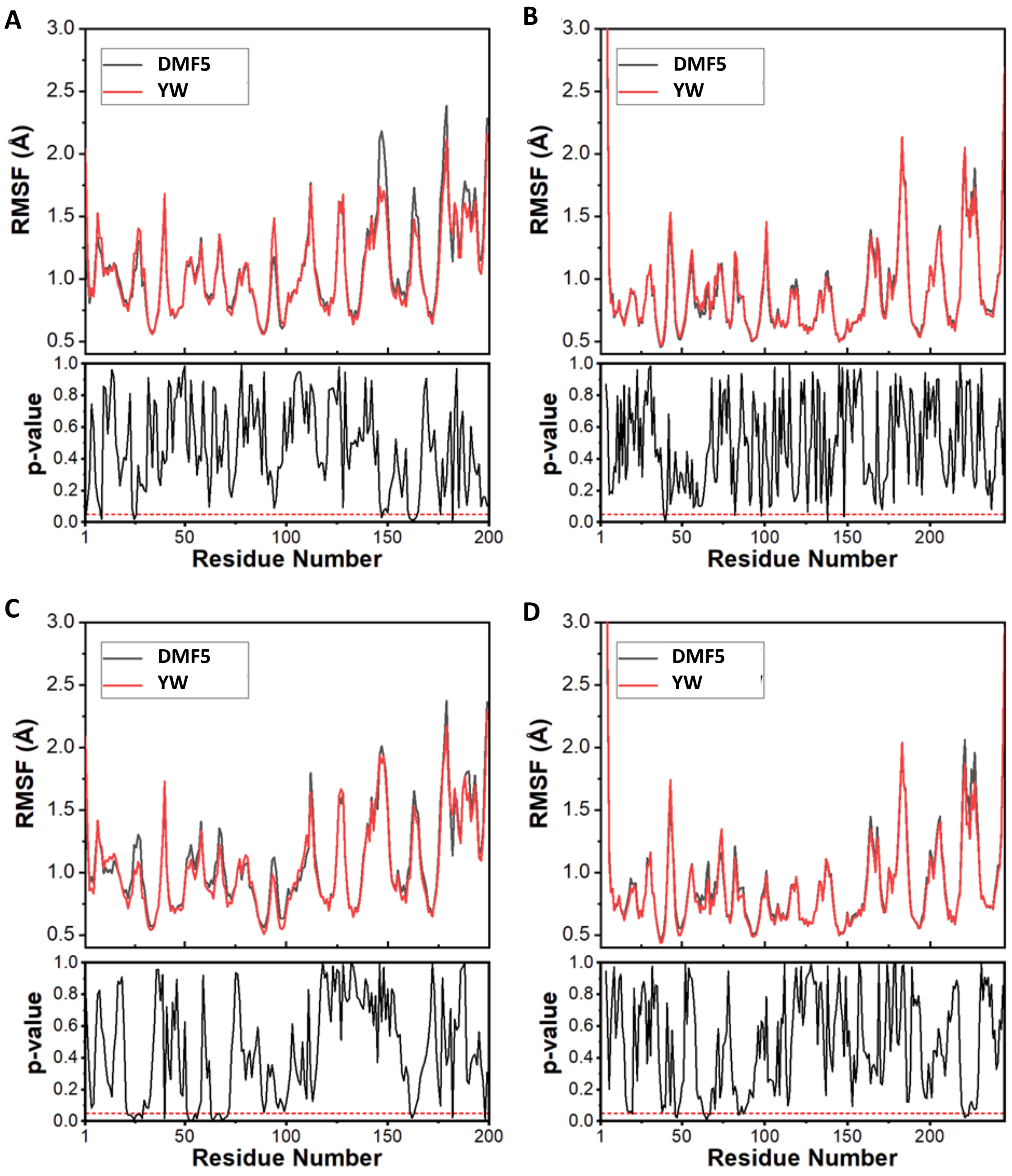

Supplementary Figure S7: Cα RMSF values for both Apo (A + B) and pHLA bound (C + D) DMF5 TCRs simulated. The α-chain RMSFs for the apo and pHLA bound simulations are plotted in panels A and C respectively, whilst the β-chain RMSFs for the apo and pHLA bound simulations are plotted in panels B and D respectively. Below each plot is the p-value obtained from a two-sample t-test between the wildtype and the affinity enhanced DMF5 TCRs. A red dotted line is plotted at a p-value of 0.05, which is the cut-off used to determine significance.

Supplementary Tables

Supplementary Table S1. Data collection and refinement statistics

* One crystal was used for determining each structure.
* Figures in brackets refer to outer resolution shell, where applicable.
[1] Coordinate Estimated Standard Uncertainty in (Å), based on maximum likelihood statistics.

**Data Collection**	**MEL5_α24β17-A2-EAA**
Accession Code	6TMO
Beamtime proposal	mx6232-3
Diamond Beamline	I02
Wavelength	0.9795
**Crystal Data**	
a, b, c (Å)	121.49, 121.49, 82.68
α, β, γ (°)	90.0, 90.0, 90.0
Space group	$P\ 4_3$
Resolution (Å)	2.10 – 54.33
Outer shell	2.10 – 2.15
R-merge (%)	8.4 (71.3)
R-meas (%)	9.6 (80.7)
R-pim (%)	3.3 (27.7)
CC1/2	n/a (n/a)
I / σ(I)	16.0 (3.6)
Completeness (%)	99.4 (99.0)
Multiplicity	8.3 (8.4)
Total Measurements	581,617 (42,829)
Unique Reflections	69,920 (5,110)
Wilson B-factor ($Å^2$)	33.3
**Refinement Statistics**	
Non-H Atoms	7,167
R-work reflections	66,386
R-free reflections	3,532
R-work/R-free	0.173 / 0.209
**rms deviations**	
Bond lengths (Å)	0.016
Bond Angles (°)	1.556
[1]Coordinate error	0.093
Mean B value ($Å^2$)	40.3
**Ramachandran Statistics**	
Favoured/allowed/Outliers	775 / 24 / 6
%	96.3 / 3.0 / 0.7

Supplementary Table S2. PDB IDs and histidine tautomerisation state assignments for all 1G4 MD simulations.

[a], All Apo-TCR simulations used the same tautomerisation states as those in the TCR-pHLA simulations.

[b], HID corresponds to a histidine residue which is singly protonated on its Nδ1 nitrogen.

[c], HIE corresponds to a histidine residue which is singly protonated on their Nε2 nitrogen.

**TCR-pHLA System**[a]	**HID Tautomerisation States**[b]	**HIE Tautomerisation States**[c]
**1G4**<br>PDB: 2BNR	**HLA:** 3, 70, 74, 93, 114, 145, 260.<br>**β2m:**<br>**CDRα:**<br>**CDRβ:** 151.	**HLA:** 151, 188, 191, 192, 197, 263.<br>**β2m:** 13, 31, 51, 84.<br>**CDRα:** 112.<br>**CDRβ:** 27, 45, 134, 164, 204.
**1G4_c5c1**<br>PDB: 2PYE	**HLA:** 3, 70, 74, 93, 114, 145, 260.<br>**β2m:**<br>**CDRα:**<br>**CDRβ:** 151.	**HLA:** 151, 188, 191, 192, 197, 263.<br>**β2m:** 13, 31, 51, 84.<br>**CDRα:** 112.<br>**CDRβ:** 27, 45, 134, 164, 204.
**1G4_c49c50**<br>PDB: 2F53	**HLA:** 3, 70, 74, 93, 114, 145, 260.<br>**β2m:**<br>**CDRα:**<br>**CDRβ:** 151.	**HLA:** 151, 188, 191, 192, 197, 263.<br>**β2m:** 13, 31, 51, 84.<br>**CDRα:** 112.<br>**CDRβ:** 27, 45, 134, 164, 204.
**1G4_c58c62**<br>PDB: 2P5W	**HLA:** 3, 70, 74, 93, 114, 145, 260.<br>**β2m:**<br>**CDRα:**<br>**CDRβ:** 151.	**HLA:** 151, 188, 191, 192, 197, 263.<br>**β2m:** 13, 31, 51, 84.<br>**CDRα:** 112.<br>**CDRβ:** 27, 45, 134, 164, 204.
**1G4_c58c61**<br>PDB: 2P5E	**HLA:** 3, 70, 74, 93, 114, 145, 260.<br>**β2m:**<br>**CDRα:**<br>**CDRβ:** 151.	**HLA:** 151, 188, 191, 192, 197, 263.<br>**β2m:** 13, 31, 51, 84.<br>**CDRα:** 112.<br>**CDRβ:** 27, 45, 134, 164, 204.

Supplementary Table S3. PDB IDs and histidine tautomerisation state assignments for the MD simulations of the A6, DMF5 and MEL5 and affinity enhanced TCRs.
[a], All Apo-TCR simulations used the same tautomerisation states as those in the TCR-pHLA simulations.
[b], HID corresponds to a histidine residue which is singly protonated on its Nδ1 nitrogen.
[c], HIE corresponds to a histidine residue which is singly protonated on their Nε2 nitrogen.

TCR-pHLA System[a]	HID Tautomerisation States[b]	HIE Tautomerisation States[c]
**DMF5**<br>PDB: 3QDG	**HLA:** 3, 70, 74, 93, 114, 151, 191, 192, 260.<br>**β2m:** 51.<br>**CDRα:**<br>**CDRβ:** 32, 50, 138, 168.	**HLA:** 145, 188, 197, 263.<br>**β2m:** 13, 31, 84.<br>**CDRα:**<br>**CDRβ:** 155, 208.
**DMF5_YW**<br>PDB: 4L3E	**HLA:** 3, 70, 74, 93, 114, 151, 191, 192, 260.<br>**β2m:** 51.<br>**CDRα:**<br>**CDRβ:** 32, 50, 138, 168.	**HLA:** 145, 188, 197, 263.<br>**β2m:** 13, 31, 84.<br>**CDRα:**<br>**CDRβ:** 155, 208.
**A6**<br>PDB: 1AO7	**HLA:** 3, 70, 74, 93, 114.<br>**β2m:**<br>**CDRα:**<br>**CDRβ:**	**HLA:** 145, 151, 188, 191, 192, 260, 263.<br>**β2m:** 13,31, 51, 84.<br>**CDRα:**<br>**CDRβ:** 29, 47, 139, 156, 169, 209.
**A6_c134**<br>PDB: 4FTV	**HLA:** 3, 70, 74, 93, 114.<br>**β2m:**<br>**CDRα:**<br>**CDRβ:**	**HLA:** 145, 151, 188, 191, 192, 260, 263.<br>**β2m:** 13,31, 51, 84.<br>**CDRα:**<br>**CDRβ:** 29, 47, 139, 156, 169, 209.
**MEL5**<br>PDB: 3HG1	**HLA:** 3, 70, 74, 114.<br>**β2m:**<br>**CDRα:**<br>**CDRβ:** 5, 207.	**HLA:** 93, 145, 151, 188, 191, 192, 197, 260, 263.<br>**β2m:** 13, 31, 51, 84.<br>**CDRα:** 71.<br>**CDRβ:** 137, 154, 167.
**MEL5_α24β17**<br>PDB: 4JFF	**HLA:** 3, 70, 74, 114.<br>**β2m:**<br>**CDRα:**<br>**CDRβ:** 5, 207.	**HLA:** 93, 145, 151, 188, 191, 192, 197, 260, 263.<br>**β2m:** 13, 31, 51, 84.<br>**CDRα:** 71.<br>**CDRβ:** 137, 154, 167.

Supplementary Table S4: Average number of vdWs contacts formed between the TCR to each pHLA residue for all 1G4 simulations. For each TCR-pHLA complex the 10 pHLA residues with the greatest number of contacts are coloured red, with their rank provided in brackets.

	TCR-pHLA Complex				
**Residue**	**1G4**	**1G4_c5c1**	**1G4_c49c50**	**1G4_c58c62**	**1G4_c58c61**
**HLA: 19**	0.0	0.0	0.2	0.3	0.7
**HLA: 62**	1.4	0.7	1.5	0.5	0.6
**HLA: 65**	14.6 (4)	19.2 (3)	16.3 (4)	14.3 (4)	19.5 (3)
**HLA: 66**	4.2	6.0 (9)	2.4	6.1 (9)	5.6 (10)
**HLA: 68**	2.4	2.1	4.2	3.7	3.7
**HLA: 69**	2.5	3.7	2.4	4.2	3.5
**HLA: 71**	0.0	0.0	0.1	0.1	0.0
**HLA: 72**	11.6 (6)	13.3 (4)	16.9 (3)	15.4 (3)	14.8 (4)
**HLA: 73**	4.0	5.9 (10)	4.7 (10)	6.0 (10)	6.0 (9)
**HLA: 75**	1.7	0.5	3.6	1.8	2.0
**HLA: 76**	0.8	1.9	1.4	1.9	1.9
**HLA: 146**	0.1	0.0	0.0	0.2	0.1
**HLA: 149**	0.0	0.0	0.0	0.3	0.2
**HLA: 150**	7.1 (9)	5.0	4.0	2.4	2.4
**HLA: 151**	4.9 (10)	5.0	6.5 (8)	5.2	4.8
**HLA: 152**	0.3	0.0	0.0	0.0	0.0
**HLA: 154**	2.1	2.1	1.4	4.0	4.9
**HLA: 155**	16.4 (3)	11.9 (6)	11.5 (6)	9.9 (6)	11.7 (6)
**HLA: 158**	0.0	0.0	0.7	0.4	0.6
**HLA: 163**	0.0	1.0	0.0	0.7	1.0
**Pep: 4**	21.1 (2)	27.8 (2)	21.5 (2)	28.3 (2)	28.0 (2)
**Pep: 5**	44.4 (1)	42.7 (1)	44.4 (1)	42.4 (1)	42.8 (1)
**Pep: 6**	9.4 (7)	7.8 (7)	9.5 (7)	7.7 (7)	7.7 (7)
**Pep: 7**	7.1 (8)	7.4 (8)	6.2 (9)	7.3 (8)	6.7 (8)
**Pep: 8**	12.5 (5)	13.1 (5)	12.0 (5)	12.7 (5)	12.7 (5)

Supplementary Table S5: Average number of hydrogen bonds formed between the TCR to each pHLA residue for all 1G4 simulations. For each TCR-pHLA complex the 10 pHLA residues with the greatest number of contacts are coloured red, with their rank provided in brackets.

	TCR-pHLA Complex				
**Residue**	**1G4**	**1G4_c5c1**	**1G4_c49c50**	**1G4_c58c62**	**1G4_c58c61**
**HLA: 19**	0.1	0.3	0.2	0.2	0.4
**HLA: 43**	0.0	0.0	0.1	0.1	0.0
**HLA: 58**	0.0	0.1	0.0	0.1	0.1
**HLA: 61**	0.0	0.0	0.1	0.0	0.1
**HLA: 65**	2.3 (2)	3.2 (1)	2.3 (2)	2.9 (1)	3.2 (1)
**HLA: 66**	0.5	0.7	0.4	0.7 (10)	0.7
**HLA: 68**	0.4	0.6	0.5 (10)	0.4	0.6
**HLA: 70**	0.0	0.1	0.0	0.0	0.1
**HLA: 71**	0.0	0.0	0.0	0.1	0.0
**HLA: 72**	1.7 (4)	1.1 (8)	2.2 (3)	1.0 (7)	0.9 (8)
**HLA: 73**	0.6	1.6 (5)	0.7 (9)	1.6 (4)	1.6 (5)
**HLA: 75**	0.4	0.3	0.4	0.3	0.2
**HLA: 80**	0.0	0.0	0.0	0.1	0.0
**HLA: 146**	0.0	0.3	0.3	0.4	0.3
**HLA: 149**	0.1	0.1	0.1	0.2	0.2
**HLA: 150**	1.5 (6)	1.1 (7)	0.2	0.5	0.6
**HLA: 151**	1.0 (8)	0.8	0.3	0.7	0.7 (10)
**HLA: 154**	0.2	0.3	0.0	0.4	0.4
**HLA: 155**	2.1 (3)	1.8 (4)	1.2 (5)	1.2 (5)	1.6 (4)
**HLA: 163**	0.1	0.0	0.0	0.0	0.0
**Pep: 1**	0.0	0.1	0.0	0.0	0.1
**Pep: 4**	1.1 (7)	1.4 (6)	1.0 (6)	1.2 (6)	1.3 (6)
**Pep: 5**	1.0 (9)	0.9 (10)	0.9 (8)	0.9 (9)	0.9 (9)
**Pep: 6**	1.7 (5)	1.8 (3)	1.8 (4)	1.8 (3)	1.8 (3)
**Pep: 7**	1.0 (10)	1.0 (9)	1.0 (7)	1.0 (8)	1.0 (7)
**Pep: 8**	2.5 (1)	2.6 (2)	2.5 (1)	2.6 (2)	2.6 (2)

Supplemetary Table S6: Average number of vdWs contacts formed between the TCR to each pHLA residue for our simulations of wildtype and affinity enhanced DMF5, MEL5 and Tax A6. For each TCR-pHLA complex the 10 pHLA residues with the greatest number of contacts are coloured red, with their rank provided in brackets.

	TCR-pHLA Complex					
**Residue**	**DMF5**	**DMF5_ YW**	**MEL5**	**MEL5_ α24β17**	**A6**	**A6_ c134**
**HLA: 43**	0.0	0.0	0.4	0.0	0.0	0.0
**HLA: 55**	3.0	3.5	0.0	0.0	2.7	3.6
**HLA: 56**	0.0	0.2	0.0	0.0	0.0	0.0
**HLA: 58**	0.1	2.9	0.3	2.0	0.1	0.5
**HLA: 59**	1.2	2.3	0.0	0.3	0.4	1.0
**HLA: 61**	0.0	0.9	0.0	0.0	0.0	0.0
**HLA: 62**	0.0	3.2	1.6	0.2	0.0	0.0
**HLA: 63**	0.0	0.3	0.0	0.0	0.0	0.0
**HLA: 65**	7.1 (4)	8.5 (3)	13.9 (1)	19.9 (2)	17.1 (3)	17.1 (2)
**HLA: 66**	4.1 (9)	8.3 (4)	1.8	4.0	8.3 (6)	9.1 (6)
**HLA: 68**	0.0	0.0	2.2	1.6	2.7	2.8
**HLA: 69**	5.6 (7)	7.1 (5)	3.6 (9)	3.1	5.7 (8)	5.7 (9)
**HLA: 70**	0.1	0.5	2.3	1.4	0.0	0.0
**HLA: 72**	10.4 (1)	12.2 (1)	8.0 (4)	25.4 (1)	2.2	2.5
**HLA: 73**	1.3	1.5	2.9	1.7	2.2	2.1
**HLA: 75**	1.3	1.7	0.9	6.6 (8)	0.0	0.0
**HLA: 76**	1.7	2.1	1.3	4.2 (10)	0.0	0.0
**HLA: 145**	0.0	0.1	0.0	0.0	0.0	0.0
**HLA: 146**	0.0	0.8	0.0	0.0	0.0	0.0
**HLA: 149**	0.0	0.7	0.0	0.0	0.8	0.2
**HLA: 150**	0.8	4.8 (9)	0.0	0.1	5.1 (9)	8.4 (8)
**HLA: 151**	0.2	0.0	1.0	0.7	3.5	4.6 (10)
**HLA: 152**	0.0	0.0	0.0	0.0	0.5	1.2
**HLA: 154**	0.1	0.6	4.2 (8)	6.3 (9)	1.2	1.9
**HLA: 155**	7.6 (3)	5.8 (8)	9.2 (2)	10.4 (3)	13.3 (4)	12.9 (4)
**HLA: 157**	0.0	0.0	0.0	8.0 (6)	0.5	0.1
**HLA: 158**	2.1	1.8	1.4	2.2	2.3	1.6
**HLA: 159**	1.9	1.6	0.7	1.4	0.9	0.8
**HLA: 161**	0.0	0.0	0.0	2.2	0.0	0.1
**HLA: 162**	0.0	0.0	0.0	0.3	0.0	0.1
**HLA: 163**	1.7	2.0	3.5 (10)	2.8	2.4	2.6
**HLA: 166**	1.0	1.8	4.3 (7)	0.4	4.2 (10)	3.6
**HLA: 167**	1.4	2.7	0.3	4.2	2.2	2.0
**HLA: 170**	0.7	1.7	1.5	0.7	2.1	1.0
**Pep: 1**	6.5 (5)	6.1 (6)	0.8	4.2	2.8	3.2
**Pep: 2**	2.3	2.0	0.4	1.2	1.6	1.2
**Pep: 3**	1.7	1.7	1.2	2.1	0.2	0.0
**Pep: 4**	8.4 (2)	9.1 (2)	5.5 (5)	8.7 (5)	9.5 (5)	8.9 (7)
**Pep: 5**	5.9 (6)	6.1 (7)	8.1 (3)	6.9 (7)	25.5 (1)	26.8 (1)
**Pep: 6**	1.9	0.1	2.3	2.9	3.0	2.7
**Pep: 7**	4.2 (8)	4.3 (10)	5.1 (6)	9.3 (4)	7.4 (7)	9.3 (5)
**Pep: 8**	2.7	0.0	1.8	3.0	17.2 (2)	17.1 (3)
**Pep: 9**	4.0 (10)	0.0	0.2	2.1	0.0	0.0

Supplementary Table S7: Average number of hydrogen bonds formed between the TCR to each pHLA residue for our simulations of wildtype and enhanced affinity DMF5, MEL5 and Tax A6. For each TCR-pHLA complex the 10 pHLA residues with the greatest number of contacts are coloured red, with their rank provided in brackets.

	TCR-pHLA Complex					
**Residue**	**DMF5**	**DMF5_ YW**	**MEL5**	**MEL5_ α24β17**	**A6**	**A6_ c134**
**HLA: 19**	0.0	0.0	0.1	0.0	0.0	0.0
**HLA: 43**	0.0	0.0	0.2	0.0	0.0	0.0
**HLA: 55**	1.0 (5)	1.1 (5)	0.0	0.0	0.9 (9)	1.1 (8)
**HLA: 58**	0.3	1.0 (6)	0.1	0.8	0.5	0.5
**HLA: 59**	0.0	0.2	0.0	0.0	0.0	0.0
**HLA: 61**	0.1	0.3	0.1	0.3	0.0	0.0
**HLA: 62**	0.0	0.1	0.0	0.0	0.0	0.0
**HLA: 65**	1.5 (4)	1.6 (3)	2.1 (1)	2.0 (2)	2.5 (1)	2.6 (1)
**HLA: 66**	0.6 (10)	0.9 (9)	0.0	0.7	1.0 (7)	1.3 (5)
**HLA: 68**	0.1	0.0	0.5 (8)	1.0 (9)	0.0	0.0
**HLA: 72**	2.0 (1)	2.4 (1)	1.5 (2)	2.7 (1)	0.2	0.2
**HLA: 73**	0.1	0.2	0.6 (7)	0.0	0.0	0.0
**HLA: 75**	0.5	0.5	0.2	0.0	0.0	0.0
**HLA: 145**	0.0	0.1	0.0	0.0	0.0	0.0
**HLA: 146**	0.1	0.2	0.0	0.0	0.0	0.0
**HLA: 149**	0.0	0.2	0.0	0.0	0.4	0.2
**HLA: 150**	0.0	0.1	0.0	0.1	0.4	0.8
**HLA: 151**	0.0	0.1	0.0	0.2	0.4	0.8
**HLA: 154**	0.1	0.0	0.3	1.3 (6)	0.3	0.2
**HLA: 155**	0.7 (8)	0.9 (7)	0.5 (9)	0.8 (10)	1.0 (6)	1.5 (4)
**HLA: 157**	0.0	0.0	0.0	1.1 (8)	0.1	0.1
**HLA: 158**	0.2	0.2	0.0	0.2	0.3	0.2
**HLA: 161**	0.1	0.1	0.0	1.1 (7)	0.2	0.3
**HLA: 162**	0.0	0.0	0.0	0.1	0.0	0.0
**HLA: 163**	0.5	0.5	0.4 (10)	0.6	0.9 (8)	0.9 (10)
**HLA: 166**	0.4	0.8	0.9	0.1	1.4 (4)	1.1 (9)
**HLA: 167**	0.4	0.3	0.0	0.5	0.5	0.5
**HLA: 170**	0.1	0.0	0.4	0.0	0.1	0.1
**Pep: 1**	1.7 (3)	1.8 (2)	0.6 (6)	1.5 (4)	0.0	0.0
**Pep: 2**	0.7 (9)	0.5	0.2	0.4	0.7	0.5
**Pep: 3**	0.0	0.1	0.0	0.0	0.0	0.0
**Pep: 4**	1.8 (2)	1.3 (4)	1.2 (3)	1.4 (5)	2.2 (3)	1.9 (3)
**Pep: 5**	0.2	0.1	0.1	0.0	1.1 (5)	1.1 (7)
**Pep: 6**	0.1	0.0	0.2	0.1	0.9 (10)	1.2 (6)
**Pep: 7**	0.7 (7)	0.8 (10)	0.9 (5)	1.6 (3)	2.3 (2)	2.1 (2)
**Pep: 9**	0.8 (6)	0.9 (8)	0.4	0.2	0.0	0.0

Supplementary Methods

Cloning, expression and refolding of proteins. The TCR α and TCR β-chains, as well as the HLA class I α-chains (tagged and not tagged with a biotinylation sequence) and β2m sequences, were cloned into the pGMT7 expression vector under the control of the T7 promoter using BamH1 and EcoR1 restriction sites, as described previously[1–3]. All sequences were confirmed by automated DNA sequencing. The TCR α and TCR β-chains, the HLA-A*02:01 α chains and β2m were expressed separately, without post-translational modification, as insoluble inclusion bodies in competent Rosetta DE3 *Escherichia coli* cells using 1 mM IPTG, as described previously[1–3]. For a 1 L TCR refold, 30 mg TCR α-chain was incubated at 37 °C for 30 min with 10 mM DTT and added to cold refold buffer (50 mM TRIS pH 8.1, 2 mM EDTA, 2.5 M urea, 6 mM cysteamine hydrochloride and 4 mM cystamine). After 30 min, 30 mg TCR β-chain, also incubated at 37°C for 30 min with 10 mM DTT, was added. For a 1 L pHLA-I refold, 30 mg HLA-A*02:01 α-chain was mixed with 30 mg β2m and 4 mg of peptide at 37 °C for 30 min with 10 mM DTT. This mixture was then added to cold refold buffer (50 mM TRIS pH 8.1, 2 mM EDTA, 400 mM L-arginine, 6 mM cysteamine hydrochloride, and 4 mM cystamine). TCR and pHLAI refolds were mixed at 4 °C for >1 hr and dialyzed against 10mM TRIS pH 8.1 until the conductivity of the refolds was <2 mS/cm. All the refolds were then filtered, ready for purification. Refolded proteins were purified, initially by ion exchange using a Poros50HQTM column (Thermo Fisher Scientific Inc, MA, USA.), and finally gel filtered into crystallization buffer (10 mM TRIS pH 8.1 and 10 mM NaCl) or BIAcore buffer (10 mM HEPES pH 7.4, 150 mM NaCl, 3 mM EDTA and 0.005% (v/v) surfactant P20) using a Superdex200HRTM column (GE Healthcare, Buckinghamshire, U.K.). Protein quality, either under non-reducing or reducing conditions, was analyzed by Coomassie-stained SDS-PAGE.

Molecular Dynamics Simulations. Starting structures for all simulations were obtained from previously solved X-ray crystal structures from multiple studies (**Table 1 and Supplementary Tables S2&S3**), with any missing residues added using Modeller[4]. Molecular dynamics (MD) simulations of apo-TCR structures were initiated from the TCR-pHLA bound structure. MolProbity[5] was used to determine the optimum tautomerisation states for all histidine residues and make any required Asn/Gln side chain flips under the criteria of optimising the hydrogen bonding network. Protonation states of all titratable residues were assigned using PropKa 3.0[6] for pH 7. All structures were then solvated in an octahedral water box (retaining any crystal waters), ensuring all protein atoms were at least 10 Å away from the box boundary, with Na^+ or Cl^- counter ions added, as necessary, to ensure an overall neutral charge. MD simulations were performed using GPU accelerated Amber16[7], with the ff14SB force field[8] and TIP3P water model used to describe protein and water molecules, respectively. All systems investigated were equilibrated to 300 K and 1 atm in the NPT ensemble (see section “MD Equilibration Procedure” below). Subsequently, production MD simulations were performed for 100 ns each with 10 replicas performed per TCR-pHLA or apo-TCR structure. Production MD simulations were run using a 2 fs time step with the SHAKE algorithm applied to any bond containing a hydrogen atom. An 8 Å direct space non-bonded cut-off was applied with long range electrostatics evaluated using the particle mesh Ewald algorithm[9]. Temperature was regulated using Langevin temperature control (collision frequency of 1 ps^{-1}), whilst pressure was controlled with a Berendsen barostat (setting the pressure relaxation time to 1 ps).

MD Equilibration Procedure. The following procedure was used to prepare all systems simulated for production MD simulations at 300 K and 1 atm. Furthermore, the equilibration protocol used is identical for both our "long" timescale (5 x 100 ns) and "short" time scale (25 x 4 ns) MD simulations. All dynamics steps applied the SHAKE algorithm to constrain all bonds containing hydrogen. Replicas simulations were initiated from the second heating step of the following protocol (with each replica therefore assigned different random velocity vectors at this stage). First hydrogens atoms and solvent molecules were energy minimised (using 500 steps of steepest descent followed by 500 steps of conjugate gradient minimisation). To prevent the movement of non-hydrogen and non-solvent atoms during the minimisation, 10 kcal mol^{-1} $Å^{-1}$ positional restraints were used to keep all heavy atoms fixed. Then the solvent was heated rapidly from 50 K to 300 K (NVT ensemble, 1 fs timestep) over the course of 200 ps, with the previously described restraints still maintained. The positional restraints were then replaced with 5 kcal mol^{-1} $Å^{-1}$ positional restraint on only the Cα carbon atoms and subjected to another round of energy minimisation (500 steps of steepest descent followed by 500 steps of conjugate gradient). Retaining these positional restraints, the system was heated from 25 K to 300 K over the course of 50 ps (NVT ensemble, 1 fs time step). Simulations were then performed in the NPT ensemble (1 atm, 300 K, 2 fs time step) by first gradually reducing the 5 kcal mol^{-1} $Å^{-1}$ Cα carbon restraints over the course of 50 ps. This was done by reducing the restraint weight by 1 kcal mol^{-1} $Å^{-1}$ every 10 ps. The end structure from this run was then used as the starting structure for production MD simulations. Simulations performed in the NVT ensemble used Langevin temperature control (with a collision frequency of 1 ps^{-1}) and used a simulation timestep of 1 fs. Simulations performed in the NPT ensemble again used Langevin temperature control (collision frequency of 1 ps^{-1}) and a Berendsen barostat (1 ps pressure relaxation time).

MD Simulation Analysis. Trajectory analysis was performed with CPPTRAJ[10], using frames collected every 10 ps for analysis of MD simulations. Hydrogen bonds (HBs), including water bridged HBs between atoms of the TCR and pHLA were defined as formed if the donor acceptor distance was within 3.5 Å and the donor hydrogen acceptor angle was 180 ± 45°. If two heavy (non-hydrogen) atoms were within 4 Å of one another, a van der Waals (vdWs) contact was considered to be formed between the two atoms. RMSF calculations were performed on the Cα atoms of the relevant residues with RMS fitting performed to a consistent set of TCR residues in the variable domains that are not highly flexible (see section "RMS Fitting Procedure" below). RMSFs, HBs and vdWs contacts were calculated by discarding the first 10 ns of simulation time (meaning 10 replicas of 10-100 ns used) to allow for structure equilibration. Buried solvent accessible surface area (BSASA) was determined using with the LCPO algorithm[11], available with the "molsurf" command within CPPTRAJ[10].

RMS Fitting Procedure. To ensure fair comparison between the TCR structures, RMS fitting (for RMSF calculations) was performed using the same set of residues in the TCR variable region. Residues excluded from the RMS fitting procedure were the first five N-terminal residues and all CDR loop residues (due to their high mobility, which would therefore provide a poor fit). Residues used for RMS fitting from Chain A were therefore: 6-22,33-46,55-65,73-94,103-113. Residues used for RMS fitting from Chain B were: 6-21,30-47,54-65,73-91,102-111. RMS fitting of MD simulation snapshots was first performed to the crystal structure, with this RMS fitted trajectory used to create an average structure. Following this, all snapshots were then re-fitted to the average structure for the subsequent RMSF calculation.

MMGBSA Methodology. The molecular mechanics generalized Born surface area (MMGBSA) method is a binding free energy calculation method which has been widely used to predict relative binding free energies[12,13]. The approach uses a combination of MD simulations (for sampling to obtain many snapshots) and empirical calculations (on the obtained snapshots) to predict ΔG_{bind}. In the MMGBSA approach, the different contributions to affinity are calculated individually and summed together to obtain ΔG_{bind} (see Equation 1):

Equation S1: $$\Delta G_{\text{bind}} = \Delta E_{\text{el}} + \Delta E_{\text{vdw}} + \Delta G_{\text{pol}} + \Delta G_{\text{npol}} - T\Delta S \qquad (1)$$

Where ΔE_{el} and ΔE_{vdw} are obtained directly from the molecular mechanics force field terms and describe the gas phase interaction energy. The polar and non-polar contributions to the solvation free energy are described by ΔG_{pol} and ΔG_{npol}, respectively. ΔG_{pol} is calculated by solving the GB equation, whilst ΔG_{npol} is obtained from a function that assumes a linear relationship between the solvent accessible surface area and ΔG_{npol}. Finally, $T\Delta S$ describes the change in entropy of the solute upon binding, most often calculated through normal mode analysis (NMA). For large systems like TCR-pHLA, NMA is computationally expensive and also tends to produce large errors that do not improve the accuracy of the calculation[14]. Furthermore, as it is not possible to decompose the results from NMA to a per-residue level, we did not perform NMA for our MMGBSA calculations.

MMGBSA Calculations. MMGBSA calculations were performed using MMPBSA.py.MPI[15], using 25 independent (random velocity vectors assigned upon heating) 4 ns long MD simulations (separate to the above described 100 ns long simulations). These simulations were run under the same conditions as the aforementioned longer timescale simulations. From each run, 300 equally spaced snapshots were taken from the last 3 ns of each MD simulation for MMGBSA calculations, giving a total of 7500 frames per complex. MMGBSA calculations used the GB-Neck2 (i.e. igb = 8) solvation model and an implicit salt concentration of 150 mM. The obtained results were decomposed into their per-residue contributions to the total free energy, with the values obtained used to calculate the differences between the WT and eaTCRs.

Supplementary References

1. Boulter, JM, Glick, M, Todorov, PT, Baston, E, Sami, M, Rizkallah, P, *et al.* (2003). Stable, soluble T-cell receptor molecules for crystallization and therapeutics. *Protein Eng.* **16**: 707–11.
2. Garboczi, DN, Utz, U, Ghosh, P, Seth, A, Kim, J, VanTienhoven, EA, *et al.* (1996). Assembly, specific binding, and crystallization of a human TCR-alphabeta with an antigenic Tax peptide from human T lymphotropic virus type 1 and the class I MHC molecule HLA-A2. *J. Immunol.* **157**: 5403–10.
3. Garboczi, DN, Hung, DT and Wiley, DC (1992). HLA-A2-peptide complexes: refolding and crystallization of molecules expressed in Escherichia coli and complexed with single antigenic peptides. *Proc. Natl. Acad. Sci. U. S. A.* **89**: 3429–3433.
4. Webb, B and Sali, A (2014). Comparative Protein Structure Modeling Using MODELLER. *Curr. Protoc. Bioinforma.* **47**: 5.6.1-5.6.32.
5. Chen, VB, Arendall, WB, Headd, JJ, Keedy, DA, Immormino, RM, Kapral, GJ, *et al.* (2010). *MolProbity* : all-atom structure validation for macromolecular crystallography. *Acta Crystallogr. Sect. D Biol. Crystallogr.* **66**: 12–21.
6. Søndergaard, CR, Olsson, MHM, Rostkowski, M and Jensen, JH (2011). Improved Treatment of Ligands and Coupling Effects in Empirical Calculation and Rationalization of pKa Values. *J. Chem. Theory Comput.* **7**: 2284–95.
7. D.A. Case, D.S. Cerutti, T.E. Cheatham, III, T.A. Darden, R.E. Duke, T.J. Giese, H. Gohlke, A.W. Goetz, D. Greene, N. Homeyer, S. Izadi, A. Kovalenko, T.S. Lee, S. LeGrand, P. Li, C. Lin, J. Liu, T. Luchko, R. Luo, D. Mermelstein, K.M. Merz, G. Monard, H., DMY and PAK (2017). AMBER 2016. *Univ. Calafornia San Fr.*
8. Maier, JA, Martinez, C, Kasavajhala, K, Wickstrom, L, Hauser, KE and Simmerling, C (2015). ff14SB: Improving the Accuracy of Protein Side Chain and Backbone Parameters from ff99SB. *J. Chem. Theory Comput.* **11**: 3696–713.
9. Darden, T, York, D and Pedersen, L (1993). Particle mesh Ewald: An *N* ·log(*N*) method for Ewald sums in large systems. *J. Chem. Phys.* **98**: 10089–10092.
10. Roe, DR and Cheatham, TE (2013). PTRAJ and CPPTRAJ: Software for Processing and Analysis of Molecular Dynamics Trajectory Data. *J. Chem. Theory Comput.* **9**: 3084–3095.
11. Weiser, J, Shenkin, PS and Still, WC (1999). Approximate atomic surfaces from linear combinations of pairwise overlaps (LCPO). *J. Comput. Chem.* **20**: 217–230.
12. Genheden, S and Ryde, U (2015). The MM/PBSA and MM/GBSA methods to estimate ligand-binding affinities. *Expert Opin. Drug Discov.* **10**: 449–461.
13. Chen, F, Liu, H, Sun, H, Pan, P, Li, Y, Li, D, *et al.* (2016). Assessing the performance of the MM/PBSA and MM/GBSA methods. 6. Capability to predict protein–protein binding free energies and re-rank binding poses generated by protein–protein docking. *Phys. Chem. Chem. Phys.* **18**: 22129–22139.
14. Sun, H, Duan, L, Chen, F, Liu, H, Wang, Z, Pan, P, *et al.* (2018). Assessing the performance of MM/PBSA and MM/GBSA methods. 7. Entropy effects on the performance of end-point binding free energy calculation approaches. *Phys. Chem. Chem. Phys.*doi:10.1039/C7CP07623A.
15. Miller, BR, McGee, TD, Swails, JM, Homeyer, N, Gohlke, H and Roitberg, AE (2012). *MMPBSA.py* : An Efficient Program for End-State Free Energy Calculations. *J. Chem. Theory Comput.* **8**: 3314–3321.
